# Validation study of case-identifying algorithms for severe hypoglycemia using hospital administrative data in Japan

**DOI:** 10.1371/journal.pone.0289840

**Published:** 2023-08-09

**Authors:** Satoshi Osaga, Takeshi Kimura, Yasuyuki Okumura, Rina Chin, Makoto Imori, Machiko Minatoya

**Affiliations:** 1 Japan Drug Development and Medical Affairs, Eli Lilly Japan K.K., Kobe, Hyogo Prefecture, Japan; 2 Real World Data Co., Ltd., Nakagyo Ward, Kyoto, Kyoto Prefecture, Japan; Karolinska Institutet, SWEDEN

## Abstract

**Objective:**

The purpose of this study was to evaluate the performance of algorithms for identifying cases of severe hypoglycemia in Japanese hospital administrative data.

**Methods:**

This was a multicenter, retrospective, observational study conducted at 3 acute-care hospitals in Japan. The study population included patients aged ≥18 years with diabetes who had an outpatient visit or hospital admission for possible hypoglycemia. Possible cases of severe hypoglycemia were identified using health insurance claims data and Diagnosis Procedure Combination data. Sixty-one algorithms using combinations of diagnostic codes and prescription of high concentration (≥20% mass/volume) injectable glucose were used to define severe hypoglycemia. Independent manual chart reviews by 2 physicians at each hospital were used as the reference standard. Algorithm validity was evaluated using standard performance metrics.

**Results:**

In total, 336 possible cases of severe hypoglycemia were identified, and 260 were consecutively sampled for validation. The best performing algorithms included 6 algorithms that had sensitivity ≥0.75, and 6 algorithms that had positive predictive values ≥0.75 with sensitivity ≥0.30. The best-performing algorithm with sensitivity ≥0.75 included any diagnoses for possible hypoglycemia or prescription of high-concentration glucose but excluded suspected diagnoses (sensitivity: 0.986 [95% confidence interval 0.959–1.013]; positive predictive value: 0.345 [0.280–0.410]). Restricting the algorithm definition to those with both a diagnosis of possible hypoglycemia and a prescription of high-concentration glucose improved the performance of the algorithm to correctly classify cases as severe hypoglycemia but lowered sensitivity (sensitivity: 0.375 [0.263–0.487]; positive predictive value: 0.771 [0.632–0.911]).

**Conclusion:**

The case-identifying algorithms in this study showed moderate positive predictive value and sensitivity for identification of severe hypoglycemia in Japanese healthcare data and can be employed by future pharmacoepidemiological studies using Japanese hospital administrative databases.

## Introduction

The current guidelines of the American Diabetes Association define severe hypoglycemia as an event characterized by altered mental and/or physical functioning that requires assistance from another person for recovery [1]. Although there are several causes of hypoglycemia, the majority of cases among patients with diabetes arise from use of glucose-lowering therapies, particularly insulins and sulfonylureas [2, 3]. Severe hypoglycemia is a serious complication of diabetic therapy, associated with increased risk of mortality and morbidity [4–8] and significant socioeconomic costs, including the frequent requirement for emergency department visits and hospitalization [9].

Real-world healthcare databases are increasingly being used in pharmacoepidemiological and post-marketing surveillance studies as they contain data from large, heterogeneous populations of patients which can be queried with coded algorithms designed to identify specific populations, such as patients with certain medical conditions, characteristics, or outcomes [10]. Use of electronic databases can offer time- and cost-saving advantages over conventional methods and has wide applicability, including the assessment of real-world drug safety and effectiveness, costs, resource utilization, and treatment patterns [10–12]. However, the algorithms used to interrogate administrative databases are based on diagnostic codes, and the codes and databases were not designed for this purpose. As such, validation studies are needed to evaluate the performance of algorithms to correctly detect cases and outcomes of interest in the target database [10–12].

The accuracy of case-identifying algorithms in real-world databases can be assessed by comparing algorithm performance against a reference standard, which is the best available method for detecting the condition of interest [13]. Several prior studies have assessed the performance of algorithms that identify cases of hypoglycemia requiring in- and/or outpatient visits to North American hospitals [14–18], but similar validation studies have not been conducted in Japan. Prior studies have indicated racial or ethnic differences in hypoglycemia-related emergency department visits and hospitalizations [19]. In addition, country- and health data system-specific factors have been found to limit the generalizability of validation study results across different diseases [20–23]. For these reasons, there is a need to develop and assess high-performing algorithms within the Japanese healthcare context. The primary objective of this study was to evaluate the validity of case-identifying algorithms for severe hypoglycemia using administrative data at 3 acute-care hospitals in Japan.

## Methods

### Study overview

This was a multicenter, retrospective observational study that investigated the validity of case-identifying algorithms for the detection of severe hypoglycemia using Japanese hospital administrative data. The algorithms were based on diagnosis or prescription for possible hypoglycemia, defined using the International Statistical Classification of Diseases and Related Health Problems, 10th Revision (ICD-10) [24]. As ICD-10 codes do not include a code specific for “severe hypoglycemia”, the current study examined all possible hypoglycemic events that required inpatient or outpatient visits to participating hospitals to identify cases of likely severe hypoglycemia.

**Fig 1:**
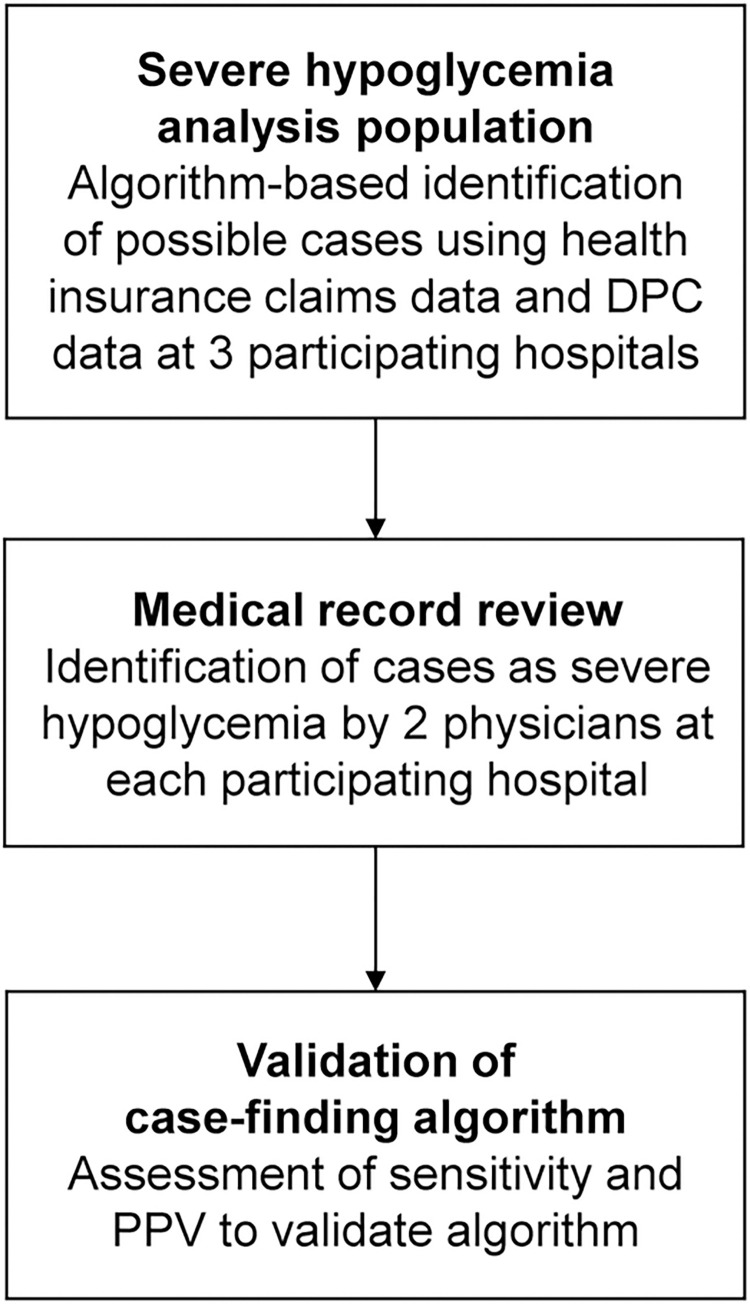
Study design. DPC = Diagnosis Procedure Combination; PPV = positive predictive value.

An overview of the study design is shown in Fig 1. Eli Lilly Japan K.K. commissioned the Medical Data Vision Co., Ltd, (MDV; Tokyo, Japan) to identify candidate hospitals using the commercially available MDV database, an anonymized database containing data from health insurance claims and discharge summaries from acute-care hospitals under the Diagnosis Procedure Combination (DPC) system in Japan [25]. All possible cases of severe hypoglycemia were identified at 3 participating hospitals using a broad case-identifying algorithm based on health insurance claims data and DPC data stored at the hospitals. Following case selection at all participating hospitals, possible cases were subsequently sampled in consecutive fashion from newest to oldest for medical record review, which was conducted independently by 2 raters at each participating hospital based on review of medical record information. Raters were physicians who had experience in emergency department or inpatient diabetes treatment. Raters reviewed non-anonymized hospital record data but were blinded to health insurance claims data, DPC data, and algorithmic results. The anonymized results of medical record review were provided to MDV only. MDV calculated the positive predictive value (PPV) and sensitivity for each algorithm and provided the results of statistical analyses to Eli Lilly K.K. and Real World Data Co., Ltd, (Kyoto, Japan) for analysis and interpretation. Eli Lilly K.K. calculated additional performance metrics (prevalence, probability+, and probability-) using the results of the statistical analyses provided by MDV. Authors did not receive any patient-level data. Data collection at the participating hospitals was conducted from May 20 to June 24, 2022.

This study was approved by an independent central ethics committee (MINS Research Ethics Committee, Tokyo, Japan; Application ID#220202) and conducted in accordance with the Declaration of Helsinki and the Japanese Ethical Guidelines for Medical and Biological Research Involving Human Subjects [26]. As this was a non-interventional study using anonymized patient data, the requirement for informed consent was waived by the ethical review board. Participating hospitals provided the opportunity for patients to refuse the use of their health information.

### Hospital selection and study population

Hospitals with the most patients recorded in the MDV database meeting the criteria for the study population were identified, and those that had previously participated in validation studies were contacted by MDV to participate in this study. The 3 hospitals that agreed to participate were included in this study. Per agreement between MDV and the hospitals to maintain their anonymity, participating hospitals have been arbitrarily numbered herein as 1, 2, and 3. The representativeness of these hospitals was assessed by comparing the age distribution and the inpatient/outpatient ratio of the potential patients in the participating hospitals to those of potential patients in all medical institutions in the MDV database.

Eligible patients were ≥18 years at the date of an outpatient visit or hospital admission with possible hypoglycemia (the index date) with a diagnosis code of diabetes based on DPC or health claims data within 12 months from the index month and a diagnosis or prescription for possible hypoglycemia at the index date. The definition of possible hypoglycemia was based on the following ICD-10 diagnostic codes [24]: E10.0 (type 1 diabetes mellitus with coma;), E11.0 (type 2 diabetes mellitus with coma), E14.0 (unspecified diabetes mellitus with coma), E15 (non-diabetic hypoglycemic coma), E16.0 (drug-induced hypoglycemia without coma), E16.1 (other hypoglycemia), and E16.2 (hypoglycemia, unspecified) or a high-concentration (≥20% mass/volume) injectable glucose prescription for possible hypoglycemia. Patients with diagnoses or prescriptions recorded after the hospital admission date were not included in order to exclude hypoglycemic events arising secondary to treatment while in hospital.

The eligibility evaluation period was defined in the study protocol as the earliest time point at which data could be obtained to July 31, 2021. During the study, a practical measure was taken to avoid any excessive imbalance in the numbers of patients assessed at the 3 hospitals, wherein the start of the evaluation period was determined for each hospital based on the number of patients who could potentially meet the eligibility criteria at the hospital during the evaluation period. The actual start dates for eligibility evaluation across the 3 hospitals were June 1, 2018; January 1, 2019; and January 1, 2020 (dates are provided in no particular order, i.e., numbering of hospitals does not correspond to the order or timing of the eligibility evaluation periods). For patients who met the above criteria 2 or more times, only the most recent episode was used, and the previous hypoglycemic episodes were excluded.

### Medical record review

For the reference standard, all possible eligible cases were assessed independently by 2 physicians at each participating hospital who had experience in the emergency department or in diabetes treatment. Assessments of medical record information were conducted using a judgement sheet based on the American Diabetes Association definition of severe hypoglycemia (S1 Table). In brief, the criteria were 1) the presence of sympathetic symptoms (finger tremor, palpitation, cold sweat, etc) with no recovery without assistance by family members or medical treatment; 2) the presence of central nervous system symptoms (decreased level of consciousness, abnormal behavior, convulsions, coma, etc) with no recovery without assistance by family members or medical treatment; and 3) interventions for recovery provided by family members or medical institutions (oral intake of drinking water containing glucose, injection of glucagon, intravenous injection of high-concentration [≥20% mass/volume] glucose, etc). A case was judged as severe hypoglycemia if criterion 3 was met with either criterion 1 or 2. If the judgements of the 2 assessors did not match, a third assessor with more experience in treating severe hypoglycemia determined whether the case was severe hypoglycemia. All assessors were blinded from viewing health insurance claims data, DPC data, and algorithmic results. The assessment window for severe hypoglycemia was the period within ±30 days of the index date.

The assessors also evaluated additional patient information in hospital medical records, including blood glucose levels at the time of the event (date, continuous variable/unclear). If multiple glucose values were available for a patient, the minimum value between the event occurrence and the hospital visit was recorded. Additionally, assessors determined whether patients were under sulfonylurea or insulin treatment (no/yes/unclear) at the time of the event, as these glucose-lowering medications have previously been associated with the highest risk for hypoglycemic-related hospital visits [19]. The assessment window for these data was from 30 days before the index date to the index date.

### Algorithms

We identified cases of possible severe hypoglycemia in hospital administrative data by the application of 61 case-identifying algorithms that were based on a combination of disease names for hypoglycemia (ICD-10 codes E10.0, E11.0, E14.0, E15, E16.0, E16.1, and E16.2) and/or prescription of high-concentration (≥20% mass/volume) injectable glucose. The algorithms used herein are listed in S2 Table. ICD-10 diagnostic codes and health insurance drug codes used in the study are listed in full in S3 and S4 Tables, respectively.

### Sample size

Sample size was determined in accordance with a Japanese regulatory notice which states that validation studies plan for at least 100 cases with the target condition within all possible cases [27]. This study planned to consecutively sample 260 patients, on the assumption that at least 100 patients who met the inclusion criteria would be judged to have cases of severe hypoglycemia by medical record review. This assumption was based on a prior study by Min et al. (2019) that found 12 of 15 inpatients with possible hypoglycemia (80%) and 44 of 103 emergency outpatients with possible hypoglycemia (43%) were cases of severe hypoglycemia [17]. A preliminary survey of patients who met the inclusion criteria in the current study showed that there were 62 inpatients and 198 outpatients, which, if the proportions derived from the Min et al. study [17] were applied to the current study, would result in the identification of approximately 50 cases of severe hypoglycemia among the inpatients (i.e., 80% sensitivity) and 85 cases among the outpatients (i.e., 43% sensitivity), or 135 overall.

### Statistical analyses

Selected case information from hospital health insurance claims and DPC data were descriptively summarized, including patient sex, age and age group (<65 years or ≥65 years) at the index date, classification as inpatient or outpatient (including emergency and other outpatients) at the index date, and the Sundararajan version of the Charlson score [28]. For continuous variables, mean with standard deviation or median with interquartile range (IQR) were calculated. For categorical variables, frequency and percentage were summarized.

Case terminology was based on the Standards for Reporting of Diagnostic Accuracy Studies guidelines [29], in which cases with the presence of severe hypoglycemia were termed “cases with the target condition” and cases identified by algorithm (test under evaluation) were termed “index test-positive cases”. Cases were considered: (a) “true positives” if identified as “true” by both the algorithm and medical record review; (b) “false negatives” if identified by medical record review but not by the algorithm; (c) “false positives” if identified by the algorithm but not by medical record review; and (d) “true negatives” if identified as false by both the algorithm and medical record review. In the medical record review, inter-rater agreement was assessed via a Kappa statistic.

As the regulatory agency in Japan recommends PPV and sensitivity as measures of validation for rare outcome events [27], the focus of our validation analysis was on these metrics. Normal approximation to the binomial distribution was used to calculate 95% confidence intervals (CIs) for PPV and sensitivity. We also report prevalence, probability+, and probability-.

S1 Fig shows the calculations for the performance metrics evaluated in this study. PPV was calculated by dividing the number of true-positive cases by the number of index test-positive cases (the sum of the true-positive and false-positive cases). Due to the impractical nature of reviewing all medical records in a database when the prevalence of the target condition is low, for the evaluation of sensitivity, we assumed that all cases with the target condition met the eligibility criteria and were included in the study. Sensitivity was calculated by dividing the number of true-positive cases by the number of cases with the target condition (sum of true-positive and false-negative cases). Prevalence was calculated by dividing the number of cases identified as hypoglycemia by medical record review (sum of the true-positive and false-negative cases) by the total number of cases. Probability+ was calculated by dividing the number of index test-positive cases (sum of the true-positive and false-positive cases) by the total number of cases. Probability- was calculated by dividing the number of index-case negative cases (sum of the true- negative and false-negative cases) by the total number of cases.

Performance metrics were also determined for subgroups, including sex (male or female), age group (<65 years old or ≥65 years), and hospitalization classification (inpatient or outpatient). In these analyses, patients were excluded from the relevant subgroup analysis if values were missing for the variable used for subgrouping.

## Results

### Analysis population

**Fig 2:**
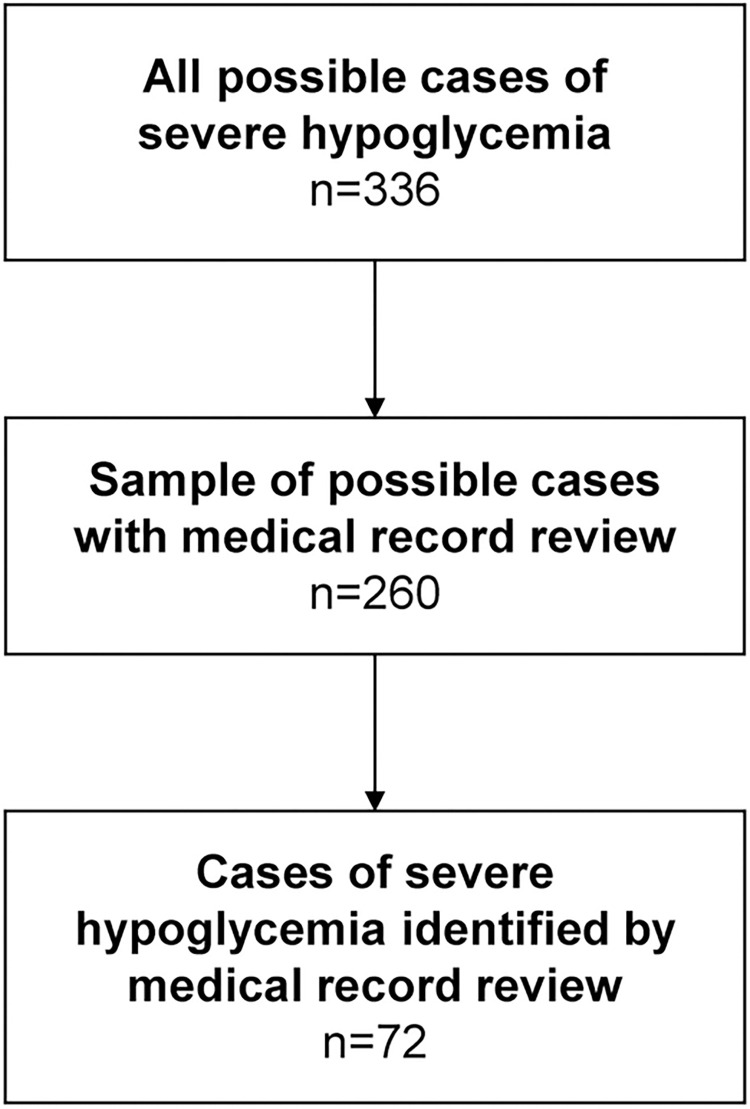
Case selection.

The representativeness of the selected medical institutions is shown in S5 Table, which compares the age distribution and inpatient/outpatient ratio of the patients in the 3 selected hospitals to those of potential patients in all medical institutions in the MDV database. The total number of possible cases of hypoglycemia identified by algorithm was 336, of which 260 were sampled for validation. Of these, 72 were identified as cases of severe hypoglycemia by medical record review (Fig 2). The Kappa statistic was 1, indicating 100% agreement between the 2 medical record reviewers at each hospital.

The analysis population comprised 124 (47.7%) inpatients and 136 (52.3%) outpatients (Table 1), of whom a greater proportion were male (n = 158; 60.8%) than female (n = 102; 39.2%). The median age was 73 years (IQR 63.0–82.0). At the time of the hospital visit, 37 (14.2%) had a prescription for sulfonylurea and 91 (35.0%) had a prescription for insulin. The median blood glucose level was 96.0 mg/dl (IQR 47.0–165.5; Table 1).

**Table 1:** Patient characteristics.

Characteristic	Analysis population
	N = 260
Sex, n (%)	
Male	158 (60.8)
Female	102 (39.2)
Age, years	
Mean (SD)	71.2 (14.3)
Median (IQR)	73.0 (63.0–82.0)
Age group, n (%)	
<65 years	70 (26.9)
≥65 years	190 (73.1)
Hospitalization category, n (%)	
Inpatient	124 (47.7)
Outpatient	136 (52.3)
Charlson Comorbidity Index	
Mean (SD)	2.9 (2.5)
Median (IQR)	2.0 (1.0–4.0)
Sulfonylurea treatment, n (%)	
No	223 (85.8)
Yes	37 (14.2)
Insulin treatment, n (%)	
No	169 (65.0)
Yes	91 (35.0)
Blood glucose, mg/dL	
Mean (SD)	133.8 (141.7)
Median (IQR)	96.0 (47.0–165.5)
Missing, n (%)	37 (14.2)

IQR = interquartile range; SD = standard deviation.

### Assessment of algorithms

S6 Table summarizes the performance metrics for all 61 case-identifying algorithms evaluated. The prevalence of cases judged by medical record review to be severe hypoglycemia was 0.277. No algorithm had both high PPV and high sensitivity for identifying severe hypoglycemia (S6 Table). Table 2 shows the 14 best-performing algorithms, categorized as “sensitivity-focused” (≥0.75 sensitivity), “PPV-focused” (≥0.75 PPV; ≥0.30 sensitivity), or “balanced-type” algorithms (≥0.50 for both sensitivity and PPV). Algorithms 1, 2, 13, 14, 55, and 56 were sensitivity-focused by this categorization scheme. Algorithm 1, which broadly encompassed any hypoglycemia diagnoses or prescription of high-concentration glucose, identified 336 cases at participating hospitals, representing all possible cases of severe hypoglycemia in this study, including suspected cases (sensitivity: 1.000; 95% CI 1.000, 1.000; PPV: 0.277; 95% CI 0.223, 0.331). Algorithm 2 was identical to Algorithm 1 but excluded suspected diagnoses (sensitivity: 0.986; 95% CI 0.959, 1.013; PPV: 0.345; 95% CI 0.280, 0.410). Restricting the variety of diagnoses included in Algorithm 1 and 2 by excluding non-diabetic and drug-induced hypoglycemia with coma and “other hypoglycemia” (Algorithms 13 and 14, respectively) or by including only “hypoglycemia, unspecified” (Algorithms 55 and 56, respectively) reduced the number of true-positive cases identified. This narrowing of search criteria only slightly reduced the sensitivity of case identification (Table 2).

**Table 2:** Sensitivity and PPV of case-identifying algorithms for severe hypoglycemia (selected algorithms).

Algorithm case definitions^a^	Cases with target condition^b^^,^^c^	Index test positive^d^	True positive^e^	PPV^f^^,^^g^ (95% CI)	Sensitivity^g^^,^^h^ (95% CI)
**Sensitivity-focused**					
1. Any diagnoses (including suspected diagnoses) listed in footnote (a) recorded or high concentration of glucose (i.e., ≥20%) prescribed at index date	72	260	72	0.277 (0.223, 0.331)	1.000 (1.000, 1.000)
2. Any diagnoses (excluding suspected diagnoses) listed in footnote (a) recorded or high concentration of glucose (i.e., ≥20%) prescribed at index date	72	206	71	0.345 (0.280, 0.410)	0.986 (0.959, 1.013)
13. Diagnosis information in algorithm 1 limited to E10.0, E11.0, E14.0, E16.2	72	229	69	0.301 (0.242, 0.361)	0.958 (0.912, 1.004)
14. Diagnosis information in algorithm 2 limited to E10.0, E11.0, E14.0, E16.2	72	198	68	0.343 (0.277, 0.410)	0.944 (0.892, 0.997)
55. Diagnosis information in algorithm 1 limited to E16.2	72	224	69	0.308 (0.248, 0.369)	0.958 (0.912, 1.004)
56. Diagnosis information in algorithm 2 limited to E16.2	72	193	68	0.352 (0.285, 0.420)	0.944 (0.892, 0.997)
**PPV-focused**					
5. Any diagnoses (including suspected diagnoses) listed in footnote (a) recorded and high concentration of glucose (i.e., ≥20%) prescribed at index date	72	36	28	0.778 (0.642, 0.914)	0.389 (0.276, 0.501)
6. Any diagnoses (excluding suspected diagnoses) listed in footnote (a) recorded and high concentration of glucose (i.e., ≥20%) prescribed at index date	72	35	27	0.771 (0.632, 0.911)	0.375 (0.263, 0.487)
17. Diagnosis information in algorithm 5 limited to E10.0, E11.0, E14.0, E16.2	72	33	25	0.758 (0.611, 0.904)	0.347 (0.237, 0.457)
18. Diagnosis information in algorithm 6 limited to E10.0, E11.0, E14.0, E16.2	72	32	24	0.750 (0.600, 0.900)	0.333 (0.224, 0.442)
59. Diagnosis information in algorithm 5 limited to E16.2	72	30	23	0.767 (0.615, 0.918)	0.319 (0.212, 0.427)
60. Diagnosis information in algorithm 6 limited to E16.2	72	29	22	0.759 (0.603, 0.914)	0.306 (0.199, 0.412)
**Balanced-type**					
4. Any diagnoses (excluding suspected diagnoses) listed in footnote (a) recorded at the index date	72	94	47	0.500 (0.399, 0.601)	0.653 (0.543, 0.763)
58. Diagnosis information in algorithm 4 limited to E16.2.	72	75	39	0.520 (0.407, 0.633)	0.542 (0.427, 0.657)

^a^ICD-10 codes [24] corresponding to names of diseases for possible hypoglycemia: E10.0 = type 1 diabetes mellitus with coma; E11.0 = type 2 diabetes mellitus with coma; E14.0 = unspecified diabetes mellitus with coma; E15 = non-diabetic hypoglycemic coma; E16.0 = drug-induced hypoglycemia without coma; E16.1 = other hypoglycemia; E16.2 = hypoglycemia, unspecified

^b^The total number of possible cases identified with the algorithm was 336, of which 260 were sampled for validation.

^c^Cases with the target condition were identified by medical record review as severe hypoglycemia and were the reference standard in this study. These cases may include both true positives and false negatives.

^d^Index test-positive cases met the criteria for each case-identifying algorithm and may include both true-positive cases and false-positive cases.

^e^True-positive cases met the criteria for each case-identifying algorithm and were identified as cases of severe hypoglycemia by medical record review.

^f^PPV was calculated based on the number of true-positive cases divided by the number of index test-positive cases (i.e., the sum of the number of true-positive and false-positive cases).

^g^95% CIs were calculated using normal approximation to the binomial distribution.

^h^Sensitivity was calculated by dividing the number of true-positive cases by the number of cases with the target condition (i.e., the sum of true-positive and false-negative cases) with the assumption that all cases with the target condition met the eligibility criteria and were included in the study population.

CI = confidence interval; ICD-10 = International Statistical Classification of Diseases and Related Health Problems, 10^th^ revision; PPV = positive predictive value.

Algorithms 5, 6, 17, 18, 59, and 60 were considered PPV-focused algorithms and included only cases that had both a diagnosis of hypoglycemia and prescription of high-concentration glucose, which improved the performance of the algorithms to correctly classify algorithm-positive cases as severe hypoglycemia but reduced the number of true-positive cases identified, thereby reducing sensitivity. Algorithm 5 had the highest PPV and sensitivity (sensitivity: 0.389; 95% CI 0.276, 0.501; PPV: 0.778; 95% CI 0.642, 0.914). Algorithm 6, which was identical to Algorithm 5 but excluded suspected diagnoses, had slightly lower sensitivity and PPV (sensitivity: 0.375; 95% CI 0.263, 0.487; PPV: 0.771; 95% CI 0.632, 0.911). Restricting the variety of diagnoses considered by Algorithm 5 and 6 further (i.e., Algorithms 17, 18, 59, and 60) slightly reduced the number of true-positive cases identified and did not improve the PPV of the algorithms (Table 2).

Algorithms 4 and 58 were considered balanced-type algorithms. The best performing balanced-type algorithm was Algorithm 4, which included any diagnosis of hypoglycemia except suspected diagnoses, but did not include drug prescription as a second condition of identification. This substantially reduced both the PPV and sensitivity of the algorithm (PPV: 0.500; 95% CI 0.399, 0.601; sensitivity: 0.653; 95% CI 0.543, 0.763) compared to the best-performing PPV- and sensitivity-focused algorithms (Table 2). Algorithm 58 restricted diagnoses considered by Algorithm 4 to only E16.2-coded diagnoses (“hypoglycemia, unspecified”), which decreased the number of true-positive cases identified by 8 cases, reducing sensitivity (0.542; 95% CI 0.427, 0.657) but not PPV (0.520; 95% CI 0.407, 0.633).

The sensitivity and PPV of 14 selected, best-performing algorithms (sensitivity-focused, PPV-focused, and balanced-type) are shown by subgroup in S7 Table. For the subgroup analyses, the number of cases sampled for validation included 158 males and 102 females in the sex subgroup, 70 patients <65 years and 190 patients ≥65 years in the age subgroup, and 124 inpatients and 136 outpatients in the hospitalization subgroup. No major differences were observed between the sensitivity or PPV of selected algorithms in any of the subgroups compared to those in the overall population. In an informal comparison of the data within the hospitalization subgroup, PPVs for the inpatient group trended approximately 10% higher across the PPV-focused algorithms compared to PPVs for the outpatient group (S7 Table).

## Discussion

This study evaluated the performance of 61 case-identifying algorithms for the detection of severe hypoglycemic events requiring inpatient or outpatient hospital visits. None of the algorithms had both high PPV and high sensitivity for the detection of cases judged to have the target condition by medical record review. Six algorithms were identified that had sensitivity ≥0.75, and 6 algorithms were identified that had a PPV ≥0.75 with sensitivity ≥0.30. Two algorithms with moderate PPV and sensitivity (≥0.50 each) were also identified. Algorithms 1 and 2 (any hypoglycemia diagnosis or prescription of high-concentration glucose, including and excluding suspected cases, respectively) were the best performing sensitivity-focused algorithms whereas Algorithms 5 and 6 (any hypoglycemia diagnosis plus prescription of high-concentration glucose, including and excluding suspected cases, respectively) were considered the best performing PPV-focused algorithms. In the Japanese health claims system, a suspected disease flag is generally included to support claims for diagnostic tests, and the flag is removed if a diagnosis of exclusion for the disease is confirmed. Therefore, Algorithms 2 and 6 may be preferrable for most applications, as these excluded suspected cases.

Prior studies on the validation of case-finding algorithms for hypoglycemia using electronic healthcare databases reported PPVs ranging from 48% to 99.2% [14–18]. Sensitivity was also reported by Hodge et al., 2017 [15], who found their algorithm had a high PPV at 94% but low sensitivity (12.7%). In contrast, in their validation study using United States Medicare claims data, Yang et al., 2022, reported moderate PPV (69.2%) and moderate sensitivity (83.9%) across all acute-care encounters, with better accuracy observed for emergency department visits (PPV: 92.9%; sensitivity: 100%) than inpatient visits (PPV: 53.7%; sensitivity: 71.0%) [18]. These data suggest that the current best-performing algorithms, Algorithms 2 and 6, may offer improved sensitivity or PPV over some of the previously proposed algorithms. However, there are a number of study design and methodological differences that must be considered when making cross-study comparisons of algorithm performance, including, for example, the use of different administrative codes and/or different versions of the ICD to define hypoglycemia [14, 16, 17] and different study cohort inclusion criteria (e.g., Hodge et al., 2017 [15], only assessed patients >65 years of age). In addition, ethnic-, country-, and site-specific factors should also be considered [19–23]. Additional studies are needed to confirm the performance of the currently described algorithms in other contexts. Our inclusion of comprehensive assessment metrics for all 61 algorithms examined in the current study should facilitate future comparisons and the refinement of algorithms.

It is well recognized that different types of case-identifying algorithms may be best suited to different applications and, when developing algorithms, certain performance metrics may need to be prioritized over others, depending on the application [30]. An algorithm with high PPV would be expected to better classify algorithm-positive cases as severe hypoglycemia whereas an algorithm with high sensitivity would be expected to identify most severe hypoglycemia cases in a patient population [30]. High-sensitivity algorithms that broadly define severe hypoglycemia may be the most appropriate for assessment of drug safety signals, as the use of broad definitions would maximize the number of cases of severe hypoglycemia identified in a patient population [10]. There are frequently trade-offs between the accuracy metrics [30–32]. As PPV represents the likelihood that an algorithm correctly identifies a case as severe hypoglycemia, prioritizing high PPV would increase the number of true-positive cases of severe hypoglycemia identified, which would be expected to reduce bias in the relative risk estimates [30]. As the algorithms with high sensitivity (≥0.75) in this study did not also have high PPV (≥0.75), more false-positive cases would be identified by these algorithms. Conversely, as the algorithms with high PPV defined in this study did not also have high sensitivity, some target cases may be missed when using these algorithms. When choosing among the best-performing algorithms described in this study for future studies, prioritization of sensitivity versus PPV should be assessed in terms of the intended application and setting. The balanced-type algorithms identified in this study might be of limited use for future studies using hospital administrative data in Japan, given their lower PPV and sensitivity than the best performing sensitivity- and PPV-focused algorithms.

Overall, no major differences were observed between the performance of selected algorithms in any of the subgroups compared to the overall population. In the hospitalization subgroup, PPV for the inpatient group was approximately 10% higher than that for the outpatient group. Consistent with this finding, Min et al. (2019) [17] also reported a higher PPV for “hospitalization events” than for outpatient “emergency department events” (80% and 48%, respectively). Nevertheless, the PPV for the outpatient subgroup in the current study was approximately 70% for the PPV-focused algorithms, suggesting that our algorithms may be well suited for case identification in outpatient populations.

Strengths of this study include validation of algorithms based on the findings of 2 physicians at each of 3 sites who independently reviewed medical records. In addition, the age distribution and ratio of inpatients to outpatients at the 3 institutions were similar to those in the overall MDV database, indicating that the selected medical institutions were representative of the institutions covered in the MDV database in terms of these characteristics. The patients included in the current validation study also appear representative of severe hypoglycemia cases reported more broadly within the diabetic population in Japan, based on a comparison of the patient characteristics (i.e., the ratio of males to females, ratio of inpatients to outpatients, and age ranges) in this study and those of patients reporting severe hypoglycemia in a national Japan Diabetes Society survey conducted at 193 medical facilities [33].

This study had several limitations. Notably, since the MDV database covers Japanese DPC hospitals only, the applicability of the algorithms evaluated in this study to the other settings is unknown. Performance of the algorithms in other contexts may differ and particular consideration should be given to differences in coding practices and in the clinical characteristics of the study cohort represented in different electronic databases [30]. We also calculated sensitivity under the assumption that all cases with the target condition met the eligibility criteria and were included in the study, which may not have captured all cases in the hospital databases. In addition, fewer true-positive cases were found in the <65 years category compared to the ≥65 years category. However, this finding is not unexpected given that the highest rates of severe hypoglycemia have previously been reported among elderly patients and those with multiple comorbidities [34]. Finally, negative predictive value and specificity were not calculated, as both statistics pertain to patients without possible hypoglycemia, which were not included in this study, thus rendering these statistics inapplicable to other settings which include patients both with and without possible hypoglycemia.

## Conclusions

This study evaluated several algorithms designed to detect cases of severe hypoglycemia in Japanese hospital administrative databases that contained claims and DPC data. Our analysis identified the best performing algorithms as those that excluded suspected cases and required either a diagnosis for possible hypoglycemia or a prescription of high-concentration glucose (Algorithm 2) or both a diagnosis of possible hypoglycemia and a prescription of high-concentration glucose (Algorithm 6). It is anticipated that the high-performing case-identifying algorithms in this study will be of utility in future studies that evaluate severe hypoglycemia in Japan and utilize hospital administrative data with consideration of the suitability of the type of algorithm (e.g., high sensitivity versus high PPV), depending on the purpose and context of application. Additional studies are warranted to confirm the current findings in and other Japanese healthcare database systems.

## Supporting information

S1 Fig Calculations for performance metrics. Cases of severe hypoglycemia identified by the algorithm were termed ‘index test-positive’ cases. Independent manual chart reviews by 2 physicians at each participating hospital were used as the reference standard. Cases were considered: (a) “true positives” if identified as severe hypoglycemia by both the algorithm and medical record review; (b) “false negatives” if identified by medical record review but not by the algorithm; (c) “false positives” if identified by the algorithm but not by medical record review; and (d) “true negatives” if identified as not cases of severe hypoglycemia by both the algorithm and medical record review. Algorithm validity was evaluated using standard performance metrics. PPV represents the likelihood that the algorithm correctly identifies a case as severe hypoglycemia. Sensitivity measures the true positive ratio (calculated herein under the assumption that all cases with the target condition met the eligibility criteria and were included in the study). Prevalence was calculated by dividing the number of cases identified as severe hypoglycemia by medical record review by the total number of cases. Probability+ was calculated by dividing the number of index test-positive cases by the total number of cases. Probability- was calculated by dividing the number of index-case negative cases by the total number of cases. NPV (the likelihood that the algorithm correctly classifies a case as not severe hypoglycemia) and specificity (the true negative ratio when comparing the performance of the algorithm to the reference standard) are not included herein as these statistics pertain to patients who do not have severe hypoglycemia. NPV and specificity derived from the current study, which did not include patients without possible hypoglycemia, would not be applicable to other settings that include patients with and without possible hypoglycemia. NPV = negative predictive value; PPV = positive predictive value.
(TIF)

S1 Table Judgement sheet for severe hypoglycemia. (DOCX)

S2 Table Overview of case-identification algorithms for severe hypoglycemia. (DOCX)

S3 Table List of diagnostic codes. (DOCX)

S4 Table List of drug codes. (DOCX)

S5 Table Analysis for hospital selection. (DOCX)

S6 Table Performance metrics of case-identifying algorithms for severe hypoglycemia (all evaluated algorithms). (DOCX)

S7 Table Performance metrics of selected algorithms for severe hypoglycemia in patient subgroups (selected algorithms). (DOCX)

S1 Appendix Minimal data set. (XLSX)
