# Supplementary figures and images for: Validation study of case-identifying algorithms for severe hypoglycemia using hospital administrative data in Japan
Source: PLoS One. 2023 Aug 9;18(8):e0289840. doi: 10.1371/journal.pone.0289840 (PMC10411751; doi:10.1371/journal.pone.0289840)

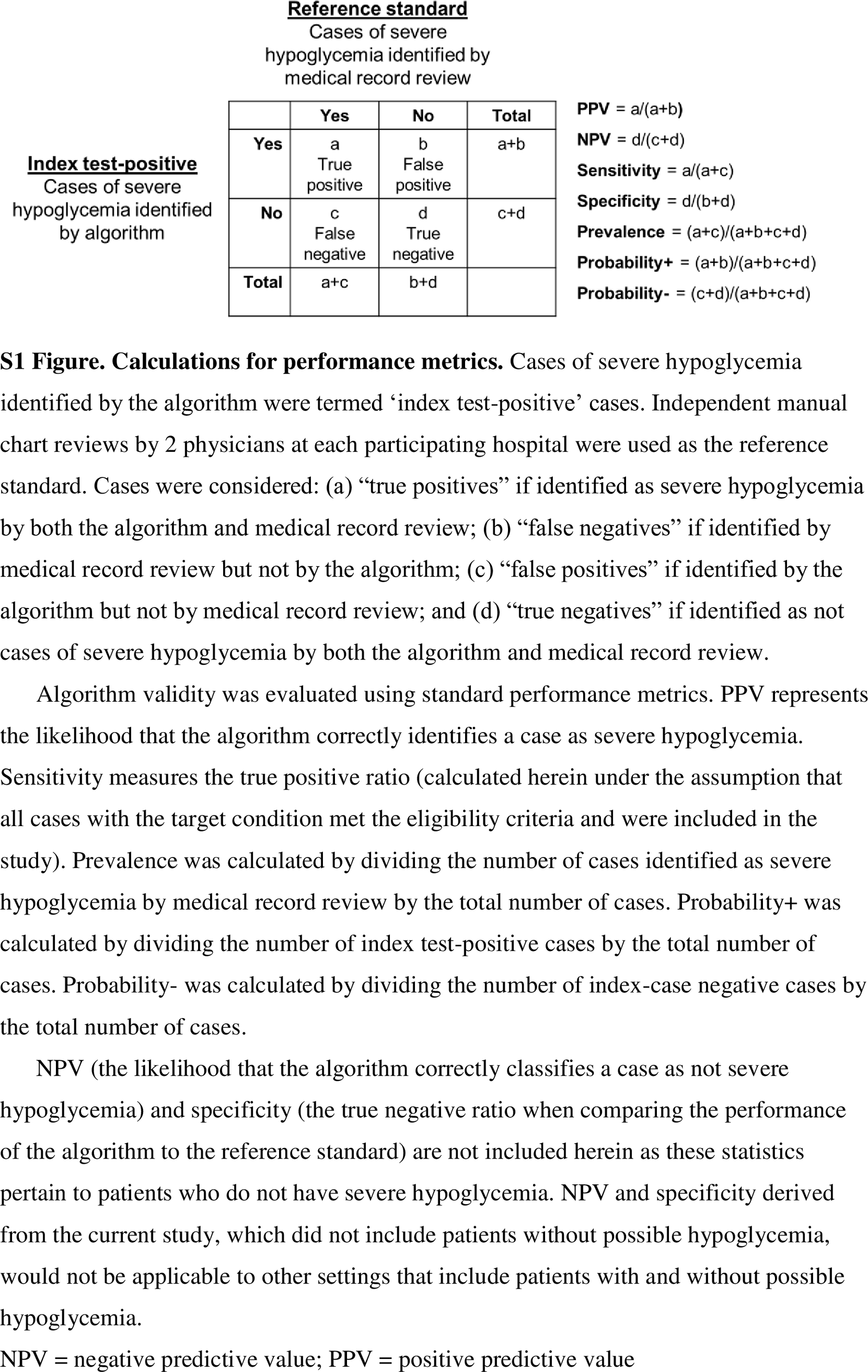

Supplement: S1 Fig — Cases of severe hypoglycemia identified by the algorithm were termed ‘index test-positive’ cases. Independent manual chart reviews by 2 physicians at each participating hospital were used as the reference standard. Cases were considered: (a) “true positives” if identified as severe hypoglycemia by both the algorithm and medical record review; (b) “false negatives” if identified by medical record review but not by the algorithm; (c) “false positives” if identified by the algorithm but not by medical record review; and (d) “true negatives” if identified as not cases of severe hypoglycemia by both the algorithm and medical record review. Algorithm validity was evaluated using standard performance metrics. PPV represents the likelihood that the algorithm correctly identifies a case as severe hypoglycemia. Sensitivity measures the true positive ratio (calculated herein under the assumption that all cases with the target condition met the eligibility criteria and were included in the study). Prevalence was calculated by dividing the number of cases identified as severe hypoglycemia by medical record review by the total number of cases. Probability+ was calculated by dividing the number of index test-positive cases by the total number of cases. Probability- was calculated by dividing the number of index-case negative cases by the total number of cases. NPV (the likelihood that the algorithm correctly classifies a case as not severe hypoglycemia) and specificity (the true negative ratio when comparing the performance of the algorithm to the reference standard) are not included herein as these statistics pertain to patients who do not have severe hypoglycemia. NPV and specificity derived from the current study, which did not include patients without possible hypoglycemia, would not be applicable to other settings that include patients with and without possible hypoglycemia. NPV = negative predictive value; PPV = positive predictive value. (TIF) [file pone.0289840.s001.tif]
