# Supplementary material for: Validation study of case-identifying algorithms for severe hypoglycemia using hospital administrative data in Japan
Source: PLoS One. 2023 Aug 9;18(8):e0289840. doi: 10.1371/journal.pone.0289840 (PMC10411751; doi:10.1371/journal.pone.0289840)
Supplement: S1 Table — (DOCX) [file pone.0289840.s002.docx]

**S1 Table. Judgement sheet for severe hypoglycemia**

| **Item** | **Raters’ judgement** | **Support for judgement written in medical records** |
| --- | --- | --- |
| Description of sympathetic symptoms (finger tremor, palpitation, cold sweat, etc.) that recovery could not be achieved without assistance/treatment of others* until the time of hospital visit or until recovery after visit | Yes/No | [*this column is for medical rater annotations*] |
| Description of central nervous system symptoms (decreased level of consciousness, abnormal behavior, convulsion, coma, etc.) that recovery could not be achieved without assistance/treatment of others* until the time of hospital visit or until recovery after visit | Yes/No |  |
| Description of interventions for recovery by others* (intake of drinking water containing glucose, injection of glucagon, intravenous injection of 20% or more glucose, etc.) | Yes/No |  |
| Judgement of severe hypoglycemia (when there are ‘1 and 3’ or ‘2 and 3’, it is judged as a true case) | True case/false case | -- |

*Including family members and medical institutions
