# Supplementary material for: Validation study of case-identifying algorithms for severe hypoglycemia using hospital administrative data in Japan
Source: PLoS One. 2023 Aug 9;18(8):e0289840. doi: 10.1371/journal.pone.0289840 (PMC10411751; doi:10.1371/journal.pone.0289840)
Supplement: S2 Table — (DOCX) [file pone.0289840.s003.docx]

**S2 Table. Overview of case-identification algorithms for severe hypoglycemia**

| **Algorithm** | **Definition for Index test positive** |
| --- | --- |
| **1** | Any diagnoses (including suspected diagnoses) listed in footnote (a) are recorded or high concentration glucose (i.e., 20% or more) is prescribed at the index date. |
| **2** | Any diagnoses (excluding suspected diagnoses) listed in footnote (a) are recorded or high concentration glucose (i.e., 20% or more) is prescribed at the index date. |
| **3** | Any diagnoses (including suspected diagnoses) listed in footnote (a) are recorded at the index date. |
| **4** | Any diagnoses (excluding suspected diagnoses) listed in footnote (a) are recorded at the index date. |
| **5** | Any diagnoses (including suspected diagnoses) listed in footnote (a) are recorded and high concentration glucose (i.e., 20% or more) is prescribed at the index date. |
| **6** | Any diagnoses (excluding suspected diagnoses) listed in footnote (a) are recorded and high concentration glucose (i.e., 20% or more) is prescribed at the index date. |
| **7-12** | Diagnosis information in algorithms 1 to 6 is limited to one of E10.0, E11.0, E14.0. |
| **13-18** | Diagnosis information in algorithms 1 to 6 is limited to one of E10.0, E11.0, E14.0, E16.2. |
| **19-24** | Diagnosis information in algorithms 1 to 6 is limited to E10.0. |
| **25-30** | Diagnosis information in algorithms 1 to 6 is limited to E11.0. |
| **31-36** | Diagnosis information in algorithms 1 to 6 is limited to E14.0. |
| **37-42** | Diagnosis information in algorithms 1 to 6 is limited to E15. |
| **43-48** | Diagnosis information in algorithms 1 to 6 is limited to E16.0. |
| **49-54** | Diagnosis information in algorithms 1 to 6 is limited to E16.1. |
| **55-60** | Diagnosis information in algorithms 1 to 6 is limited to E16.2. |
| **61** | High-concentration glucose (i.e., 20% or more) is prescribed at the index date. |

^a^ICD-10 codes [24] corresponding to names of diseases for possible hypoglycemia: E10.0 = type 1 diabetes mellitus with coma; E11.0 = type 2 diabetes mellitus with coma; E14.0 = unspecified diabetes mellitus with coma; E15 = non-diabetic hypoglycemic coma; E16.0 = drug-induced hypoglycemia without coma; E16.1 = other hypoglycemia; E16.2 = hypoglycemia, unspecified
