# Supplementary material for: Validation study of case-identifying algorithms for severe hypoglycemia using hospital administrative data in Japan
Source: PLoS One. 2023 Aug 9;18(8):e0289840. doi: 10.1371/journal.pone.0289840 (PMC10411751; doi:10.1371/journal.pone.0289840)
Supplement: S3 Table — (DOCX) [file pone.0289840.s004.docx]

**S3 Table. List of diagnostic codes**

| **Category** | **ICD10** | **Diagnosis Code** | **Diagnosis Name** |
| --- | --- | --- | --- |
| Diabetes mellitus | E10 | 2500014 | Type 1 diabetes mellitus |
| Diabetes mellitus | E10 | 2500027 | Diabetes brittle (unstable diabetes) |
| Diabetes mellitus | E10 | 8844022 | Slowly progressive type 1 diabetes mellitus |
| Diabetes mellitus | E100 | 8830030 | Type 1 diabetic coma |
| Diabetes mellitus | E100 | 8841679 | Type 1 diabetes mellitus with coma |
| Diabetes mellitus | E100 | 8844026 | Slowly progressive type 1 diabetes mellitus with coma |
| Diabetes mellitus | E100 | 8845065 | Type 1 diabetic hypoglycemic coma |
| Diabetes mellitus | E101 | 8830028 | Type 1 diabetic ketoacidosis |
| Diabetes mellitus | E101 | 8841680 | Type 1 diabetes mellitus with ketoacidosis |
| Diabetes mellitus | E101 | 8844025 | Slowly progressive type 1 diabetes mellitus with ketoacidosis |
| Diabetes mellitus | E101 | 8844045 | Fulminant type 1 diabetes mellitus |
| Diabetes mellitus | E101 | 8845044 | Type 1 diabetic acidosis |
| Diabetes mellitus | E101 | 8845045 | Type 1 diabetic acetonemia |
| Diabetes mellitus | E101 | 8849056 | Type 1 diabetic ketosis |
| Diabetes mellitus | E102 | 8830031 | Type 1 diabetic nephropathy |
| Diabetes mellitus | E102 | 8841681 | Type 1 diabetes mellitus with renal complications |
| Diabetes mellitus | E102 | 8843983 | Type 1 diabetic nephropathy stage 1 |
| Diabetes mellitus | E102 | 8843984 | Type 1 diabetic nephropathy stage 2 |
| Diabetes mellitus | E102 | 8843985 | Type 1 diabetic nephropathy stage 3 |
| Diabetes mellitus | E102 | 8843986 | Type 1 diabetic nephropathy stage 3A |
| Diabetes mellitus | E102 | 8843987 | Type 1 diabetic nephropathy stage 3B |
| Diabetes mellitus | E102 | 8843988 | Type 1 diabetic nephropathy stage 4 |
| Diabetes mellitus | E102 | 8843989 | Type 1 diabetic nephropathy stage 5 |
| Diabetes mellitus | E102 | 8844028 | Slowly progressive type 1 diabetes mellitus with renal complications |
| Diabetes mellitus | E102 | 8845058 | Type 1 diabetic renal sclerosis |
| Diabetes mellitus | E102 | 8845059 | Type 1 diabetic kidney failure |
| Diabetes mellitus | E103 | 8830033 | Type 1 diabetic retinopathy |
| Diabetes mellitus | E103 | 8841682 | Type 1 diabetes mellitus with ophthalmic complications |
| Diabetes mellitus | E103 | 8843982 | Type 1 diabetic macular oedema |
| Diabetes mellitus | E103 | 8844024 | Slowly progressive type 1 diabetes mellitus with ophthalmic complications |
| Diabetes mellitus | E103 | 8844346 | Type 1 diabetic cataract |
| Diabetes mellitus | E103 | 8844536 | Proliferative type 1 diabetic retinopathy |
| Diabetes mellitus | E103 | 8845043 | Type 1 diabetic maculopathy |
| Diabetes mellitus | E103 | 8845049 | Type 1 diabetic ophthalmoplegia |
| Diabetes mellitus | E103 | 8845053 | Type 1 diabetic iritis |
| Diabetes mellitus | E103 | 8845064 | Type 1 diabetic central retinopathy |
| Diabetes mellitus | E104 | 8830032 | Type 1 diabetic neuropathy |

| **Category** | **ICD10** | **Diagnosis Code** | **Diagnosis Name** |
| --- | --- | --- | --- |
| Diabetes mellitus | E104 | 8841683 | Type 1 diabetes mellitus with neurological complications |
| Diabetes mellitus | E104 | 8844027 | Slowly progressive type 1 diabetes mellitus with neurological complications |
| Diabetes mellitus | E104 | 8845050 | Type 1 diabetic amyotrophy |
| Diabetes mellitus | E104 | 8845055 | Type 1 diabetic neuropathic bladder |
| Diabetes mellitus | E104 | 8845056 | Type 1 diabetic neuralgia |
| Diabetes mellitus | E104 | 8845057 | Type 1 diabetic autonomic neuropathy |
| Diabetes mellitus | E104 | 8845062 | Type 1 diabetic polyneuropathy |
| Diabetes mellitus | E104 | 8845063 | Type 1 diabetic mononeuropathy |
| Diabetes mellitus | E104 | 8845071 | Type 1 diabetic peripheral neuropathy |
| Diabetes mellitus | E105 | 8841684 | Type 1 diabetes mellitus with peripheral circulatory complications |
| Diabetes mellitus | E105 | 8843105 | Type 1 diabetic gangrene |
| Diabetes mellitus | E105 | 8844031 | Slowly progressive type 1 diabetes mellitus with peripheral circulatory complications |
| Diabetes mellitus | E105 | 8845046 | Type 1 diabetic ulcer |
| Diabetes mellitus | E105 | 8845051 | Type 1 diabetic vascular disorders |
| Diabetes mellitus | E105 | 8845066 | Type 1 diabetic arteriosclerosis |
| Diabetes mellitus | E105 | 8845067 | Type 1 diabetic arterial occlusion |
| Diabetes mellitus | E105 | 8845069 | Type 1 diabetic peripheral angiopathy |
| Diabetes mellitus | E105 | 8845070 | Type 1 diabetic peripheral vascular disease |
| Diabetes mellitus | E106 | 8841685 | Type 1 diabetes mellitus with joint complications |
| Diabetes mellitus | E106 | 8841686 | Type 1 diabetes mellitus with complications |
| Diabetes mellitus | E106 | 8844023 | Slowly progressive type 1 diabetes mellitus with joint complications |
| Diabetes mellitus | E106 | 8844626 | Type 1 diabetic blister |
| Diabetes mellitus | E106 | 8844627 | Type 1 scleroedema diabeticorum |
| Diabetes mellitus | E106 | 8845047 | Type 1 diabetic hepatopathy |
| Diabetes mellitus | E106 | 8845048 | Type 1 diabetic arthrosis |
| Diabetes mellitus | E106 | 8845052 | Type 1 diabetic hypercholesteremia |
| Diabetes mellitus | E106 | 8845054 | Type 1 diabetic osteosis |
| Diabetes mellitus | E106 | 8845060 | Type 1 diabetic psychic disturbance |
| Diabetes mellitus | E106 | 8845061 | Type 1 diabetic pruritus |
| Diabetes mellitus | E106 | 8845068 | Type 1 diabetic dermopathy |
| Diabetes mellitus | E106 | 8845842 | Type 1 diabetic gastroenteropathy |
| Diabetes mellitus | E106 | 8849557 | Type 1 diabetic hyperglycemic hyperosmolar syndrome |
| Diabetes mellitus | E107 | 8841687 | Type 1 diabetes mellitus with multiple complications |
| Diabetes mellitus | E107 | 8844029 | Slowly progressive type 1 diabetes mellitus with multiple complications |
| Diabetes mellitus | E109 | 8841688 | Type 1 diabetes mellitus without complications |
| Diabetes mellitus | E109 | 8844030 | Slowly progressive type 1 diabetes mellitus without complications |
| Diabetes mellitus | E11 | 2500001 | Insulin-resistant diabetes |
| Diabetes mellitus | E11 | 2500015 | Type 2 diabetes mellitus |
| Diabetes mellitus | E11 | 8830405 | Stable diabetes mellitus |
| Diabetes mellitus | E11 | 8835244 | Juvenile type 2 diabetes mellitus |

| **Category** | **ICD10** | **Diagnosis Code** | **Diagnosis Name** |
| --- | --- | --- | --- |
| Diabetes mellitus | E110 | 8830041 | Type 2 diabetic coma |
| Diabetes mellitus | E110 | 8841689 | Type 2 diabetes mellitus with coma |
| Diabetes mellitus | E110 | 8845094 | Type 2 diabetic hypoglycemic coma |
| Diabetes mellitus | E111 | 8830040 | Type 2 diabetic ketoacidosis |
| Diabetes mellitus | E111 | 8841690 | Type 2 diabetes mellitus with ketoacidosis |
| Diabetes mellitus | E111 | 8845073 | Type 2 diabetic acidosis |
| Diabetes mellitus | E111 | 8845074 | Type 2 diabetic acetonemia |
| Diabetes mellitus | E111 | 8849058 | Type 2 diabetic ketosis |
| Diabetes mellitus | E112 | 8830042 | Type 2 diabetic nephropathy |
| Diabetes mellitus | E112 | 8841691 | Type 2 diabetes mellitus with renal complications |
| Diabetes mellitus | E112 | 8843991 | Type 2 diabetic nephropathy stage 1 |
| Diabetes mellitus | E112 | 8843992 | Type 2 diabetic nephropathy stage 2 |
| Diabetes mellitus | E112 | 8843993 | Type 2 diabetic nephropathy stage 3 |
| Diabetes mellitus | E112 | 8843994 | Type 2 diabetic nephropathy stage 3A |
| Diabetes mellitus | E112 | 8843995 | Type 2 diabetic nephropathy stage 3B |
| Diabetes mellitus | E112 | 8843996 | Type 2 diabetic nephropathy stage 4 |
| Diabetes mellitus | E112 | 8843997 | Type 2 diabetic nephropathy stage 5 |
| Diabetes mellitus | E112 | 8845087 | Type 2 diabetic renal sclerosis |
| Diabetes mellitus | E112 | 8845088 | Type 2 diabetic kidney failure |
| Diabetes mellitus | E113 | 8830045 | Type 2 diabetic retinopathy |
| Diabetes mellitus | E113 | 8841692 | Type 2 diabetes mellitus with ophthalmic complications |
| Diabetes mellitus | E113 | 8843990 | Type 2 diabetic macular edema |
| Diabetes mellitus | E113 | 8844347 | Type 2 diabetic cataract |
| Diabetes mellitus | E113 | 8844537 | Proliferative type 2 diabetic retinopathy |
| Diabetes mellitus | E113 | 8845072 | Type 2 diabetic maculopathy |
| Diabetes mellitus | E113 | 8845078 | Type 2 diabetic ophthalmoplegia |
| Diabetes mellitus | E113 | 8845082 | Type 2 diabetic iritis |
| Diabetes mellitus | E113 | 8845093 | Type 2 diabetic central retinopathy |
| Diabetes mellitus | E114 | 8830043 | Type 2 diabetic neuropathy |
| Diabetes mellitus | E114 | 8830044 | Type 2 diabetic myopathies |
| Diabetes mellitus | E114 | 8841693 | Type 2 diabetes mellitus with neurological complications |
| Diabetes mellitus | E114 | 8845079 | Type 2 diabetic amyotrophy |
| Diabetes mellitus | E114 | 8845084 | Type 2 diabetic neuropathic bladder |
| Diabetes mellitus | E114 | 8845085 | Type 2 diabetic neuralgia |
| Diabetes mellitus | E114 | 8845086 | Type 2 diabetic autonomic neuropathy |
| Diabetes mellitus | E114 | 8845091 | Type 2 diabetic polyneuropathy |
| Diabetes mellitus | E114 | 8845092 | Type 2 diabetic mononeuropathy |
| Diabetes mellitus | E114 | 8845100 | Type 2 diabetic peripheral neuropathy |
| Diabetes mellitus | E115 | 8841694 | Type 2 diabetes mellitus with peripheral circulatory complications |
| Diabetes mellitus | E115 | 8843106 | Type 2 diabetic gangrene |
| Diabetes mellitus | E115 | 8845075 | Type 2 diabetic ulcer |
| Diabetes mellitus | E115 | 8845080 | Type 2 diabetic vascular disorders |
| Diabetes mellitus | E115 | 8845095 | Type 2 diabetic arteriosclerosis |
| Diabetes mellitus | E115 | 8845096 | Type 2 diabetic arterial occlusion |
| Diabetes mellitus | E115 | 8845098 | Type 2 diabetic peripheral angiopathy |

| **Category** | **ICD10** | **Diagnosis Code** | **Diagnosis Name** |
| --- | --- | --- | --- |
| Diabetes mellitus | E115 | 8845099 | Type 2 diabetic peripheral vascular disease |
| Diabetes mellitus | E116 | 8841695 | Type 2 diabetes mellitus with joint complications |
| Diabetes mellitus | E116 | 8841696 | Type 2 diabetes mellitus with complications |
| Diabetes mellitus | E116 | 8844628 | Type 2 diabetic blister |
| Diabetes mellitus | E116 | 8844629 | Type 2 scleroedema diabeticorum |
| Diabetes mellitus | E116 | 8845076 | Type 2 diabetic hepatopathy |
| Diabetes mellitus | E116 | 8845077 | Type 2 diabetic arthrosis |
| Diabetes mellitus | E116 | 8845081 | Type 2 diabetic hypercholesteremia |
| Diabetes mellitus | E116 | 8845083 | Type 2 diabetic osteosis |
| Diabetes mellitus | E116 | 8845089 | Type 2 diabetic psychiatric disorder |
| Diabetes mellitus | E116 | 8845090 | Type 2 diabetic pruritus |
| Diabetes mellitus | E116 | 8845097 | Type 2 diabetic dermopathy |
| Diabetes mellitus | E116 | 8848108 | Type 2 diabetic gastroenteropathy |
| Diabetes mellitus | E116 | 8849558 | Type 2 diabetic hyperglycemic hyperosmolar syndrome |
| Diabetes mellitus | E117 | 8841697 | Type 2 diabetes mellitus with multiple complications |
| Diabetes mellitus | E119 | 8841698 | Type 2 diabetes mellitus without complications |
| Diabetes mellitus | E12 | 2500037 | Malnutrition-related diabetes mellitus |
| Diabetes mellitus | E13 | 2500024 | Pancreatogenous diabetes |
| Diabetes mellitus | E13 | 2500041 | Insulin receptor disorders |
| Diabetes mellitus | E13 | 2509003 | Steroid diabetes |
| Diabetes mellitus | E13 | 2509004 | Secondary diabetes |
| Diabetes mellitus | E13 | 8830756 | Viral diabetes mellitus |
| Diabetes mellitus | E13 | 8840710 | Drug-induced diabetes |
| Diabetes mellitus | E13 | 8845198 | Type B insulin receptor disorder |
| Diabetes mellitus | E13 | 8849585 | Hepatic diabetes mellitus |
| Diabetes mellitus | E13 | 8849874 | Insulin receptor disorders |
| Diabetes mellitus | E130 | 8843122 | Viral diabetes mellitus with coma |
| Diabetes mellitus | E130 | 8843377 | Pancreatogenous diabetes with coma |
| Diabetes mellitus | E130 | 8843390 | Steroid diabetes with coma |
| Diabetes mellitus | E130 | 8843450 | Secondary diabetes with coma |
| Diabetes mellitus | E130 | 8843621 | Drug-induced diabetes with coma |
| Diabetes mellitus | E130 | 8849588 | Hepatic diabetes mellitus with coma |
| Diabetes mellitus | E131 | 8843121 | Viral diabetes mellitus with ketoacidosis |
| Diabetes mellitus | E131 | 8843376 | Pancreatogenous diabetes with ketoacidosis |
| Diabetes mellitus | E131 | 8843389 | Steroid diabetes with ketoacidosis |
| Diabetes mellitus | E131 | 8843449 | Secondary diabetes with ketoacidosis |
| Diabetes mellitus | E131 | 8843620 | Drug-induced diabetes with ketoacidosis |
| Diabetes mellitus | E131 | 8849587 | Hepatic diabetes mellitus with ketoacidosis |
| Diabetes mellitus | E132 | 8843124 | Viral diabetes mellitus with renal complications |
| Diabetes mellitus | E132 | 8843379 | Pancreatogenous diabetes with renal complications |
| Diabetes mellitus | E132 | 8843392 | Steroid diabetes with renal complications |
| Diabetes mellitus | E132 | 8843452 | Secondary diabetes with renal complications |
| Diabetes mellitus | E132 | 8843623 | Drug-induced diabetes with renal complications |
| Diabetes mellitus | E132 | 8849590 | Hepatic diabetes mellitus with renal complications |

| **Category** | **ICD10** | **Diagnosis Code** | **Diagnosis Name** |
| --- | --- | --- | --- |
| Diabetes mellitus | E133 | 8843120 | Viral diabetes mellitus with ophthalmic complications |
| Diabetes mellitus | E133 | 8843375 | Pancreatogenous diabetes with ophthalmic complications |
| Diabetes mellitus | E133 | 8843388 | Steroid diabetes with ophthalmic complications |
| Diabetes mellitus | E133 | 8843448 | Secondary diabetes with ophthalmic complications |
| Diabetes mellitus | E133 | 8843619 | Drug-induced diabetes with ophthalmic complications |
| Diabetes mellitus | E133 | 8849586 | Hepatic diabetes mellitus with ophthalmic complications |
| Diabetes mellitus | E134 | 8843123 | Viral diabetes mellitus with neurological complications |
| Diabetes mellitus | E134 | 8843378 | Pancreatogenous diabetes with neurological complications |
| Diabetes mellitus | E134 | 8843391 | Steroid diabetes with neurological complications |
| Diabetes mellitus | E134 | 8843451 | Secondary diabetes with neurological complications |
| Diabetes mellitus | E134 | 8843622 | Drug-induced diabetes with neurological complications |
| Diabetes mellitus | E134 | 8849589 | Hepatic diabetes mellitus with neurological complications |
| Diabetes mellitus | E135 | 8843128 | Viral diabetes mellitus with peripheral circulatory complications |
| Diabetes mellitus | E135 | 8843383 | Pancreatogenous diabetes with peripheral circulatory complications |
| Diabetes mellitus | E135 | 8843396 | Steroid diabetes with peripheral circulatory  complications |
| Diabetes mellitus | E135 | 8843456 | Secondary diabetes with peripheral circulatory complications |
| Diabetes mellitus | E135 | 8843627 | Drug-induced diabetes with peripheral circulatory complications |
| Diabetes mellitus | E135 | 8849594 | Hepatic diabetes mellitus with peripheral circulatory complications |
| Diabetes mellitus | E136 | 8843126 | Viral diabetes mellitus with other specified complications |
| Diabetes mellitus | E136 | 8843381 | Pancreatogenous diabetes with complications |
| Diabetes mellitus | E136 | 8843394 | Steroid diabetes with complications |
| Diabetes mellitus | E136 | 8843454 | Secondary diabetes with complications |
| Diabetes mellitus | E136 | 8843625 | Drug-induced diabetes with complications |
| Diabetes mellitus | E136 | 8849592 | Hepatic diabetes mellitus with complications |
| Diabetes mellitus | E137 | 8843125 | Viral diabetes mellitus with multiple complications |
| Diabetes mellitus | E137 | 8843380 | Pancreatogenous diabetes with multiple complications |
| Diabetes mellitus | E137 | 8843393 | Steroid diabetes with multiple complications |
| Diabetes mellitus | E137 | 8843453 | Secondary diabetes with multiple complications |
| Diabetes mellitus | E137 | 8843624 | Drug-induced diabetes with multiple complications |

| **Category** | **ICD10** | **Diagnosis Code** | **Diagnosis Name** |
| --- | --- | --- | --- |
| Diabetes mellitus | E137 | 8849591 | Hepatic diabetes mellitus with multiple complications |
| Diabetes mellitus | E139 | 8843127 | Viral diabetes mellitus without complications |
| Diabetes mellitus | E139 | 8843382 | Pancreatogenous diabetes without complications |
| Diabetes mellitus | E139 | 8843395 | Steroid diabetes without complications |
| Diabetes mellitus | E139 | 8843455 | Secondary diabetes without complications |
| Diabetes mellitus | E139 | 8843626 | Drug-induced diabetes without complications |
| Diabetes mellitus | E139 | 8849593 | Hepatic diabetes mellitus without complications |
| Diabetes mellitus | E14 | 2500013 | Diabetes mellitus |
| Diabetes mellitus | E14 | 2507028 | Diabetic complications |
| Diabetes mellitus | E140 | 2502004 | Hyperosmolar nonketotic coma |
| Diabetes mellitus | E140 | 2502006 | Diabetic coma |
| Diabetes mellitus | E140 | 8838076 | Diabetic hypoglycemic coma |
| Diabetes mellitus | E141 | 2501002 | Diabetic acidosis |
| Diabetes mellitus | E141 | 2501003 | Diabetic acetonemia |
| Diabetes mellitus | E141 | 2501005 | Diabetic ketoacidosis |
| Diabetes mellitus | E141 | 8849181 | Diabetic ketosis |
| Diabetes mellitus | E142 | 2503005 | Diabetic renal disease |
| Diabetes mellitus | E142 | 2503007 | Diabetic kidney failure |
| Diabetes mellitus | E142 | 8832747 | Kimmelstiel-Wilson syndrome |
| Diabetes mellitus | E142 | 8838071 | Diabetic renal sclerosis |
| Diabetes mellitus | E142 | 8850065 | Diabetic renal disease |
| Diabetes mellitus | E143 | 2504004 | Diabetic iritis |
| Diabetes mellitus | E143 | 2504005 | Diabetic central retinopathy |
| Diabetes mellitus | E143 | 2504006 | Diabetic cataract |
| Diabetes mellitus | E143 | 2504010 | Proliferative retinopathy diabetic |
| Diabetes mellitus | E143 | 2504012 | Diabetic maculopathy |
| Diabetes mellitus | E143 | 2504013 | Retinopathy diabetic |
| Diabetes mellitus | E143 | 8838065 | Diabetic ophthalmoplegia |
| Diabetes mellitus | E143 | 8844089 | Diabetic macular edema |
| Diabetes mellitus | E144 | 2505011 | Diabetic neuralgia |
| Diabetes mellitus | E144 | 2505018 | Diabetic peripheral neuropathy |
| Diabetes mellitus | E144 | 2505021 | Diabetic amyotrophy |
| Diabetes mellitus | E144 | 8838069 | Diabetic neuropathic bladder |
| Diabetes mellitus | E144 | 8838070 | Diabetic autonomic neuropathy |
| Diabetes mellitus | E144 | 8838074 | Diabetic polyneuropathy |
| Diabetes mellitus | E144 | 8838075 | Diabetic mononeuropathy |
| Diabetes mellitus | E144 | 8838078 | Diabetic neuropathy |
| Diabetes mellitus | E144 | 8848634 | Diabetic foot lesions |
| Diabetes mellitus | E144 | 8848768 | Diabetic neuropathic pain |
| Diabetes mellitus | E145 | 2506006 | Diabetic gangrene |
| Diabetes mellitus | E145 | 2506011 | Diabetic arterial occlusion |
| Diabetes mellitus | E145 | 8838063 | Diabetic ulcer |
| Diabetes mellitus | E145 | 8838066 | Diabetic vascular disorder |
| Diabetes mellitus | E145 | 8838077 | Diabetic arteriosclerosis |
| Diabetes mellitus | E145 | 8838079 | Diabetic peripheral angiopathy |
| Diabetes mellitus | E145 | 8838080 | Diabetic peripheral vascular disease |
| Diabetes mellitus | E145 | 8848632 | Diabetic foot gangrene |

| **Category** | **ICD10** | **Diagnosis Code** | **Diagnosis Name** |
| --- | --- | --- | --- |
| Diabetes mellitus | E145 | 8848633 | Diabetic foot ulcers |
| Diabetes mellitus | E146 | 2507025 | Diabetic arthropathy |
| Diabetes mellitus | E146 | 2507029 | Diabetic dermopathy |
| Diabetes mellitus | E146 | 8838064 | Diabetic hepatopathy |
| Diabetes mellitus | E146 | 8838067 | Diabetic hypercholesteremia |
| Diabetes mellitus | E146 | 8838068 | Diabetic osteosis |
| Diabetes mellitus | E146 | 8838072 | Diabetic psychic disturbance |
| Diabetes mellitus | E146 | 8838073 | Diabetic pruritus |
| Diabetes mellitus | E146 | 8844652 | Diabetic blister |
| Diabetes mellitus | E146 | 8844653 | Scleroedema diabeticorum |
| Diabetes mellitus | E146 | 8845128 | Hyperglycemic hyperosmolar syndrome |
| Diabetes mellitus | E149 | 8843439 | Diabetes mellitus without complications |
| Possible diagnoses for hypoglycemia | E100 | 8830030 | Type 1 diabetic coma |
| Possible diagnoses for hypoglycemia | E100 | 8841679 | Type 1 diabetes mellitus with coma |
| Possible diagnoses for hypoglycemia | E100 | 8844026 | Slowly progressive type 1 diabetes mellitus with coma |
| Possible diagnoses for hypoglycemia | E100 | 8845065 | Type 1 diabetic hypoglycemic coma |
| Possible diagnoses for hypoglycemia | E110 | 8830041 | Type 2 diabetic coma |
| Possible diagnoses for hypoglycemia | E110 | 8841689 | Type 2 diabetes mellitus with coma |
| Possible diagnoses for hypoglycemia | E110 | 8845094 | Type 2 diabetic hypoglycemic coma |
| Possible diagnoses for hypoglycemia | E140 | 2502004 | Hyperosmolar nonketotic coma |
| Possible diagnoses for hypoglycemia | E140 | 2502006 | Diabetic coma |
| Possible diagnoses for hypoglycemia | E140 | 8838076 | Diabetic hypoglycemic coma |
| Possible diagnoses for hypoglycemia | E15 | 2510003 | Hypoglycemic coma |
| Possible diagnoses for hypoglycemia | E15 | 8839324 | Nondiabetic hypoglycemic coma |
| Possible diagnoses for hypoglycemia | E160 | 2512009 | Iatrogenic hypoglycemia |
| Possible diagnoses for hypoglycemia | E160 | 8830649 | Insulin hypoglycemia |
| Possible diagnoses for hypoglycemia | E161 | 2511007 | Hyperinsulinemia |
| Possible diagnoses for hypoglycemia | E161 | 8834798 | Idiopathic ketotic hypoglycemia in children |
| Possible diagnoses for hypoglycemia | E161 | 8837872 | Hypoglycemic encephalopathy |
| Possible diagnoses for hypoglycemia | E161 | 8838149 | Pancreatic beta-cell hyperplasia |

| **Category** | **ICD10** | **Diagnosis Code** | **Diagnosis Name** |
| --- | --- | --- | --- |
| Possible diagnoses for hypoglycemia | E161 | 8842161 | Insulin autoimmune syndrome |
| Possible diagnoses for hypoglycemia | E161 | 8844963 | Ketotic hypoglycemia |
| Possible diagnoses for hypoglycemia | E161 | 8849798 | Congenital hyperinsulinemia |
| Possible diagnoses for hypoglycemia | E162 | 2512004 | Hypoglycemia episode |
| Possible diagnoses for  hypoglycemia | E162 | 8837871 | Hypoglycemia |
| Possible diagnoses for hypoglycemia | E162 | 8840698 | Hypoglycemia night |
| Charlson Score | B200 | 8843639 | HIV nontuberculous mycobacteriosis |
| Charlson Score | B202 | 8830096 | HIV cytomegaloviral infections |
| Charlson Score | B203 | 8849060 | HIV herpes viral infections |
| Charlson Score | B204 | 8830094 | HIV candidiasis |
| Charlson Score | B206 | 8830092 | HIV-related pneumocystis carinii pneumonia |
| Charlson Score | B210 | 8830091 | HIV-associated Kaposi’s sarcoma |
| Charlson Score | B211 | 8830099 | HIV-associated Burkitt lymphoma |
| Charlson Score | B212 | 8830100 | HIV-related non-Hodgkin’s lymphoma |
| Charlson Score | B220 | 8830098 | HIV encephalopathy |
| Charlson Score | B220 | 8845516 | Dementia due to HIV infection |
| Charlson Score | B221 | 8830093 | HIV-associated pneumonia interstitial |
| Charlson Score | B222 | 8835763 | Slim disease |
| Charlson Score | B230 | 8832271 | Acute HIV syndrome |
| Charlson Score | B238 | 8830097 | HIV-associated nephropathy |
| Charlson Score | B238 | 8844004 | HIV retinopathy |
| Charlson Score | B24 | 0798002 | HIV infection |
| Charlson Score | B24 | 2793007 | Acquired immunodeficiency syndromes |
| Charlson Score | B24 | 2793011 | AIDS |
| Charlson Score | B24 | 7712015 | Neonatal HIV infections |
| Charlson Score | B24 | 8830055 | AIDS-related complex |
| Charlson Score | B24 | 8842156 | HIV infection |
| Charlson Score | B24 | 8847287 | HIV-1 infection |
| Charlson Score | B24 | 8847288 | HIV-2 infection |
| Charlson Score | C000 | 8842597 | Cancer of external upper lip |
| Charlson Score | C001 | 8842560 | Cancer of external lower lip |
| Charlson Score | C002 | 8842579 | Cancer of external lip |
| Charlson Score | C003 | 8835450 | Cancer of upper lip |
| Charlson Score | C003 | 8850013 | Acinic cell carcinoma of upper lip |
| Charlson Score | C003 | 8850014 | Adenoid cystic carcinoma of upper lip |
| Charlson Score | C003 | 8850016 | Mucoepidermoid carcinoma of upper lip |
| Charlson Score | C004 | 8831237 | Cancer of lower lip |
| Charlson Score | C004 | 8849901 | Acinic cell carcinoma of lower lip |
| Charlson Score | C004 | 8849902 | Adenoid cystic carcinoma of lower lip |
| Charlson Score | C004 | 8849904 | Mucoepidermoid carcinoma of lower lip |
| Charlson Score | C006 | 8834979 | Cancer of oral commissure |
| Charlson Score | C008 | 8842578 | Cancer of oral overlapping lesion |
| Charlson Score | C009 | 1409003 | Cancer of lip |

| **Category** | **ICD10** | **Diagnosis Code** | **Diagnosis Name** |
| --- | --- | --- | --- |
| Charlson Score | C01 | 8836431 | Cancer at base of tongue |
| Charlson Score | C020 | 8836461 | Cancer at dorsal surface of tongue |
| Charlson Score | C021 | 8836443 | Cancer at border of tongue |
| Charlson Score | C021 | 8842603 | Tongue cancer at the tip |
| Charlson Score | C022 | 8836428 | Cancer at ventral surface of tongue |
| Charlson Score | C029 | 1419002 | Tongue cancer |
| Charlson Score | C029 | 8844999 | Liposarcoma of the tongue |
| Charlson Score | C030 | 8842594 | Upper gingival cancer |
| Charlson Score | C031 | 8842556 | Lower gingival cancer |
| Charlson Score | C039 | 1439002 | Gingival cancer |
| Charlson Score | C040 | 8846729 | Mouth floor cancer of anterior portion |
| Charlson Score | C040 | 8846730 | Carcinoma of oral floor of anterior portion |
| Charlson Score | C041 | 8846785 | Mouth floor cancer of lateral portion |
| Charlson Score | C041 | 8846786 | Mouth floor cancer of lateral portion |
| Charlson Score | C049 | 1449002 | Mouth floor cancer |
| Charlson Score | C049 | 8844192 | Carcinoma of oral floor |
| Charlson Score | C050 | 8833430 | Carcinoma of hard palate |
| Charlson Score | C050 | 8849959 | Acinic cell carcinoma of hard palate |
| Charlson Score | C050 | 8849960 | Adenoid cystic carcinoma of hard palate |
| Charlson Score | C050 | 8849962 | Mucoepidermoid carcinoma of hard palate |
| Charlson Score | C051 | 8838349 | Soft palate cancer |
| Charlson Score | C051 | 8850072 | Acinic cell carcinoma of soft palate |
| Charlson Score | C051 | 8850073 | Adenoid cystic carcinoma of soft palate |
| Charlson Score | C051 | 8850074 | Mucoepidermoid carcinoma of soft palate |
| Charlson Score | C052 | 8833368 | Uvular cancer |
| Charlson Score | C059 | 8833363 | Palatal cancer |
| Charlson Score | C059 | 8849955 | Acinic cell carcinoma of palate |
| Charlson Score | C059 | 8849956 | Adenoid cystic carcinoma of palate |
| Charlson Score | C059 | 8849957 | Mucoepidermoid carcinoma of palate |
| Charlson Score | C060 | 8832563 | Buccal mucosa cancer |
| Charlson Score | C060 | 8849929 | Acinic cell carcinoma of buccal mucosa |
| Charlson Score | C060 | 8849930 | Adenoid cystic carcinoma of buccal mucosa |
| Charlson Score | C060 | 8849932 | Mucoepidermoid carcinoma of buccal mucosa |
| Charlson Score | C061 | 8833402 | Buccal cavity cancer |
| Charlson Score | C061 | 8842557 | Lower gingival cancer of mucogingival junction |
| Charlson Score | C061 | 8842595 | Upper gingival cancer of mucogingival junction |
| Charlson Score | C062 | 8832258 | Cancer of retromolar area |
| Charlson Score | C062 | 8842593 | Cancer of maxillary tuberosity |
| Charlson Score | C069 | 1459007 | Oral cancer |
| Charlson Score | C069 | 8834757 | Small salivary gland cancers |
| Charlson Score | C069 | 8843320 | Oral melanoma malignant |
| Charlson Score | C069 | 8849986 | Acinic cell carcinoma of small salivary gland |
| Charlson Score | C069 | 8849987 | Adenoid cystic carcinoma of small salivary gland |
| Charlson Score | C069 | 8849989 | Mucoepidermoid carcinoma of small salivary gland |
| Charlson Score | C07 | 1420003 | Parotid gland cancer |
| Charlson Score | C07 | 8849993 | Acinic cell carcinoma of parotid gland |
| Charlson Score | C07 | 8849994 | Adenoid cystic carcinoma of parotid gland |

| **Category** | **ICD10** | **Diagnosis Code** | **Diagnosis Name** |
| --- | --- | --- | --- |
| Charlson Score | C07 | 8849995 | Mucoepidermoid carcinoma of parotid gland |
| Charlson Score | C080 | 1421002 | Submandibular gland cancer |
| Charlson Score | C080 | 8849923 | Acinic cell carcinoma of submandibular gland |
| Charlson Score | C080 | 8849924 | Adenoid cystic carcinoma of submandibular gland |
| Charlson Score | C080 | 8849925 | Mucoepidermoid carcinoma of submandibular gland |
| Charlson Score | C081 | 1422003 | Sublingual gland cancer |
| Charlson Score | C081 | 8850028 | Acinic cell carcinoma of sublingual gland |
| Charlson Score | C081 | 8850029 | Adenoid cystic carcinoma of sublingual gland |
| Charlson Score | C081 | 8850031 | Mucoepidermoid carcinoma of sublingual gland |
| Charlson Score | C089 | 1429007 | Salivary gland cancer |
| Charlson Score | C089 | 8837362 | Major salivary gland cancers |
| Charlson Score | C089 | 8850059 | Acinic cell carcinoma of salivary gland |
| Charlson Score | C089 | 8850060 | Adenoid cystic carcinoma of salivary gland |
| Charlson Score | C089 | 8850061 | Mucoepidermoid carcinoma of salivary gland |
| Charlson Score | C090 | 8840005 | Tonsillar Fossa Cancer |
| Charlson Score | C091 | 8833364 | Palatine arch cancer |
| Charlson Score | C099 | 1460003 | Tonsillar carcinoma |
| Charlson Score | C099 | 1460004 | Tonsillar sarcoma |
| Charlson Score | C100 | 8833609 | Epiglottic vallecula carcinoma |
| Charlson Score | C101 | 8833605 | Epiglottic carcinoma at anterior surface |
| Charlson Score | C102 | 8837552 | Oropharyngeal cancer at Lateral wall |
| Charlson Score | C103 | 8846010 | Oropharyngeal cancer at posterior wall |
| Charlson Score | C104 | 8833941 | Branchiogenous ccarcinoma |
| Charlson Score | C109 | 1469004 | Oropharyngeal cancer |
| Charlson Score | C109 | 8837553 | Oropharyngeal sarcoma |
| Charlson Score | C110 | 8835376 | Nasopharyngeal carcinoma at superior wall |
| Charlson Score | C111 | 8835375 | Nasopharyngeal carcinoma at posterior wall |
| Charlson Score | C112 | 8835378 | Nasopharyngeal carcinoma at lateral wall |
| Charlson Score | C113 | 8835377 | Nasopharyngeal carcinoma at anterior wall |
| Charlson Score | C119 | 1479001 | Nasopharyngeal carcinoma |
| Charlson Score | C119 | 1479002 | Nasopharyngeal cancer |
| Charlson Score | C119 | 1611001 | Nasopharyngeal malignant tumor |
| Charlson Score | C119 | 8844986 | Nasopharyngeal Liposarcoma |
| Charlson Score | C12 | 8840960 | Pyriform sinus carcinoma |
| Charlson Score | C130 | 8841093 | Postcricoid cancer |
| Charlson Score | C131 | 8847687 | Aryepiglottic fold cancer of hypopharynx |
| Charlson Score | C131 | 8847727 | Hypopharyngeal surface cancer of aryepiglottic fold |
| Charlson Score | C132 | 8831084 | Hypopharyngeal cancer at posterior wall |
| Charlson Score | C139 | 1489003 | Hypopharyngeal cancer |
| Charlson Score | C139 | 8831087 | Hypopharyngeal sarcoma |
| Charlson Score | C140 | 1469001 | Pharyngeal sarcoma |
| Charlson Score | C140 | 1490001 | Pharyngeal cancer |
| Charlson Score | C140 | 8847137 | Malignant tumor of parapharyngeal space |
| Charlson Score | C150 | 1500002 | Cervical esophageal carcinoma |
| Charlson Score | C151 | 1501002 | Thoracic esophageal carcinoma |
| Charlson Score | C151 | 8842701 | Esophageal cancer of lower chest |

| **Category** | **ICD10** | **Diagnosis Code** | **Diagnosis Name** |
| --- | --- | --- | --- |
| Charlson Score | C151 | 8842702 | Esophageal cancer of upper chest |
| Charlson Score | C151 | 8842703 | Esophageal cancer of central chest |
| Charlson Score | C152 | 1502002 | Abdominal esophagus carcinoma |
| Charlson Score | C153 | 1503002 | Upper esophageal cancer |
| Charlson Score | C154 | 1504002 | Middle esophageal cancer |
| Charlson Score | C155 | 1505002 | Lower esophageal cancer |
| Charlson Score | C155 | 8844316 | Barrett’s esophageal cancer |
| Charlson Score | C158 | 8848065 | Gastrooesophageal junction cancer |
| Charlson Score | C159 | 1509003 | Oesophageal cancer |
| Charlson Score | C159 | 1509006 | Early esophageal cancer |
| Charlson Score | C159 | 8834848 | Esophageal carcinosarcoma |
| Charlson Score | C159 | 8834863 | Esophageal leiomyosarcoma |
| Charlson Score | C159 | 8842744 | Malignant melanoma of the esophagus |
| Charlson Score | C159 | 8842745 | Esophageal rhabdomyosarcoma |
| Charlson Score | C159 | 8842747 | Esophageal carcinoid |
| Charlson Score | C159 | 8842748 | Basal cell carcinoma of the esophagus |
| Charlson Score | C159 | 8842749 | Esophageal pseudosarcoma |
| Charlson Score | C159 | 8842751 | Small cell carcinoma of the oesophagus |
| Charlson Score | C159 | 8842753 | Adenocarcinoma of the oesophagus |
| Charlson Score | C159 | 8842754 | Adenoid cystic carcinoma of the oesophagus |
| Charlson Score | C159 | 8842755 | Mucoepidermoid carcinoma of the esophagus |
| Charlson Score | C159 | 8842756 | Superficial cancer of the oesophagus |
| Charlson Score | C159 | 8842757 | Anaplastic carcinoma of the oesophagus |
| Charlson Score | C159 | 8844982 | Esophageal liposarcoma |
| Charlson Score | C159 | 8845202 | KIT (CD117) -positive esophageal stromal tumor |
| Charlson Score | C159 | 8846289 | Malignant emesenchymal tumor of the oesophagus |
| Charlson Score | C159 | 8847830 | KIT (CD117) -positive esophageal and gastrointestinal stromal tumor |
| Charlson Score | C159 | 8847859 | Esophageal and gastrointestinal stromal tumor |
| Charlson Score | C159 | 8849124 | Neuroendocrine cell carcinoma of the oesophagus |
| Charlson Score | C159 | 8849125 | Neuroendocrine tumor of the oesophagus |
| Charlson Score | C160 | 1510005 | Cardia carcinoma |
| Charlson Score | C160 | 8849679 | Cardioesophageal junction cancer |
| Charlson Score | C161 | 1513002 | Gastric fundus cancer |
| Charlson Score | C162 | 1514002 | Cancer in the gastric body |
| Charlson Score | C163 | 1512002 | Pyloric antrum carcinoma |
| Charlson Score | C163 | 8848024 | Gastric antral carcinoma |
| Charlson Score | C164 | 1511003 | Pyloric carcinoma |
| Charlson Score | C164 | 8830612 | Pyloric carcinoma of the stomach |
| Charlson Score | C165 | 8845849 | Lesser curvature of stomach carcinoma |
| Charlson Score | C166 | 8845852 | Greater curvature of stomach carcinoma |
| Charlson Score | C169 | 1519006 | Gastric carcinoma |
| Charlson Score | C169 | 1519010 | End stage gastric cancer |
| Charlson Score | C169 | 1519011 | Gastric double cancer |
| Charlson Score | C169 | 1519012 | Advanced gastric cancer |
| Charlson Score | C169 | 1519015 | Gastric sarcoma |

| **Category** | **ICD10** | **Diagnosis Code** | **Diagnosis Name** |
| --- | --- | --- | --- |
| Charlson Score | C169 | 1519017 | Gastric leiomyosarcoma |
| Charlson Score | C169 | 1519020 | Residual gastric cancer |
| Charlson Score | C169 | 1519022 | Early gastric cancer |
| Charlson Score | C169 | 8830411 | Malignant melanoma of the stomach |
| Charlson Score | C169 | 8830421 | Gastric carcinoid |
| Charlson Score | C169 | 8842111 | Scirrhous gastric carcinoma |
| Charlson Score | C169 | 8842666 | Gastric tube cancer |
| Charlson Score | C169 | 8844932 | Gastric liposarcoma |
| Charlson Score | C169 | 8845199 | KIT (CD117) -positive gastric stromal tumor |
| Charlson Score | C169 | 8846240 | Malignant emesenchymal tumor of the stomach |
| Charlson Score | C169 | 8846354 | Gastric carcinoma-HER2 overexpression |
| Charlson Score | C169 | 8847306 | Primary gastric choriocarcinoma |
| Charlson Score | C169 | 8847307 | Gastric germ cell tumor |
| Charlson Score | C169 | 8847827 | KIT (CD117) -positive gastrointestinal stromal tumor |
| Charlson Score | C169 | 8847839 | Gastrointestinal stromal tumor |
| Charlson Score | C169 | 8848457 | Endocrine cell carcinoma of the stomach |
| Charlson Score | C169 | 8849064 | Neuroendocrine cell carcinoma of the stomach |
| Charlson Score | C169 | 8849065 | Neuroendocrine tumor of the stomach |
| Charlson Score | C169 | 8849698 | HER2 positive gastric cancer |
| Charlson Score | C170 | 1520002 | Duodenal carcinoma |
| Charlson Score | C170 | 1520006 | Duodenal carcinoid |
| Charlson Score | C170 | 8835301 | Duodenal leiomyosarcoma |
| Charlson Score | C170 | 8846398 | Neuroendocrine carcinoma of the duodenum |
| Charlson Score | C170 | 8847202 | Malignant duodenal gastrinoma |
| Charlson Score | C170 | 8847203 | Malignant duodenal somatostatinoma |
| Charlson Score | C170 | 8847871 | Duodenal and gastrointestinal stromal tumor |
| Charlson Score | C170 | 8849135 | Neuroendocrine cell carcinoma of the duodenum |
| Charlson Score | C171 | 1521002 | Jejunum cancer |
| Charlson Score | C171 | 8846572 | Jejunal carcinoid |
| Charlson Score | C171 | 8847854 | Jejunal and gastrointestinal stromal tumor |
| Charlson Score | C171 | 8849102 | Neuroendocrine cell carcinoma of the jejunum |
| Charlson Score | C171 | 8849103 | Neuroendocrine tumor of the jejunum |
| Charlson Score | C172 | 1522002 | Ileal cancer |
| Charlson Score | C172 | 8846480 | Ileal carcinoid |
| Charlson Score | C172 | 8847842 | Ileal and GI stromal tumor |
| Charlson Score | C172 | 8849078 | Neuroendocrine cell carcinoma of the ileum |
| Charlson Score | C172 | 8849079 | Neuroendocrine tumor of the ileum |
| Charlson Score | C179 | 1529002 | Small intestine carcinoma |
| Charlson Score | C179 | 8844981 | Small intestine liposarcoma |
| Charlson Score | C179 | 8845201 | KIT (CD117) -positive small intestinal stromal tumor |
| Charlson Score | C179 | 8845951 | Small intestine leiomyosarcoma |
| Charlson Score | C179 | 8846657 | Small intestine carcinoid |
| Charlson Score | C179 | 8847829 | KIT (CD117) -positive small intestine and gastrointestinal stromal tumor |
| Charlson Score | C179 | 8847858 | Small intestine and gastrointestinal stromal tumor |

| **Category** | **ICD10** | **Diagnosis Code** | **Diagnosis Name** |
| --- | --- | --- | --- |
| Charlson Score | C179 | 8849122 | Neuroendocrine cell carcinoma of the small intestine |
| Charlson Score | C179 | 8849123 | Neuroendocrine tumor of the small intestine |
| Charlson Score | C180 | 1534001 | Ileocecal carcinoma |
| Charlson Score | C180 | 1534004 | Cecal cancer |
| Charlson Score | C180 | 8844619 | Cecal carcinoid |
| Charlson Score | C180 | 8848675 | Endocrine cell carcinoma of the cecal |
| Charlson Score | C180 | 8849216 | Neuroendocrine cell carcinoma of the cecal |
| Charlson Score | C180 | 8849217 | Neuroendocrine tumor of the cecal |
| Charlson Score | C181 | 1535002 | Carcinoma of the appendix |
| Charlson Score | C181 | 8830219 | Mucinous carcinoma of the appendix |
| Charlson Score | C181 | 8847052 | Goblet cell carcinoid of the appendix |
| Charlson Score | C181 | 8849175 | Neuroendocrine tumor of the appendix |
| Charlson Score | C182 | 1536002 | Ascending colon carcinoma |
| Charlson Score | C182 | 8835410 | Leiomyosarcoma of the ascending colon |
| Charlson Score | C182 | 8844478 | Carcinoid of the ascending colon |
| Charlson Score | C182 | 8849137 | Neuroendocrine cell carcinoma of the ascending colon |
| Charlson Score | C182 | 8849138 | Neuroendocrine tumor of the ascending colon |
| Charlson Score | C183 | 8831682 | Hepatic flexure carcinoma |
| Charlson Score | C184 | 1531002 | Cancer of the transverse colon |
| Charlson Score | C184 | 8849076 | Neuroendocrine cell carcinoma of the transverse colon |
| Charlson Score | C184 | 8849077 | Neuroendocrine tumor of the transverse colon |
| Charlson Score | C185 | 8839429 | Splenic flexure carcinoma |
| Charlson Score | C186 | 1532002 | Descending colon carcinoma |
| Charlson Score | C186 | 8849082 | Neuroendocrine cell carcinoma of the descending colon |
| Charlson Score | C186 | 8849083 | Neuroendocrine tumor of the descending colon |
| Charlson Score | C187 | 1533003 | Sigmoid colon cancer |
| Charlson Score | C187 | 8849061 | Neuroendocrine cell carcinoma of the sigmoid colon |
| Charlson Score | C187 | 8849062 | Neuroendocrine tumor of the sigmoid colon |
| Charlson Score | C189 | 1539002 | Colon cancer |
| Charlson Score | C189 | 1539004 | Large intestine carcinoma |
| Charlson Score | C189 | 8837365 | Colonic carcinoid |
| Charlson Score | C189 | 8837377 | Colonic sarcoma |
| Charlson Score | C189 | 8842670 | Hereditary large intestine carcinoma |
| Charlson Score | C189 | 8842671 | Hereditary nonpolyposis colorectal cancer |
| Charlson Score | C189 | 8842802 | Mucinous carcinoma of the large intestine |
| Charlson Score | C189 | 8844962 | Colonic liposarcoma |
| Charlson Score | C189 | 8845200 | KIT (CD117) -positive colonic stromal tumor |
| Charlson Score | C189 | 8847828 | KIT (CD117) -positive colonic and gastrointestinal stromal tumor |
| Charlson Score | C189 | 8847856 | Colonic and gastrointestinal stromal tumor |
| Charlson Score | C189 | 8847915 | KRAS-gene wild-type colonic cancer |
| Charlson Score | C189 | 8849104 | Neuroendocrine cell carcinoma of the colon |
| Charlson Score | C189 | 8849105 | Neuroendocrine tumor of the colon |
| Charlson Score | C189 | 8849167 | Neuroendocrine cell carcinoma of the colon |

| **Category** | **ICD10** | **Diagnosis Code** | **Diagnosis Name** |
| --- | --- | --- | --- |
| Charlson Score | C189 | 8849168 | Neuroendocrine tumor of the colon |
| Charlson Score | C19 | 8842808 | Rectosigmoid cancer |
| Charlson Score | C19 | 8848749 | Rectosigmoid malignant tumor |
| Charlson Score | C20 | 1541005 | Rectal carcinoma |
| Charlson Score | C20 | 1541009 | Postoperative recurrent rectal cancer |
| Charlson Score | C20 | 1541010 | Rectal carcinoma-perforation |
| Charlson Score | C20 | 8837776 | Rectal malignant melanoma |
| Charlson Score | C20 | 8837779 | Rectal carcinoid |
| Charlson Score | C20 | 8845017 | Rectal liposarcoma |
| Charlson Score | C20 | 8845203 | KIT (CD117) -positive rectal stromal tumor |
| Charlson Score | C20 | 8846018 | Rectal leiomyosarcoma |
| Charlson Score | C20 | 8847831 | KIT (CD117) -positive rectal and gastrointestinal stromal tumor |
| Charlson Score | C20 | 8847886 | Rectal and gastrointestinal stromal tumor |
| Charlson Score | C20 | 8847916 | KRAS-gene wild-type rectal cancer |
| Charlson Score | C20 | 8849176 | Neuroendocrine cell carcinoma of the rectum |
| Charlson Score | C20 | 8849177 | Neuroendocrine tumor of the rectum |
| Charlson Score | C210 | 1543002 | Anal carcinoma |
| Charlson Score | C210 | 1543003 | Anal cancer |
| Charlson Score | C210 | 8842719 | Malignant melanoma of the anus |
| Charlson Score | C210 | 8842722 | Sq cell Ca anus |
| Charlson Score | C211 | 8833700 | Anal canal carcinoma |
| Charlson Score | C211 | 8842315 | Fistula cancer |
| Charlson Score | C220 | 1550004 | Liver/hepatic carcinoma |
| Charlson Score | C220 | 1550005 | Hepatocellular carcinoma |
| Charlson Score | C220 | 1550012 | Primary hepatic carcinoma |
| Charlson Score | C220 | 8847692 | Hepatocellular carcinoma-rupture |
| Charlson Score | C221 | 1551002 | Intrahepatic bile duct carcinoma |
| Charlson Score | C221 | 1561005 | Cholangiocarcinoma |
| Charlson Score | C222 | 8831496 | Hepatoblastoma |
| Charlson Score | C223 | 8831501 | Hepatic angiosarcoma |
| Charlson Score | C224 | 8831674 | Hepatic leiomyosarcoma |
| Charlson Score | C224 | 8844946 | Hepatic liposarcoma |
| Charlson Score | C224 | 8849015 | Hepatoembryonic sarcoma |
| Charlson Score | C227 | 8831498 | Hepatic teratoma |
| Charlson Score | C227 | 8831666 | Hepatic cystadenocarcinoma |
| Charlson Score | C227 | 8842916 | Mixed hepatocellular carcinoma |
| Charlson Score | C229 | 8831475 | Malignant hepatic tumor |
| Charlson Score | C229 | 8831492 | Hepatic carcinoid |
| Charlson Score | C229 | 8831679 | Hepatic porta carcinoma |
| Charlson Score | C23 | 8837166 | Gallbladder carcinoma |
| Charlson Score | C23 | 8837187 | Gallbladder sarcoma |
| Charlson Score | C23 | 8846805 | Gallbladder carcinoid |
| Charlson Score | C23 | 8848215 | Neuroendocrine carcinoma of the gallbladder |
| Charlson Score | C240 | 1561004 | Bile duct cancer |
| Charlson Score | C240 | 8831494 | Extrahepatic bile duct cancer |
| Charlson Score | C240 | 8835701 | Intrapancreatic bile duct carcinoma |
| Charlson Score | C240 | 8836676 | Common bile duct cancer |
| Charlson Score | C240 | 8837158 | Cholangiocarcinoma |

| **Category** | **ICD10** | **Diagnosis Code** | **Diagnosis Name** |
| --- | --- | --- | --- |
| Charlson Score | C240 | 8842690 | Lower bile duct cancer |
| Charlson Score | C240 | 8842770 | Upper bile duct cancer |
| Charlson Score | C240 | 8842807 | Central bile duct cancer |
| Charlson Score | C240 | 8849880 | Distal cholangiocarcinoma |
| Charlson Score | C241 | 1520003 | Duodenal papilla cancer |
| Charlson Score | C241 | 1520004 | Duodenal papilla cancer |
| Charlson Score | C248 | 8841644 | Hepatic porta cholangiocarcinoma |
| Charlson Score | C249 | 1569002 | Biliary carcinoma |
| Charlson Score | C250 | 1570002 | Cancer of the pancreatic head |
| Charlson Score | C250 | 8846726 | Carcinoid of the pancreatic head |
| Charlson Score | C250 | 8848334 | Groove pancreatic carcinoma |
| Charlson Score | C251 | 1571002 | Pancreatic body cancer |
| Charlson Score | C252 | 8835710 | Pancreatic tail cancer |
| Charlson Score | C253 | 8835653 | Pancreatic ductal carcinoma |
| Charlson Score | C253 | 8844259 | Intraductal tubular adenocarcinoma of the pancreas |
| Charlson Score | C253 | 8844262 | Intraductal papillary-mucinous carcinoma of the pancreas |
| Charlson Score | C254 | 1574005 | Pancreatic islet cell carcinoma |
| Charlson Score | C254 | 8830206 | Malignant insulinoma |
| Charlson Score | C254 | 8830216 | Malignant endocrine tumor of the pancreas |
| Charlson Score | C254 | 8844929 | VIP-producing tumor |
| Charlson Score | C254 | 8845115 | Malignant somatostatinoma |
| Charlson Score | C254 | 8847155 | Malignant gastrinoma |
| Charlson Score | C254 | 8847156 | Malignant glucagonoma |
| Charlson Score | C257 | 8835659 | Pancreatic neck carcinoma |
| Charlson Score | C258 | 8844267 | Pancreatic body and tail cancer |
| Charlson Score | C259 | 1579002 | Pancreas carcinoma |
| Charlson Score | C259 | 8842774 | Acinar cell carcinoma of the pancreas |
| Charlson Score | C259 | 8843374 | Pancreatoblastoma |
| Charlson Score | C259 | 8844265 | Serous cystadenocarcinoma of the pancreas |
| Charlson Score | C259 | 8844269 | Mucinous cystadenocarcinoma of the pancreas |
| Charlson Score | C259 | 8844991 | Pancreatic liposarcoma |
| Charlson Score | C259 | 8849134 | Solid pseudopapillary tumor |
| Charlson Score | C261 | 8839345 | Spleen malignant tumor |
| Charlson Score | C261 | 8845030 | Splenic liposarcoma |
| Charlson Score | C269 | 2355001 | Gastrointestinal carcinoid |
| Charlson Score | C269 | 8842453 | KIT (CD117) -positive gastrointestinal stromal tumor |
| Charlson Score | C269 | 8845853 | Imatinib-resistant GI stromal tumor |
| Charlson Score | C269 | 8847841 | Imatinib-resistant GI stromal tumor |
| Charlson Score | C300 | 1600003 | Nasal cavity cancer |
| Charlson Score | C300 | 2251005 | Olfactory neuroblastoma |
| Charlson Score | C300 | 2251006 | Olfactory neuroepithelioma |
| Charlson Score | C300 | 8839482 | Nasal vestibular cancer |
| Charlson Score | C300 | 8839511 | Nasal septal cancer |
| Charlson Score | C301 | 1601004 | Middle ear malignant tumor |
| Charlson Score | C301 | 8835192 | Eustachian tube cancer |
| Charlson Score | C301 | 8838307 | Inner ear cancer |

| **Category** | **ICD10** | **Diagnosis Code** | **Diagnosis Name** |
| --- | --- | --- | --- |
| Charlson Score | C301 | 8848402 | Endolymphatic tumor |
| Charlson Score | C310 | 1602001 | Maxillary cancer |
| Charlson Score | C310 | 8835397 | Cancer of maxillary antrum |
| Charlson Score | C311 | 8834279 | Ethmoidal sinus carcinoma |
| Charlson Score | C312 | 8836560 | Frontal sinus carcinoma |
| Charlson Score | C313 | 8837710 | Sphenoidal sinus carcinoma |
| Charlson Score | C319 | 1609002 | Paranasal sinus cancer |
| Charlson Score | C320 | 8835961 | Glottic carcinoma |
| Charlson Score | C321 | 1463002 | Epiglottic carcinoma |
| Charlson Score | C321 | 8831259 | False vocal cord cancer |
| Charlson Score | C321 | 8835963 | Supraglottic cancer |
| Charlson Score | C321 | 8847728 | Plicated laryngeal surface cancer of the cleft aryepiglottic fold |
| Charlson Score | C322 | 8835959 | Subglottic cancer |
| Charlson Score | C323 | 8833529 | Thyroid cartilage malignant tumor |
| Charlson Score | C329 | 1619005 | Laryngeal carcinoma |
| Charlson Score | C33 | 1620003 | Tracheal cancer |
| Charlson Score | C340 | 8834569 | Main bronchus malignant tumor |
| Charlson Score | C340 | 8842835 | Pulmonary hilar cancer |
| Charlson Score | C340 | 8847675 | Hilar small-cell lung carcinoma |
| Charlson Score | C340 | 8847676 | Hilar lung adenocarcinoma |
| Charlson Score | C340 | 8847677 | Hilar large cell carcinoma |
| Charlson Score | C340 | 8847678 | Hilar non-small cell carcinoma |
| Charlson Score | C340 | 8847679 | Hilar squamous cell carcinoma |
| Charlson Score | C341 | 8835493 | Upper lobe lung cancer |
| Charlson Score | C341 | 8845183 | Pancoast’s syndrome |
| Charlson Score | C341 | 8847633 | Upper lobe small-cell lung cancer |
| Charlson Score | C341 | 8847634 | Upper lobe pulmonary adenocarcinoma |
| Charlson Score | C341 | 8847635 | Upper lobe large-cell lung carcinoma |
| Charlson Score | C341 | 8847636 | Upper lobe squamous cell carcinoma |
| Charlson Score | C341 | 8847637 | Upper lobe non-small cell lung cancer |
| Charlson Score | C342 | 8837666 | Middle lobe lung cancer |
| Charlson Score | C342 | 8847660 | Middle lobe small-cell lung cancer |
| Charlson Score | C342 | 8847661 | Middle lobe lung adenocarcinoma |
| Charlson Score | C342 | 8847662 | Middle lobe large-cell lung carcinoma |
| Charlson Score | C342 | 8847663 | Squamous cell carcinoma in the middle lobe lung |
| Charlson Score | C342 | 8847664 | Middle lobe non-small cell lung cancer |
| Charlson Score | C343 | 8831458 | Lower lobe lung cancer |
| Charlson Score | C343 | 8847594 | Lower lobe small cell lung cancer |
| Charlson Score | C343 | 8847595 | Lower lobe lung adenocarcinoma |
| Charlson Score | C343 | 8847596 | Lower lobe large-cell lung carcinoma |
| Charlson Score | C343 | 8847597 | Squamous cell carcinoma in the lower lobe lung |
| Charlson Score | C343 | 8847598 | Lower lobe non-small cell lung cancer |
| Charlson Score | C348 | 8833932 | Bronchioloalveolar carcinoma |
| Charlson Score | C349 | 1629003 | Primary lung cancer |
| Charlson Score | C349 | 1629006 | Pulmonary carcinoma |
| Charlson Score | C349 | 1629009 | Lung sarcoma |
| Charlson Score | C349 | 8832029 | Cancerous pneumonia |

| **Category** | **ICD10** | **Diagnosis Code** | **Diagnosis Name** |
| --- | --- | --- | --- |
| Charlson Score | C349 | 8832157 | Bronchial carcinoma |
| Charlson Score | C349 | 8838804 | Pulmonary carcinoid |
| Charlson Score | C349 | 8838805 | Pulmonary blastoma |
| Charlson Score | C349 | 8838844 | Lung adenocarcinoma |
| Charlson Score | C349 | 8838852 | Large cell lung cancer |
| Charlson Score | C349 | 8838898 | Squamous cell carcinoma of lung |
| Charlson Score | C349 | 8838901 | Alveolar epithelial cancer |
| Charlson Score | C349 | 8838904 | Anaplastic carcinoma of lung |
| Charlson Score | C349 | 8842053 | Non-small cell lung cancer |
| Charlson Score | C349 | 8842057 | Neuroendocrine large cell carcinoma of lung |
| Charlson Score | C349 | 8842185 | Small cell lung cancer |
| Charlson Score | C349 | 8842831 | Lung cancer sarcoma |
| Charlson Score | C349 | 8842832 | Adenosquamous cell lung cancer |
| Charlson Score | C349 | 8842833 | Adenoid cystic carcinoma of the lung |
| Charlson Score | C349 | 8842834 | Pulmonary mucoepidermoid carcinoma |
| Charlson Score | C349 | 8846553 | Bronchial carcinoid tumor |
| Charlson Score | C349 | 8847242 | Obstructive pneumonia due to lung cancer |
| Charlson Score | C349 | 8847272 | ALK fusion gene-positive non-small cell lung cancer |
| Charlson Score | C349 | 8847732 | EGFR gene mutation-positive non-small cell lung cancer |
| Charlson Score | C349 | 8848775 | Pulmonary choriocarcinoma |
| Charlson Score | C349 | 8849238 | ROS1 fusion gene-positive non-small cell lung cancer |
| Charlson Score | C349 | 8849759 | Pleuropulmonary blastoma in children |
| Charlson Score | C349 | 8849788 | Pleuropulmonary blastoma in adults |
| Charlson Score | C37 | 1640001 | Malignant thymoma |
| Charlson Score | C37 | 1640004 | Thymic carcinoma |
| Charlson Score | C37 | 1640005 | Invasive thymoma |
| Charlson Score | C37 | 2126005 | Thymic carcinoid |
| Charlson Score | C380 | 8835082 | Heart malignant tumor |
| Charlson Score | C380 | 8835085 | Cardiac rhabdomyosarcoma |
| Charlson Score | C380 | 8835087 | Cardiac Hemangiosarcoma |
| Charlson Score | C380 | 8835094 | Cardiac fibrosarcoma |
| Charlson Score | C380 | 8835098 | Cardiac myxosarcoma |
| Charlson Score | C380 | 8844983 | Cardiac liposarcoma |
| Charlson Score | C381 | 8842781 | Anterior mediastinum malignant tumor |
| Charlson Score | C381 | 8848732 | Anterior mediastinal seminoma |
| Charlson Score | C381 | 8848822 | Anterior mediastinal liposarcoma |
| Charlson Score | C382 | 8842717 | Posterior mediastinum malignant tumor |
| Charlson Score | C382 | 8848813 | Posterior mediastinal liposarcoma |
| Charlson Score | C383 | 1649001 | Mediastinum malignant tumor |
| Charlson Score | C383 | 1649004 | Mediastinal Cancer |
| Charlson Score | C383 | 8835273 | Mediastinal neuroblastoma |
| Charlson Score | C383 | 8842805 | Middle mediastinum malignant tumor |
| Charlson Score | C383 | 8844985 | Mediastinal liposarcoma |
| Charlson Score | C383 | 8847365 | Mediastinal germ cell tumor |
| Charlson Score | C383 | 8847366 | Mediastinal yolk sac tumor |
| Charlson Score | C383 | 8848725 | Mediastinal choriocarcinoma |

| **Category** | **ICD10** | **Diagnosis Code** | **Diagnosis Name** |
| --- | --- | --- | --- |
| Charlson Score | C383 | 8848726 | Mediastinal seminoma |
| Charlson Score | C384 | 1639001 | Pleura malignant tumor |
| Charlson Score | C384 | 8844955 | Pleura liposarcoma |
| Charlson Score | C400 | 8846386 | Scapular osteosarcoma |
| Charlson Score | C400 | 8846394 | Ulnar osteosarcoma |
| Charlson Score | C400 | 8846405 | Humeral osteosarcoma |
| Charlson Score | C400 | 8846435 | Radial osteosarcoma |
| Charlson Score | C400 | 8846582 | Ewing’s sarcoma of the scapula |
| Charlson Score | C400 | 8846716 | Ewing’s sarcoma of the humerus |
| Charlson Score | C400 | 8848243 | Scapular chondrosarcoma |
| Charlson Score | C400 | 8848264 | Ulnar chondrosarcoma |
| Charlson Score | C400 | 8848273 | Humeral chondrosarcoma |
| Charlson Score | C400 | 8848296 | Radial chondrosarcoma |
| Charlson Score | C400 | 8849295 | Scapular malignant tumor |
| Charlson Score | C400 | 8849331 | Ulnar malignant tumor |
| Charlson Score | C400 | 8849357 | Humeral malignant tumor |
| Charlson Score | C400 | 8849358 | Distal humeral malignant bone tumor |
| Charlson Score | C400 | 8849360 | Proximal humeral malignant bone tumor |
| Charlson Score | C400 | 8849362 | Malignant bone tumor of the humeral diaphysis |
| Charlson Score | C400 | 8849418 | Radial malignant tumor |
| Charlson Score | C401 | 8846395 | Finger osteosarcoma |
| Charlson Score | C401 | 8848256 | Phalangeal chondrosarcoma |
| Charlson Score | C401 | 8848265 | Carpal bone chondrosarcoma |
| Charlson Score | C401 | 8848288 | Metacarpal chondrosarcoma |
| Charlson Score | C401 | 8849313 | Malignant tumor of the finger proximal phalanx |
| Charlson Score | C401 | 8849317 | Phalangeal malignant tumor |
| Charlson Score | C401 | 8849321 | Malignant tumor of the finger middle phalanx |
| Charlson Score | C401 | 8849328 | Malignant tumor of the finger distal phalanx |
| Charlson Score | C401 | 8849403 | Metacarpal bone malignant tumor |
| Charlson Score | C402 | 8837310 | Femural osteosarcoma |
| Charlson Score | C402 | 8846380 | Proximal tibial osteosarcoma |
| Charlson Score | C402 | 8846381 | Tibial osteosarcoma |
| Charlson Score | C402 | 8846421 | Distal osteosarcoma of the femur |
| Charlson Score | C402 | 8846422 | Proximal osteosarcoma of the femur |
| Charlson Score | C402 | 8846442 | Peroneal osteosarcoma |
| Charlson Score | C402 | 8846573 | Tibial Ewing’s sarcoma |
| Charlson Score | C402 | 8846835 | Femural Ewing’s sarcoma |
| Charlson Score | C402 | 8848240 | Tibial chondrosarcoma |
| Charlson Score | C402 | 8848286 | Femural chondrosarcoma |
| Charlson Score | C402 | 8848301 | Peroneal chondrosarcoma |
| Charlson Score | C402 | 8849282 | Tibial malignant tumor |
| Charlson Score | C402 | 8849283 | Distal tibial malignant bone tumor |
| Charlson Score | C402 | 8849285 | Proximal tibial malignant bone tumor |
| Charlson Score | C402 | 8849287 | Malignant bone tumor of the tibial diaphysis |
| Charlson Score | C402 | 8849392 | Femural malignant tumor |
| Charlson Score | C402 | 8849393 | Distal femoral malignant bone tumor |
| Charlson Score | C402 | 8849395 | Proximal femoral malignant bone tumor |
| Charlson Score | C402 | 8849397 | Malignant bone tumor of the femural diaphysis |
| Charlson Score | C402 | 8849432 | Peroneal malignant tumor |

| **Category** | **ICD10** | **Diagnosis Code** | **Diagnosis Name** |
| --- | --- | --- | --- |
| Charlson Score | C402 | 8849433 | Distal peroneal malignant bone tumor |
| Charlson Score | C402 | 8849435 | Proximal peroneal malignant bone tumor |
| Charlson Score | C402 | 8849437 | Malignant bone tumor of the peroneal diaphysis |
| Charlson Score | C403 | 8846378 | Talus osteosarcoma |
| Charlson Score | C403 | 8846391 | Toe osteosarcoma |
| Charlson Score | C403 | 8846396 | Calcaneal osteosarcoma |
| Charlson Score | C403 | 8848257 | Toe chondrosarcoma |
| Charlson Score | C403 | 8848260 | Patellar chondrosarcoma |
| Charlson Score | C403 | 8848280 | Tarsal bone chondrosarcoma |
| Charlson Score | C403 | 8848290 | Metatarsal chondrosarcoma |
| Charlson Score | C403 | 8849277 | Talus malignant tumor |
| Charlson Score | C403 | 8849312 | Malignant tumor of the foot proximal phalanx |
| Charlson Score | C403 | 8849316 | Toe malignant tumor |
| Charlson Score | C403 | 8849320 | Malignant tumor of the toe middle phalanx |
| Charlson Score | C403 | 8849324 | Patellar malignant tumor |
| Charlson Score | C403 | 8849327 | Malignant tumor of the toe distal phalanx |
| Charlson Score | C403 | 8849348 | Calcaneal malignant tumor |
| Charlson Score | C403 | 8849382 | Tarsal bone malignant tumor |
| Charlson Score | C403 | 8849384 | Malignant tumor of the foot scaphoid |
| Charlson Score | C403 | 8849405 | Metatarsal malignant tumor |
| Charlson Score | C410 | 1700005 | Skull malignant tumor |
| Charlson Score | C410 | 8835381 | Maxillary malignant tumor |
| Charlson Score | C410 | 8844240 | Maxillary malignant ameloblastoma |
| Charlson Score | C410 | 8846393 | Clivus chordoma |
| Charlson Score | C410 | 8846403 | Maxillary osteosarcoma |
| Charlson Score | C410 | 8846432 | Skull osteosarcoma |
| Charlson Score | C410 | 8846433 | Basal skull osteosarcoma |
| Charlson Score | C410 | 8848270 | Maxillary chondrosarcoma |
| Charlson Score | C410 | 8848294 | Basilar skull chondrosarcoma |
| Charlson Score | C410 | 8848304 | Paranasal sinus chondrosarcoma |
| Charlson Score | C410 | 8849267 | Malignant tumor of the facial bone |
| Charlson Score | C410 | 8849301 | Malignant tumor of the occipital bone |
| Charlson Score | C410 | 8849379 | Malignant tumor of the frontal bone |
| Charlson Score | C410 | 8849386 | Malignant tumor of the temporal bone |
| Charlson Score | C410 | 8849407 | Malignant tumor of the sphenoid bone |
| Charlson Score | C410 | 8849416 | Malignant tumor of the basilar skull |
| Charlson Score | C410 | 8849420 | Malignant tumor of the parietal bone |
| Charlson Score | C410 | 8849768 | Maxillary ameloblastic carcinoma |
| Charlson Score | C410 | 8849772 | Ghost cellular odontogenic carcinoma of the maxilla |
| Charlson Score | C410 | 8849774 | Primary intraosseous carcinoma of the maxilla |
| Charlson Score | C410 | 8849775 | Maxillary sclerosing odontogenic carcinoma |
| Charlson Score | C410 | 8849776 | Maxillary odontogenic sarcoma |
| Charlson Score | C410 | 8849781 | Clear cell odontogenic carcinoma of the maxilla |
| Charlson Score | C410 | 8849998 | Maxillary mesenchymal chondrosarcoma |
| Charlson Score | C410 | 8850001 | Maxillary periosteal osteosarcoma |
| Charlson Score | C410 | 8850002 | Low-grade central osteosarcoma of the maxilla |
| Charlson Score | C410 | 8850003 | Germ cell-type chondrosarcoma of the maxillary cartilage |

| **Category** | **ICD10** | **Diagnosis Code** | **Diagnosis Name** |
| --- | --- | --- | --- |
| Charlson Score | C410 | 8850007 | Maxillary juxtacortical osteosarcoma |
| Charlson Score | C411 | 1701003 | Malignant ameloblastoma |
| Charlson Score | C411 | 8844125 | Mandibular malignant ameloblastoma |
| Charlson Score | C411 | 8844128 | Mandibular malignant tumo |
| Charlson Score | C411 | 8846359 | Mandibular osteosarcoma |
| Charlson Score | C411 | 8848232 | Mandibular chondrosarcoma |
| Charlson Score | C411 | 8849716 | Mandibular ameloblastic carcinoma |
| Charlson Score | C411 | 8849720 | Ghost cellular odontogenic carcinoma of the mandible |
| Charlson Score | C411 | 8849722 | Primary intraosseous carcinoma of the mandible |
| Charlson Score | C411 | 8849723 | Mandibular sclerosing odontogenic carcinoma |
| Charlson Score | C411 | 8849724 | Mandibular odontogenic carcinosarcoma |
| Charlson Score | C411 | 8849725 | Mandibular odontogenic sarcoma |
| Charlson Score | C411 | 8849730 | Clear cell odontogenic carcinoma of the mandible |
| Charlson Score | C411 | 8849887 | Mandibular mesenchymal chondrosarcoma |
| Charlson Score | C411 | 8849890 | Mandibular periosteal osteosarcoma |
| Charlson Score | C411 | 8849891 | Low-grade central osteosarcoma of the mandible |
| Charlson Score | C411 | 8849892 | Germ cell-type chondrosarcoma of mandibular cartilage |
| Charlson Score | C411 | 8849896 | Mandibular juxtacortical osteosarcoma |
| Charlson Score | C412 | 8835199 | Axis chordoma |
| Charlson Score | C412 | 8840817 | Lumbar osteosarcoma |
| Charlson Score | C412 | 8846376 | Thoracic osteosarcoma |
| Charlson Score | C412 | 8846377 | Thoracic chordoma |
| Charlson Score | C412 | 8846382 | Cervical osteosarcoma |
| Charlson Score | C412 | 8846383 | Cervical chordoma |
| Charlson Score | C412 | 8846454 | Lumbar chordoma |
| Charlson Score | C412 | 8848238 | Thoracic chondrosarcoma |
| Charlson Score | C412 | 8848242 | Cervical chondrosarcoma |
| Charlson Score | C412 | 8848308 | Lumbar chondrosarcoma |
| Charlson Score | C412 | 8849270 | Thoracic malignant tumor |
| Charlson Score | C412 | 8849290 | Cervical malignant tumor |
| Charlson Score | C412 | 8849370 | Vertebral malignant tumor |
| Charlson Score | C412 | 8849483 | Lumbar malignant tumor |
| Charlson Score | C413 | 8846375 | Sternum osteosarcoma |
| Charlson Score | C413 | 8846389 | Clavicular osteosarcoma |
| Charlson Score | C413 | 8846455 | Rib osteosarcoma |
| Charlson Score | C413 | 8846987 | Rib Ewing’s sarcoma |
| Charlson Score | C413 | 8848237 | Sternum chondrosarcoma |
| Charlson Score | C413 | 8848252 | Clavicular chondrosarcoma |
| Charlson Score | C413 | 8848309 | Rib chondrosarcoma |
| Charlson Score | C413 | 8849269 | Sternum malignant tumor |
| Charlson Score | C413 | 8849307 | Clavicular malignant tumor |
| Charlson Score | C413 | 8849494 | Costochondral malignant tumor |
| Charlson Score | C413 | 8849496 | Rib malignant tumor |
| Charlson Score | C414 | 1958002 | Sacral chordoma |

| **Category** | **ICD10** | **Diagnosis Code** | **Diagnosis Name** |
| --- | --- | --- | --- |
| Charlson Score | C414 | 8836097 | Sacral chondrosarcoma |
| Charlson Score | C414 | 8837471 | Pubic chondrosarcoma |
| Charlson Score | C414 | 8846387 | Pelvic osteosarcoma |
| Charlson Score | C414 | 8846411 | Sacral osteosarcoma |
| Charlson Score | C414 | 8846423 | Pubic osteosarcoma |
| Charlson Score | C414 | 8846607 | Pelvic Ewing’s Sarcoma |
| Charlson Score | C414 | 8846739 | Sacral Ewing’s Sarcoma |
| Charlson Score | C414 | 8846868 | Iliac Ewing’s Sarcoma |
| Charlson Score | C414 | 8848249 | Pelvic chondrosarcoma |
| Charlson Score | C414 | 8848291 | Iliac chondrosarcoma |
| Charlson Score | C414 | 8849304 | Pelvic malignant tumor |
| Charlson Score | C414 | 8849310 | Ischial malignant tumor |
| Charlson Score | C414 | 8849372 | Sacral malignant tumor |
| Charlson Score | C414 | 8849401 | Pubic malignant tumor |
| Charlson Score | C414 | 8849409 | Iliac Malignant Tumor |
| Charlson Score | C414 | 8849446 | Coccygeal malignant tumor |
| Charlson Score | C419 | 1709002 | Ewing’s sarcoma |
| Charlson Score | C419 | 1709013 | Osteosarcoma |
| Charlson Score | C419 | 1709019 | Chondrosarcoma |
| Charlson Score | C419 | 1709023 | Juxtacortical osteosarcoma |
| Charlson Score | C419 | 1709026 | Primary bone tumor |
| Charlson Score | C419 | 1709034 | Malignant bone tumor |
| Charlson Score | C419 | 8833788 | Malignant fibrous histiocytoma of the bone |
| Charlson Score | C419 | 8833800 | Osteogenic sarcoma |
| Charlson Score | C419 | 8833817 | Fibrosarcoma of the bone |
| Charlson Score | C419 | 8833822 | Bone chondrosarcoma |
| Charlson Score | C419 | 8842724 | Periosteal osteosarcoma |
| Charlson Score | C419 | 8849705 | Malignant giant cell tumor of the bone |
| Charlson Score | C430 | 8842577 | Malignant melanoma of skin of lip |
| Charlson Score | C430 | 8846494 | Malignant melanoma of skin of lip |
| Charlson Score | C430 | 8846705 | Malignant melanoma of upper lip |
| Charlson Score | C431 | 8842562 | Malignant melanoma of eyelid |
| Charlson Score | C431 | 8846488 | Malignant melanoma of lower eyelid |
| Charlson Score | C431 | 8846699 | Malignant melanoma of upper eyelid |
| Charlson Score | C432 | 8845679 | Malignant melanoma of ear auricle |
| Charlson Score | C433 | 8842627 | Malignant melanoma of nasal cavity |
| Charlson Score | C433 | 8845641 | Malignant melanoma of mandible |
| Charlson Score | C433 | 8845644 | Facial malignant melanoma |
| Charlson Score | C433 | 8845651 | Malignant melanoma of cheek |
| Charlson Score | C433 | 8845689 | Frontal malignant melanoma |
| Charlson Score | C433 | 8845730 | Nasal malignant melanoma |
| Charlson Score | C433 | 8846689 | Pre-auricular malignant melanoma |
| Charlson Score | C433 | 8846907 | Malignant melanoma of nasal tip |
| Charlson Score | C433 | 8846912 | Malignant melanoma of nasal ridge |
| Charlson Score | C433 | 8846921 | Malignant melanoma of ala nasi |
| Charlson Score | C434 | 8845655 | Malignant melanoma of neck |
| Charlson Score | C434 | 8845661 | Malignant melanoma of nape |
| Charlson Score | C434 | 8845713 | Malignant melanoma of head |
| Charlson Score | C435 | 8830840 | Axillary melanoma |

| **Category** | **ICD10** | **Diagnosis Code** | **Diagnosis Name** |
| --- | --- | --- | --- |
| Charlson Score | C435 | 8842825 | Papillary malignant melanoma |
| Charlson Score | C435 | 8845652 | Malignant melanoma of chest |
| Charlson Score | C435 | 8845664 | Umbilical malignant melanoma |
| Charlson Score | C435 | 8845695 | Inguinal malignant melanoma |
| Charlson Score | C435 | 8845710 | Malignant melanoma of hip |
| Charlson Score | C435 | 8845717 | Malignant melanoma of back |
| Charlson Score | C435 | 8845733 | Malignant melanoma of abdomen |
| Charlson Score | C435 | 8845738 | Malignant melanoma of lumbar region |
| Charlson Score | C435 | 8846470 | Malignant melanoma of armpit |
| Charlson Score | C435 | 8846602 | Malignant melanoma of anus |
| Charlson Score | C435 | 8846734 | Sacral malignant melanoma |
| Charlson Score | C435 | 8846744 | Precordial malignant melanoma |
| Charlson Score | C435 | 8846763 | Malignant melanoma of lateral chest |
| Charlson Score | C436 | 8842596 | Malignant melanoma of upper limb |
| Charlson Score | C436 | 8845671 | Malignant melanoma of fingers |
| Charlson Score | C436 | 8845672 | Subungual malignant melanoma |
| Charlson Score | C436 | 8845673 | Malignant melanoma of palm |
| Charlson Score | C436 | 8845674 | Malignant melanoma of dorsum of the hand |
| Charlson Score | C436 | 8845683 | Malignant melanoma of upper arm |
| Charlson Score | C436 | 8845691 | Malignant melanoma of forearm joint |
| Charlson Score | C436 | 8845708 | Malignant melanoma of elbow |
| Charlson Score | C436 | 8846528 | Malignant melanoma of ring finger |
| Charlson Score | C436 | 8846583 | Malignant melanoma of shoulder |
| Charlson Score | C436 | 8846632 | Malignant melanoma of hand |
| Charlson Score | C436 | 8846648 | Malignant melanoma of little finger |
| Charlson Score | C436 | 8846680 | Malignant melanoma of index finger |
| Charlson Score | C436 | 8846856 | Malignant melanoma of middle finger |
| Charlson Score | C436 | 8846956 | Malignant melanoma of thumb |
| Charlson Score | C437 | 8842559 | Malignant melanoma of lower limb |
| Charlson Score | C437 | 8845628 | Malignant melanoma of toe |
| Charlson Score | C437 | 8845629 | Malignant melanoma of toenail |
| Charlson Score | C437 | 8845642 | Malignant melanoma of lower leg |
| Charlson Score | C437 | 8845668 | Malignant melanoma of knee |
| Charlson Score | C437 | 8845676 | Malignant melanoma of heel |
| Charlson Score | C437 | 8845692 | Malignant melanoma of sole |
| Charlson Score | C437 | 8845693 | Malignant melanoma of acrotarsium |
| Charlson Score | C437 | 8845694 | Malignant melanoma of foot |
| Charlson Score | C437 | 8845704 | Malignant melanoma of femur |
| Charlson Score | C437 | 8846807 | Malignant melanoma of the 2nd toe |
| Charlson Score | C437 | 8846813 | Malignant melanoma of the 3rd toe |
| Charlson Score | C437 | 8846819 | Malignant melanoma of the 4th toe |
| Charlson Score | C437 | 8846826 | Malignant melanoma of the 5th toe |
| Charlson Score | C437 | 8846957 | Malignant melanoma of hallux |
| Charlson Score | C438 | 1728001 | Subungual melanoma |
| Charlson Score | C438 | 8839361 | Malignant melanoma of skin |
| Charlson Score | C439 | 1729002 | Malignant melanoma |
| Charlson Score | C439 | 8846467 | Dysplastic nevus syndrome |
| Charlson Score | C439 | 8848313 | BRAF gene mutation positive malignant melanoma |

| **Category** | **ICD10** | **Diagnosis Code** | **Diagnosis Name** |
| --- | --- | --- | --- |
| Charlson Score | C450 | 1639004 | Pleural mesothelioma |
| Charlson Score | C450 | 1639005 | Pleural mesothelioma malignant localised |
| Charlson Score | C450 | 8842659 | Peritoneal malignant mesothelioma |
| Charlson Score | C451 | 8839764 | Peritoneal mesothelioma |
| Charlson Score | C451 | 8842664 | Peritoneal malignant mesothelioma |
| Charlson Score | C452 | 8835141 | Pericardial mesothelioma |
| Charlson Score | C452 | 8842660 | Pericardial malignant mesothelioma |
| Charlson Score | C459 | 2399009 | Mesothelioma |
| Charlson Score | C459 | 2399014 | Malignant mesothelioma |
| Charlson Score | C459 | 8833282 | Localized malignant mesothelioma |
| Charlson Score | C459 | 8839547 | Diffuse mesothelioma |
| Charlson Score | C469 | 1739001 | Kaposi sarcoma |
| Charlson Score | C470 | 8845898 | Malignant peripheral nerve sheath tumor of neck |
| Charlson Score | C471 | 8845905 | Malignant peripheral nerve sheath tumor of shoulder |
| Charlson Score | C471 | 8845959 | Malignant peripheral schwannoma of upper arm |
| Charlson Score | C472 | 8846001 | Malignant peripheral nerve sheath tumor of femur |
| Charlson Score | C472 | 8848537 | Malignant peripheral nerve sheath tumor of knee |
| Charlson Score | C473 | 8845855 | Malignant peripheral nerve sheath tumor of axilla |
| Charlson Score | C473 | 8845890 | Malignant peripheral nerve sheath tumor of chest wall |
| Charlson Score | C474 | 8848664 | Malignant peripheral nerve sheath tumor of abdomen |
| Charlson Score | C475 | 8845921 | Malignant peripheral nerve sheath tumor of  retroperitoneum |
| Charlson Score | C475 | 8846023 | Malignant peripheral nerve sheath tumor of hip |
| Charlson Score | C476 | 8848653 | Back malignant peripheral nerve sheath tumor |
| Charlson Score | C479 | 1718003 | Neurofibrosarcomas |
| Charlson Score | C479 | 1719007 | Nerve sheath malignant tumor |
| Charlson Score | C479 | 8840254 | Malignant neoplasm of peripheral nerve |
| Charlson Score | C479 | 8845846 | Malignant peripheral nerve sheath tumor |
| Charlson Score | C480 | 8833668 | Retroperitoneal malignant neoplasm |
| Charlson Score | C480 | 8844956 | Giant retroperitoneal liposarcoma |
| Charlson Score | C480 | 8844967 | Retroperitoneal liposarcoma |
| Charlson Score | C480 | 8844989 | Perirenal liposarcoma |
| Charlson Score | C480 | 8845920 | Malignant fibrous histiocytoma of retroperitoneum |
| Charlson Score | C480 | 8845922 | Retroperitoneal rhabdomyosarcoma |
| Charlson Score | C480 | 8845923 | Retroperitoneal hemangiosarcoma |
| Charlson Score | C480 | 8845924 | Retroperitoneal fibrosarcoma |
| Charlson Score | C480 | 8845925 | Retroperitoneal leiomyosarcoma |
| Charlson Score | C480 | 8847336 | Retroperitoneal germ cell tumor |
| Charlson Score | C480 | 8848047 | Retroperitoneal neuroblastoma |
| Charlson Score | C481 | 8837688 | Malignant mesenteric neoplasm |
| Charlson Score | C481 | 8837699 | Mesenteric sarcoma |

| **Category** | **ICD10** | **Diagnosis Code** | **Diagnosis Name** |
| --- | --- | --- | --- |
| Charlson Score | C481 | 8845012 | Omental liposarcoma |
| Charlson Score | C481 | 8845015 | Mesenteric liposarcoma |
| Charlson Score | C481 | 8846016 | Mesenteric leiomyosarcoma |
| Charlson Score | C481 | 8847884 | Omental gastrointestinal stromal tumor |
| Charlson Score | C481 | 8847885 | Mesenteric gastrointestinal stromal tumor |
| Charlson Score | C481 | 8848131 | Malignant mesocolon neoplasm |
| Charlson Score | C481 | 8848153 | Malignant omental neoplasm |
| Charlson Score | C482 | 8839761 | Malignant peritoneal neoplasm |
| Charlson Score | C482 | 8839762 | Peritoneal carcinoma |
| Charlson Score | C490 | 1710002 | Malignant neoplasm of soft tissue of neck |
| Charlson Score | C490 | 8833011 | Sarcoma of neck |
| Charlson Score | C490 | 8835189 | Sarcoma of ear |
| Charlson Score | C490 | 8844960 | Liposarcoma of ncek |
| Charlson Score | C490 | 8845022 | Liposarcoma of head |
| Charlson Score | C490 | 8845270 | Malignant soft tissue tumor of neck |
| Charlson Score | C490 | 8845859 | Rhabdomyosarcoma of jaw |
| Charlson Score | C490 | 8845882 | Facial rhabdomyosarcoma |
| Charlson Score | C490 | 8845887 | Buccal rhabdomyosarcoma |
| Charlson Score | C490 | 8845888 | Buccal hemangiosarcoma |
| Charlson Score | C490 | 8845897 | Malignant fibrous histiocytoma of neck |
| Charlson Score | C490 | 8845899 | Rhabdomyosarcoma of ncek |
| Charlson Score | C490 | 8845900 | Synovial sarcoma of ncek |
| Charlson Score | C490 | 8845901 | Hemangiosarcoma of neck |
| Charlson Score | C490 | 8846030 | Malignant fibrous histiocytoma of head |
| Charlson Score | C490 | 8846031 | Rhabdomyosarcoma of head |
| Charlson Score | C490 | 8846032 | Synovial sarcoma of head |
| Charlson Score | C490 | 8846033 | Hemangiosarcoma of head |
| Charlson Score | C491 | 8837917 | Malignant soft tissue tumor of hand |
| Charlson Score | C491 | 8844988 | Brachial liposarcoma |
| Charlson Score | C491 | 8845326 | Malignant soft tissue tumor of upper arm |
| Charlson Score | C491 | 8845359 | Malignant soft tissue tumor of forearm |
| Charlson Score | C491 | 8845904 | Malignant fibrous histiocytoma of shoulder |
| Charlson Score | C491 | 8845906 | Rhabdomyosarcoma of shoulder |
| Charlson Score | C491 | 8845907 | Synovial sarcoma of shoulder |
| Charlson Score | C491 | 8845908 | Fibrosarcoma of shoulder |
| Charlson Score | C491 | 8845909 | Clear cell sarcoma of shoulder |
| Charlson Score | C491 | 8845910 | Alveolar soft part sarcoma of shoulder |
| Charlson Score | C491 | 8845939 | Synovial sarcoma of wrist |
| Charlson Score | C491 | 8845942 | Malignant fibrous histiocytomas of hand |
| Charlson Score | C491 | 8845943 | Rhabdomyosarcoma of hand |
| Charlson Score | C491 | 8845944 | Synovial sarcoma of hand |
| Charlson Score | C491 | 8845945 | Clear cell sarcoma of hand |
| Charlson Score | C491 | 8845946 | Epithelioid sarcoma of hand |
| Charlson Score | C491 | 8845958 | Malignant fibrous histiocytoma of upper arm |
| Charlson Score | C491 | 8845960 | Brachial rhabdomyosarcoma |
| Charlson Score | C491 | 8845961 | Brachial synovial sarcoma |
| Charlson Score | C491 | 8845962 | Brachial fibrosarcoma |
| Charlson Score | C491 | 8845963 | Clear cell sarcoma of upper arm |
| Charlson Score | C491 | 8845964 | Alveolar soft part sarcoma of upper arm |

| **Category** | **ICD10** | **Diagnosis Code** | **Diagnosis Name** |
| --- | --- | --- | --- |
| Charlson Score | C491 | 8845965 | Epithelioid sarcoma of upper arm |
| Charlson Score | C491 | 8845981 | Malignant fibrous histiocytoma of forearm |
| Charlson Score | C491 | 8845982 | Rhabdomyosarcoma of forearm |
| Charlson Score | C491 | 8845983 | Synovial sarcoma of forearm |
| Charlson Score | C491 | 8845984 | Fibrosarcoma of forearm |
| Charlson Score | C491 | 8845985 | Alveolar soft part sarcoma of forearm |
| Charlson Score | C491 | 8845986 | Epithelioid sarcoma of forearm |
| Charlson Score | C491 | 8846013 | Synovial sarcoma of elbow |
| Charlson Score | C491 | 8846014 | Fibrosarcoma of elbow |
| Charlson Score | C491 | 8846015 | Epithelioid sarcoma of elbow |
| Charlson Score | C491 | 8848506 | Malignant soft tissue tumor of shoulder |
| Charlson Score | C491 | 8848567 | Brachial leiomyosarcoma |
| Charlson Score | C491 | 8848597 | Leiomyosarcoma of forearm |
| Charlson Score | C492 | 8837348 | Femoral liposarcoma |
| Charlson Score | C492 | 8837351 | Femoral fibrosarcoma |
| Charlson Score | C492 | 8844941 | Liposarcoma of lower leg |
| Charlson Score | C492 | 8845209 | Malignant soft tissue tumor of foot |
| Charlson Score | C492 | 8845226 | Malignant soft tissue tumor of lower leg |
| Charlson Score | C492 | 8845406 | Malignant soft tissue tumor of femur |
| Charlson Score | C492 | 8845865 | Malignant fibrous histiocytoma of lower leg |
| Charlson Score | C492 | 8845866 | Rhabdomyosarcoma of lower leg |
| Charlson Score | C492 | 8845867 | Synovial sarcoma of lower leg |
| Charlson Score | C492 | 8845868 | Fibrosarcoma of lower leg |
| Charlson Score | C492 | 8845869 | Clear cell sarcoma of lower leg |
| Charlson Score | C492 | 8845870 | Leiomyosarcoma of lower leg |
| Charlson Score | C492 | 8845871 | Alveolar soft part sarcoma of lower leg |
| Charlson Score | C492 | 8845872 | Epithelioid sarcoma of lower leg |
| Charlson Score | C492 | 8845926 | Synovial sarcoma of hip joint |
| Charlson Score | C492 | 8845935 | Synovial sarcoma of knee |
| Charlson Score | C492 | 8845936 | Malignant fibrous histiocytoma of knee |
| Charlson Score | C492 | 8845937 | Clear cell sarcoma of knee |
| Charlson Score | C492 | 8845938 | Alveolar soft part sarcoma of knee |
| Charlson Score | C492 | 8845990 | Synovial sarcoma of ankle |
| Charlson Score | C492 | 8845991 | Rhabdomyosarcoma of foot |
| Charlson Score | C492 | 8845992 | Synovial sarcoma of foot |
| Charlson Score | C492 | 8845993 | Clear cell sarcoma of foot |
| Charlson Score | C492 | 8845994 | Epithelioid sarcoma of foot |
| Charlson Score | C492 | 8846000 | Malignant fibrous histiocytoma of femur |
| Charlson Score | C492 | 8846002 | Rhabdomyosarcoma of femur |
| Charlson Score | C492 | 8846003 | Synovial sarcoma of femur |
| Charlson Score | C492 | 8846004 | Hemangiosarcoma of femur |
| Charlson Score | C492 | 8846005 | Leiomyosarcoma of femur |
| Charlson Score | C492 | 8846006 | Alveolar soft part sarcoma of femur |
| Charlson Score | C492 | 8846007 | Epithelioid sarcoma of femur |
| Charlson Score | C492 | 8848536 | Malignant soft tissue tumor of knee |
| Charlson Score | C493 | 8844954 | Liposarcoma of chest wall |
| Charlson Score | C493 | 8844965 | Scapular liposarcoma |
| Charlson Score | C493 | 8845258 | Malignant soft tissue tumor of chest |
| Charlson Score | C493 | 8845889 | Malignant fibrous histiocytoma of chest wall |

| **Category** | **ICD10** | **Diagnosis Code** | **Diagnosis Name** |
| --- | --- | --- | --- |
| Charlson Score | C493 | 8845891 | Rhabdomyosarcoma of chest wall |
| Charlson Score | C493 | 8845892 | Hemangiosarcoma of cheat wall |
| Charlson Score | C493 | 8845893 | Fibrosarcoma of chest wall |
| Charlson Score | C493 | 8845894 | Clear cell sarcoma of chest wall |
| Charlson Score | C493 | 8848488 | Leiomyosarcoma of chest |
| Charlson Score | C494 | 8845033 | Abdominal liposarcoma |
| Charlson Score | C494 | 8845485 | Malignant soft tissue tumor of abdomen |
| Charlson Score | C494 | 8846048 | Abdominal leiomyosarcoma |
| Charlson Score | C494 | 8846049 | Malignant fibrous histiocytoma of abdominal wall |
| Charlson Score | C494 | 8846050 | Rhabdomyosarcoma of abdominal wall |
| Charlson Score | C494 | 8846051 | Fibrosarcoma of abdominal wall |
| Charlson Score | C495 | 8844972 | Ischiorectal liposarcoma |
| Charlson Score | C495 | 8845004 | Inguinal liposarcoma |
| Charlson Score | C495 | 8845282 | Pelvic malignant soft tissue tumor |
| Charlson Score | C495 | 8845283 | Malignant soft tissue tumor of pelvis |
| Charlson Score | C495 | 8845444 | Malignant soft tissue tumor of hip |
| Charlson Score | C495 | 8845995 | Malignant fibrous histiocytoma of inguinal hernia |
| Charlson Score | C495 | 8845996 | Inguinal rhabdomyosarcoma |
| Charlson Score | C495 | 8845997 | Synovial sarcoma of inguinal hernia |
| Charlson Score | C495 | 8846022 | Malignant fibrous histiocytoma of hip |
| Charlson Score | C495 | 8846024 | Rhabdomyosarcoma of hip |
| Charlson Score | C495 | 8846025 | Synovial sarcoma of hip |
| Charlson Score | C495 | 8846026 | Hemangiosarcoma of hip |
| Charlson Score | C495 | 8846027 | Fibrosarcoma of hip |
| Charlson Score | C495 | 8846028 | Leiomyosarcoma of hip |
| Charlson Score | C495 | 8846029 | Alveolar soft part sarcoma of hip |
| Charlson Score | C495 | 8850262 | Malignant soft tissue tumor of inguinal hernia |
| Charlson Score | C496 | 8845028 | Liposarcoma of back |
| Charlson Score | C496 | 8845040 | Liposarcoma of lion |
| Charlson Score | C496 | 8845468 | Malignant soft tissue tumor of back |
| Charlson Score | C496 | 8846039 | Malignant fibrous histiocytoma of back |
| Charlson Score | C496 | 8846040 | Rhabdomyosarcoma of back |
| Charlson Score | C496 | 8846066 | Malignant fibrous histiocytoma of loin |
| Charlson Score | C496 | 8848654 | Leiomyosarcoma of back |
| Charlson Score | C499 | 1719010 | Rhabdomyosarcomas |
| Charlson Score | C499 | 1719012 | Synovial sarcoma |
| Charlson Score | C499 | 1719013 | Hemangiosarcoma |
| Charlson Score | C499 | 1719018 | Liposarcoma |
| Charlson Score | C499 | 1719025 | Fibrosarcomas malignant |
| Charlson Score | C499 | 1719033 | Leiomyosarcomas |
| Charlson Score | C499 | 1719035 | Alveolar soft part sarcomas |
| Charlson Score | C499 | 1719043 | Synovioma |
| Charlson Score | C499 | 1719052 | Mesenchymomas malignant |
| Charlson Score | C499 | 2001018 | Lymphangiosarcomas |
| Charlson Score | C499 | 2028045 | Malignant fibrous histiocytomas |
| Charlson Score | C499 | 2028046 | Malignant granular cell tumor |
| Charlson Score | C499 | 2028047 | Soft-tissue malignant giant cell tumor |

| **Category** | **ICD10** | **Diagnosis Code** | **Diagnosis Name** |
| --- | --- | --- | --- |
| Charlson Score | C499 | 2280108 | Malignant hemangiopericytoma |
| Charlson Score | C499 | 8830211 | Malignant glomus tumor |
| Charlson Score | C499 | 8836066 | Fibrous liposarcoma |
| Charlson Score | C499 | 8836884 | Embryonal sarcoma |
| Charlson Score | C499 | 8838371 | Malignant soft tissue tumor of neck |
| Charlson Score | C499 | 8838381 | Sarcoma |
| Charlson Score | C499 | 8840149 | Spindle cell sarcoma |
| Charlson Score | C499 | 8845999 | Clear cell sarcoma |
| Charlson Score | C499 | 8846068 | Epithelioid sarcomas |
| Charlson Score | C499 | 8848150 | Desmoplastic small round cell tumor |
| Charlson Score | C499 | 8848447 | Malignant soft tissue tumor |
| Charlson Score | C499 | 8849841 | Undifferentiated sarcoma |
| Charlson Score | C500 | 8838489 | Mammary Paget’s disease |
| Charlson Score | C500 | 8845450 | Papillary breast cancer |
| Charlson Score | C500 | 8845452 | Areolar breast cancer |
| Charlson Score | C501 | 8838483 | Malignant neoplasm of central portion of breast |
| Charlson Score | C502 | 8838476 | Malignant neoplasm of upper-inner quadrant of breast |
| Charlson Score | C503 | 8838465 | Malignant neoplasm of lower-inner quadrant of breast |
| Charlson Score | C504 | 8838475 | Malignant neoplasm of upper-outer quadrant of breast |
| Charlson Score | C505 | 8838464 | Malignant neoplasm of lower-outer quadrant of breast |
| Charlson Score | C506 | 8845449 | Malignant neoplasm of axillary tail of breast |
| Charlson Score | C506 | 8848843 | Axillary breast cancer |
| Charlson Score | C508 | 8845451 | Malignant neoplasm of overlapping lesion of breast |
| Charlson Score | C508 | 8848690 | Ectopic breast cancer |
| Charlson Score | C509 | 1749004 | Postoperative breast cancer |
| Charlson Score | C509 | 1749008 | Carcinoma breast |
| Charlson Score | C509 | 1749009 | Recurrent breast cancer |
| Charlson Score | C509 | 1749011 | Malignant neoplasm of mammary gland |
| Charlson Score | C509 | 1749015 | Malignant breast neoplasm |
| Charlson Score | C509 | 1749017 | Breast sarcoma |
| Charlson Score | C509 | 8830917 | Inflammatory breast cancer |
| Charlson Score | C509 | 8842665 | Malignant phyllodes tumor |
| Charlson Score | C509 | 8842759 | Advanced breast cancer |
| Charlson Score | C509 | 8844799 | HER2-overexpression breast cancer |
| Charlson Score | C509 | 8845025 | Breast liposarcoma |
| Charlson Score | C509 | 8848646 | Hemangiosarcoma of breast |
| Charlson Score | C509 | 8848647 | Fibrosarcoma of breast |
| Charlson Score | C509 | 8848722 | Invasive ductal breast cancer |
| Charlson Score | C509 | 8848743 | Multiple breast cancer |
| Charlson Score | C509 | 8848773 | Papillary ductal carcinoma |
| Charlson Score | C509 | 8849183 | Mammary scirrhous carcinoma |
| Charlson Score | C509 | 8849184 | Solid ductal breast carcinoma |
| Charlson Score | C509 | 8849699 | HER2 positive breast cancer |
| Charlson Score | C509 | 8849815 | Locally recurrent breast cancer |

| **Category** | **ICD10** | **Diagnosis Code** | **Diagnosis Name** |
| --- | --- | --- | --- |
| Charlson Score | C509 | 8849816 | Chest wall recurrence after breast cancer |
| Charlson Score | C510 | 1841002 | Labia majora cancer |
| Charlson Score | C511 | 1842002 | Labia minora cancer |
| Charlson Score | C512 | 1843002 | Clitoris cancer |
| Charlson Score | C519 | 1844003 | Vulvar cancer |
| Charlson Score | C519 | 1844007 | Malignant vulvar melanoma |
| Charlson Score | C519 | 8831684 | Malignant vulvar neoplasm |
| Charlson Score | C519 | 8842695 | Paget’s disease of the vulva |
| Charlson Score | C519 | 8848036 | Vulval squamous cell carcinoma |
| Charlson Score | C519 | 8848809 | Vulval apocrine carcinoma |
| Charlson Score | C52 | 8837487 | Malignant neoplasm of vagina |
| Charlson Score | C52 | 8837496 | Vaginal cancer |
| Charlson Score | C530 | 1800003 | Uterine cervix cancer |
| Charlson Score | C531 | 8834243 | Malignant neoplasm of exocervix |
| Charlson Score | C538 | 8834242 | Cervical stump cancer |
| Charlson Score | C538 | 8848883 | Cervical overlapping lesion cancer |
| Charlson Score | C539 | 1809004 | Cervical cancer |
| Charlson Score | C539 | 8842739 | Microinvasive cervix cancer |
| Charlson Score | C539 | 8844722 | Cervical adenocarcinoma |
| Charlson Score | C540 | 8847757 | Isthmus uteri cancer |
| Charlson Score | C541 | 1799007 | Endometrial stromal sarcoma |
| Charlson Score | C541 | 1820005 | Endometrial carcinoma |
| Charlson Score | C541 | 8848717 | Endometrioid adenocarcinoma |
| Charlson Score | C542 | 1799005 | Uterine sarcoma |
| Charlson Score | C542 | 1799006 | Uterine leiomyosarcoma |
| Charlson Score | C543 | 8847758 | Uterine fundus cancer |
| Charlson Score | C549 | 1799008 | Malignant mixed müllerian tumor |
| Charlson Score | C549 | 1820002 | Endometrial cancer |
| Charlson Score | C549 | 1820003 | Recurrent endometrial cancer |
| Charlson Score | C549 | 8846283 | Uterus carcinosarcoma |
| Charlson Score | C549 | 8848716 | Malignant uterine neoplasm |
| Charlson Score | C55 | 1799003 | Uterine carcinoma |
| Charlson Score | C55 | 1799004 | Recurrent uterine cancer |
| Charlson Score | C56 | 1830003 | Malignant ovarian neoplasm |
| Charlson Score | C56 | 1830005 | Ovarian carcinoma |
| Charlson Score | C56 | 1830008 | Ovarian sarcoma |
| Charlson Score | C56 | 1830009 | Ovarian dysgerminoma |
| Charlson Score | C56 | 1830014 | Ovarian choriocarcinoma |
| Charlson Score | C56 | 1830027 | Ovarian embryonal carcinoma |
| Charlson Score | C56 | 8838667 | Mucinous cystadenocarcinoma |
| Charlson Score | C56 | 8840926 | Cystic carcinoma of ovary |
| Charlson Score | C56 | 8846347 | Ovarian carcinosarcoma |
| Charlson Score | C56 | 8846984 | Ovarian carcinoid |
| Charlson Score | C56 | 8847437 | Ovarian germ cell tumor |
| Charlson Score | C56 | 8847438 | Ovarian yolk sac tumor |
| Charlson Score | C56 | 8848712 | Recurrent ovarian cancer |
| Charlson Score | C56 | 8848765 | Poorly differentiated Sertoli–Leydig cell tumors |
| Charlson Score | C56 | 8848795 | Ovarian serous adenocarcinoma |
| Charlson Score | C56 | 8848796 | Ovarian small cell carcinoma |

| **Category** | **ICD10** | **Diagnosis Code** | **Diagnosis Name** |
| --- | --- | --- | --- |
| Charlson Score | C56 | 8848799 | Ovarian squamous cell carcinoma |
| Charlson Score | C56 | 8848800 | Immature ovarian teratoma |
| Charlson Score | C56 | 8848801 | Ovarian clear cell adenocarcinoma |
| Charlson Score | C56 | 8848802 | Ovarian endometrioid adenocarcinoma |
| Charlson Score | C56 | 8849004 | Ovarian mature cystic teratoma, malignant transformation |
| Charlson Score | C56 | 8849005 | Ovarian mucinous adenocarcinoma |
| Charlson Score | C570 | 1832003 | Fallopian tube cancer |
| Charlson Score | C579 | 8835564 | Female genital cancer |
| Charlson Score | C58 | 1810001 | Choriocarcinoma |
| Charlson Score | C58 | 8848741 | Placental choriocarcinoma |
| Charlson Score | C600 | 8830639 | Prepuce cancer |
| Charlson Score | C601 | 8830620 | Glans penis cancer |
| Charlson Score | C602 | 8830632 | Malignant neoplasm of body of penis |
| Charlson Score | C609 | 1874002 | Penis carcinoma |
| Charlson Score | C609 | 8830633 | Penile sarcoma |
| Charlson Score | C609 | 8845636 | Paget’s disease of penis |
| Charlson Score | C609 | 8848027 | Malignant penile melanoma |
| Charlson Score | C609 | 8848028 | Penile squamous cell carcinoma |
| Charlson Score | C609 | 8848807 | Penile apocrine carcinoma |
| Charlson Score | C61 | 1859003 | Prostate cancer |
| Charlson Score | C61 | 1859004 | Prostate sarcoma |
| Charlson Score | C61 | 8842789 | Neuroendocrine carcinoma of prostate |
| Charlson Score | C61 | 8845979 | Rhabdomyosarcoma of prostate |
| Charlson Score | C61 | 8845980 | Prostate small cell cancer |
| Charlson Score | C61 | 8848040 | Castration-resistant prostate cancer |
| Charlson Score | C61 | 8848043 | Localized prostate cancer |
| Charlson Score | C61 | 8848066 | Progressive prostate cancer |
| Charlson Score | C61 | 8848074 | Recurrent prostate cancer |
| Charlson Score | C620 | 8842661 | Malignant neoplasm of undescended testis |
| Charlson Score | C621 | 8840102 | Hormone-secreting testicular tumor |
| Charlson Score | C629 | 1869005 | Malignant testicular neoplasm |
| Charlson Score | C629 | 1869012 | Spermatoma |
| Charlson Score | C629 | 1869014 | Testicular cancer |
| Charlson Score | C629 | 8835874 | Seminoma |
| Charlson Score | C629 | 8835893 | Testis teratocarcinoma |
| Charlson Score | C629 | 8835894 | Teratoma of testis |
| Charlson Score | C629 | 8835900 | Testicular choriocarcinoma |
| Charlson Score | C629 | 8835911 | Testicular embryonal carcinoma |
| Charlson Score | C629 | 8835914 | Testicular sarcoma |
| Charlson Score | C629 | 8835922 | Testicular tumor |
| Charlson Score | C629 | 8836910 | Embryonic testicular tumor |
| Charlson Score | C629 | 8845972 | Rhabdomyosarcoma of testis |
| Charlson Score | C629 | 8847379 | Testicular germ cell tumor |
| Charlson Score | C629 | 8847380 | Testicular yolk sac tumor |
| Charlson Score | C629 | 8848924 | Testicular seminoma |
| Charlson Score | C629 | 8848925 | Spermatocytic seminoma |
| Charlson Score | C630 | 8835901 | Epididymal cancer |
| Charlson Score | C631 | 8835850 | Spermatic cord sarcoma |

| **Category** | **ICD10** | **Diagnosis Code** | **Diagnosis Name** |
| --- | --- | --- | --- |
| Charlson Score | C631 | 8844996 | Spermatic cord liposarcoma |
| Charlson Score | C632 | 8830683 | Scrotal cancer |
| Charlson Score | C632 | 8844935 | Intrascrotal liposarcoma |
| Charlson Score | C632 | 8845637 | Paget’s disease of vulva |
| Charlson Score | C632 | 8848029 | Scrotal malignant melanoma |
| Charlson Score | C632 | 8848030 | Scrotal squamous cell carcinoma |
| Charlson Score | C637 | 8835947 | Seminal cancer |
| Charlson Score | C637 | 8835953 | Seminal sarcoma |
| Charlson Score | C639 | 8837452 | Male genital cancer |
| Charlson Score | C64 | 1890009 | Renal carcinoma |
| Charlson Score | C64 | 1890010 | Renal cell cancer |
| Charlson Score | C64 | 1890014 | Malignant renal neoplasm |
| Charlson Score | C64 | 8830766 | Wilms’ tumor |
| Charlson Score | C64 | 8835635 | Renal sarcoma |
| Charlson Score | C64 | 8846721 | Renal carcinoid |
| Charlson Score | C64 | 8847538 | Renal cell carcinoma associated with acquired cystic kidney |
| Charlson Score | C64 | 8848274 | Renal clear cell sarcoma |
| Charlson Score | C64 | 8848275 | Rhabdoid tumor of kidney |
| Charlson Score | C64 | 8848359 | Nephroblastoma |
| Charlson Score | C65 | 1891003 | Renal pelvic cancer |
| Charlson Score | C65 | 1891005 | Papillary carcinoma of renal pelvis |
| Charlson Score | C65 | 8845335 | Renal pelvic adenocarcinoma |
| Charlson Score | C65 | 8845336 | Urothelial cancer of renal pelvis |
| Charlson Score | C65 | 8845337 | Squamous cell carcinoma of renal pelvis |
| Charlson Score | C66 | 1892003 | Ureteral cancer |
| Charlson Score | C66 | 8845454 | Urethral urothelial carcinoma |
| Charlson Score | C670 | 8840134 | Malignant neoplasm of trigone of bladder |
| Charlson Score | C671 | 8840121 | Malignant neoplasm of dome of bladder |
| Charlson Score | C672 | 8840138 | Malignant neoplasm of lateral wall of bladder |
| Charlson Score | C673 | 8840137 | Malignant neoplasm of anterior wall of bladder |
| Charlson Score | C674 | 8840132 | Malignant neoplasm of posterior wall of bladder |
| Charlson Score | C675 | 8840128 | Malignant neoplasm of bladder neck |
| Charlson Score | C676 | 8838508 | Malignant neoplasm of ureteric orifice |
| Charlson Score | C677 | 1887002 | Urachal cancer |
| Charlson Score | C679 | 1889005 | Urinary bladder carcinoma |
| Charlson Score | C679 | 8840140 | Urinary bladder sarcoma |
| Charlson Score | C679 | 8845493 | Bladder urothelial carcinoma |
| Charlson Score | C679 | 8845494 | Bladder squamous cell carcinoma |
| Charlson Score | C680 | 1893003 | Urethral cancer |
| Charlson Score | C680 | 8845456 | Urethral urothelial carcinoma |
| Charlson Score | C681 | 8838552 | Malignant neoplasm of paraurethral gland |
| Charlson Score | C690 | 8833186 | Malignant neoplasm of conjunctiva |
| Charlson Score | C691 | 8831189 | Malignant neoplasm of cornea |
| Charlson Score | C692 | 1905003 | Retinoblastoma |
| Charlson Score | C692 | 1905005 | Retinal glioma |
| Charlson Score | C693 | 1906004 | Choroidal malignant melanoma |
| Charlson Score | C694 | 8842847 | Uveal malignant melanoma |
| Charlson Score | C694 | 8842867 | Malignant neoplasm of ciliary body |

| **Category** | **ICD10** | **Diagnosis Code** | **Diagnosis Name** |
| --- | --- | --- | --- |
| Charlson Score | C695 | 8850324 | Nasolacrimal duct carcinoma |
| Charlson Score | C695 | 8850361 | Malignant neoplasm of lacrimal sac |
| Charlson Score | C696 | 1901002 | Malignant orbital neoplasm |
| Charlson Score | C696 | 8842697 | Orbital neuroblastoma |
| Charlson Score | C696 | 8845879 | Rhabdomyosarcoma of orbit |
| Charlson Score | C700 | 1921002 | Malignant supratentorial and infratentorial meningioma |
| Charlson Score | C700 | 1921003 | Supratentorial and infratentorial glioma |
| Charlson Score | C700 | 1921019 | Sarcomatous meningioma |
| Charlson Score | C700 | 8830221 | Cerebral malignant meningioma |
| Charlson Score | C700 | 8842663 | Malignant cerebroventricular meningioma |
| Charlson Score | C700 | 8848452 | Malignant tuberculum sellae meningioma |
| Charlson Score | C700 | 8848467 | Malignant fornix meningioma |
| Charlson Score | C700 | 8848547 | Cerebellopontine angle malignant meningioma |
| Charlson Score | C700 | 8848548 | Cerebellar tentorial hemangiopericytoma |
| Charlson Score | C700 | 8848609 | Falx cerebri malignant meningioma |
| Charlson Score | C700 | 8848610 | Falx cerebri hemangiopericytoma |
| Charlson Score | C700 | 8848617 | Sphenoid wing malignant meningioma |
| Charlson Score | C700 | 8848672 | Parasagittal malignant meningioma |
| Charlson Score | C701 | 1923001 | Malignant spinal meningioma |
| Charlson Score | C709 | 1921005 | Malignant meningiomas |
| Charlson Score | C710 | 1910001 | Malignant brain neoplasm |
| Charlson Score | C710 | 1910003 | Deep cerebral glioma |
| Charlson Score | C710 | 8847615 | Hypothalamic astrocytoma |
| Charlson Score | C710 | 8847616 | Thalamic astrocytoma |
| Charlson Score | C710 | 8848619 | Tentorial hemangiopericytoma |
| Charlson Score | C710 | 8848745 | Cerebral oligodendroglioma |
| Charlson Score | C710 | 8850191 | Thalamic glioblastoma |
| Charlson Score | C711 | 8836571 | Malignant neoplasm of frontal lobe |
| Charlson Score | C711 | 8846752 | Frontal lobe glioblastoma |
| Charlson Score | C711 | 8846753 | Frontal lobe glioma |
| Charlson Score | C711 | 8847648 | Frontal lobe astrocytoma |
| Charlson Score | C711 | 8847649 | Frontal lobe anaplastic astrocytoma |
| Charlson Score | C711 | 8848733 | Frontal lobe anaplastic oligodendroglioma |
| Charlson Score | C711 | 8848734 | Frontal lobe oligodendroglioma |
| Charlson Score | C712 | 8836758 | Malignant neoplasm of temporal lobe |
| Charlson Score | C712 | 8842793 | Temporal lobe glioblastoma |
| Charlson Score | C712 | 8846774 | Temporal lobe glioma |
| Charlson Score | C712 | 8847654 | Temporal lobe astrocytoma |
| Charlson Score | C712 | 8847655 | Temporal lobe anaplastic astrocytoma |
| Charlson Score | C712 | 8847656 | Temporal lobe pilocytic astrocytoma |
| Charlson Score | C712 | 8848735 | Temporal lobe anaplastic oligodendroglioma |
| Charlson Score | C712 | 8848736 | Temporal lobe oligodendroglioma |
| Charlson Score | C713 | 8838059 | Malignant neoplasm of parietal lobe |
| Charlson Score | C713 | 8846880 | Parietal lobe glioblastoma |
| Charlson Score | C713 | 8846881 | Parietal lobe glioma |
| Charlson Score | C713 | 8847667 | Parietal lobe astrocytoma |
| Charlson Score | C713 | 8848766 | Parietal lobe anaplastic oligodendroglioma |
| Charlson Score | C713 | 8848767 | Parietal lobe oligodendroglioma |

| **Category** | **ICD10** | **Diagnosis Code** | **Diagnosis Name** |
| --- | --- | --- | --- |
| Charlson Score | C714 | 8833643 | Malignant neoplasm of occipital lobe |
| Charlson Score | C714 | 8846596 | Occipital lobe glioblastoma |
| Charlson Score | C714 | 8846597 | Occipital lobe glioma |
| Charlson Score | C715 | 1915008 | Ependymoma |
| Charlson Score | C715 | 8838712 | Malignant neoplasm of cerebral ventricle |
| Charlson Score | C716 | 1916004 | Cerebellar medulloblastoma |
| Charlson Score | C716 | 1916006 | Cerebellar astrocytoma |
| Charlson Score | C716 | 1919020 | Medulloblastoma |
| Charlson Score | C716 | 8846659 | Cerebellar glioblastoma |
| Charlson Score | C716 | 8846660 | Cerebellar glioma |
| Charlson Score | C716 | 8846661 | Cerebellar ependymoma |
| Charlson Score | C716 | 8847626 | Cerebellar pilocytic astrocytoma |
| Charlson Score | C716 | 8847834 | Malignant cerebellar tumor |
| Charlson Score | C716 | 8848720 | Cerebellar oligodendroglioma |
| Charlson Score | C717 | 1917002 | Pontine glioma |
| Charlson Score | C717 | 1917003 | Midbrain glioma |
| Charlson Score | C717 | 1917004 | Malignant neoplasm of brainstem |
| Charlson Score | C717 | 1917005 | Brain stem glioma |
| Charlson Score | C717 | 1919003 | Medullary glioma |
| Charlson Score | C717 | 8846825 | Ependymoma of the 4th ventricle |
| Charlson Score | C717 | 8846893 | Brain stem glioblastoma |
| Charlson Score | C717 | 8847590 | Medullary astrocytoma |
| Charlson Score | C717 | 8847669 | Brain stem astrocytoma |
| Charlson Score | C718 | 8849672 | Callosal glioblastoma |
| Charlson Score | C719 | 1919002 | Malignant glioma |
| Charlson Score | C719 | 1919004 | Spongioblastoma |
| Charlson Score | C719 | 1919007 | Oligodendroglioma |
| Charlson Score | C719 | 1919008 | Ependymoblastoma |
| Charlson Score | C719 | 1919009 | Ependymomas |
| Charlson Score | C719 | 1919015 | Glioma |
| Charlson Score | C719 | 1919022 | Astrocytoma |
| Charlson Score | C719 | 1919023 | Astroblastoma |
| Charlson Score | C719 | 1919024 | Astrocytoma |
| Charlson Score | C719 | 1919027 | Multiple gliomas |
| Charlson Score | C719 | 1919049 | Primary brain tumor |
| Charlson Score | C719 | 1919056 | Malignant astrocytoma |
| Charlson Score | C719 | 1919057 | Primary fibrillary astrocytoma |
| Charlson Score | C719 | 1919058 | Pilocytic astrocytoma |
| Charlson Score | C719 | 8830220 | Malignant brain tumor |
| Charlson Score | C719 | 8833379 | Glioblastoma |
| Charlson Score | C719 | 8835828 | Cranial chordoma |
| Charlson Score | C719 | 8838754 | Brain germ cell tumor |
| Charlson Score | C719 | 8842713 | Primitive neuroectodermal tumor |
| Charlson Score | C719 | 8846434 | Basilar chordoma |
| Charlson Score | C719 | 8847305 | Suprasellar germ cell tumor |
| Charlson Score | C719 | 8847406 | Intracranial germ cell tumor |
| Charlson Score | C719 | 8847614 | Gliosarcoma |
| Charlson Score | C719 | 8847657 | Anaplastic astrocytoma |
| Charlson Score | C719 | 8847681 | Diffuse astrocytoma |

| **Category** | **ICD10** | **Diagnosis Code** | **Diagnosis Name** |
| --- | --- | --- | --- |
| Charlson Score | C719 | 8847857 | Primary malignant brain tumor |
| Charlson Score | C719 | 8847882 | Anaplastic ependymomas |
| Charlson Score | C719 | 8848468 | Fornix hemangiopericytoma |
| Charlson Score | C719 | 8848615 | Middle cranial fossa hemangiopericytoma |
| Charlson Score | C719 | 8848621 | Intracranial hemangiopericytoma |
| Charlson Score | C719 | 8848738 | Anaplastic oligodendroglioma |
| Charlson Score | C719 | 8848739 | Anaplastic oligodendrogliocytoma |
| Charlson Score | C719 | 8848783 | Atypical teratoma-like/rhabdoid tumor |
| Charlson Score | C720 | 1922001 | Malignant neoplasm of spinal cord |
| Charlson Score | C720 | 1922003 | Spinal glioma |
| Charlson Score | C720 | 8846732 | Spinal ependymoma |
| Charlson Score | C720 | 8847608 | Cervical cord astrocytoma |
| Charlson Score | C720 | 8847646 | Spinal astrocytoma |
| Charlson Score | C721 | 8839110 | Cauda equina ependymoma |
| Charlson Score | C723 | 1920002 | Optic nerve glioma |
| Charlson Score | C724 | 8837734 | Acoustic glioma |
| Charlson Score | C725 | 8838716 | Maglignant neoplasm of cranial nerves |
| Charlson Score | C729 | 1929010 | Ganglioneuroblastoma |
| Charlson Score | C73 | 1930010 | Papillary thyroid carcinoma |
| Charlson Score | C73 | 1939001 | Malignant goiter |
| Charlson Score | C73 | 1939003 | Malignant thyroidal neoplasm |
| Charlson Score | C73 | 1939005 | Thyroid gland cancer |
| Charlson Score | C73 | 1939006 | Medullary carcinoma of thyroid |
| Charlson Score | C73 | 1939009 | Anaplastic thyroid cancer |
| Charlson Score | C73 | 1939010 | Thyroid follicular carcinoma |
| Charlson Score | C73 | 8849964 | Poorly differentiated thyroid carcinoma |
| Charlson Score | C740 | 8839697 | Adrenocortical carcinoma |
| Charlson Score | C740 | 8839703 | Malignant neoplasm of adrenal cortex |
| Charlson Score | C741 | 8830207 | Malignant phaeochromocytoma |
| Charlson Score | C741 | 8839687 | Malignant neoplasm of adrenal medulla |
| Charlson Score | C749 | 1940009 | Adrenal carcinoma |
| Charlson Score | C749 | 8832856 | Pheochromoblast |
| Charlson Score | C749 | 8839677 | Malignant adrenal neoplasm |
| Charlson Score | C749 | 8842758 | Neuroblastoma |
| Charlson Score | C749 | 8848098 | Adrenal neuroblastoma |
| Charlson Score | C750 | 1941004 | Parathyroid carcinoma |
| Charlson Score | C750 | 8844591 | Malignant parathyroid neoplasm |
| Charlson Score | C751 | 8842658 | Pituitary malignant tumor |
| Charlson Score | C752 | 8842662 | Malignant craniopharyngioma |
| Charlson Score | C753 | 8834684 | Pineoblastoma |
| Charlson Score | C753 | 8834687 | Pineal dysgerminoma |
| Charlson Score | C753 | 8842743 | Malignant neoplasm of pineal gland |
| Charlson Score | C753 | 8847350 | Pineal germ cell tumor |
| Charlson Score | C753 | 8847623 | Pineal glioblastoma |
| Charlson Score | C754 | 8842710 | Malignant neoplasm of carotid body |
| Charlson Score | C755 | 8847835 | Malignant paraganglioma |
| Charlson Score | C760 | 1733001 | Facial malignant tumor |
| Charlson Score | C760 | 1950005 | Malignant neoplasm of neck |
| Charlson Score | C760 | 1950007 | Neck cancer |

| **Category** | **ICD10** | **Diagnosis Code** | **Diagnosis Name** |
| --- | --- | --- | --- |
| Charlson Score | C760 | 1950008 | Head and neck cancer |
| Charlson Score | C760 | 8832993 | Primary neck tumor |
| Charlson Score | C760 | 8833002 | Neck neuroblastoma |
| Charlson Score | C760 | 8844153 | Malignant submandibular tumor |
| Charlson Score | C761 | 8830837 | Axillary carcinoma |
| Charlson Score | C761 | 8832584 | Chest cancer |
| Charlson Score | C761 | 8832601 | Chest neuroblastoma |
| Charlson Score | C762 | 8839723 | Malignant neoplasm of abdomen |
| Charlson Score | C762 | 8839730 | Abdominal neuroblastoma |
| Charlson Score | C763 | 8833843 | Pelvic neuroblastoma |
| Charlson Score | C763 | 8847385 | Sacrococcygeal germ cell tumor |
| Charlson Score | C764 | 8835420 | Malignant neoplasm of upper limb |
| Charlson Score | C765 | 8831206 | Malignant neoplasm of lower limb |
| Charlson Score | C770 | 1960005 | Neck lymph node metastasis |
| Charlson Score | C770 | 8834006 | Supraclavicular metastatic tumor |
| Charlson Score | C770 | 8842736 | Supraclavicular lymph node metastasis |
| Charlson Score | C771 | 8832487 | Malignant neoplasm of intrathoracic lymph nodes |
| Charlson Score | C771 | 8842764 | Mediastinal lymph node metastasis |
| Charlson Score | C771 | 8844689 | Bronchial lymph node metastasis |
| Charlson Score | C771 | 8845718 | Hilar lymph node metastasis |
| Charlson Score | C771 | 8848703 | Parasternal lymph node metastasis |
| Charlson Score | C772 | 8839655 | Malignant neoplasm of intraperitoneal lymph nodes |
| Charlson Score | C772 | 8839656 | Abdominal lymph node metastasis |
| Charlson Score | C772 | 8845660 | Retroperitoneal lymph node metastasis |
| Charlson Score | C772 | 8845706 | Periaortic lymph node metastasis |
| Charlson Score | C772 | 8845728 | Splenic lymph node metastasis |
| Charlson Score | C772 | 8848689 | Perigastric lymph node metastasis |
| Charlson Score | C772 | 8848695 | Hepatic lymph node metastasis |
| Charlson Score | C772 | 8848748 | Mesenteric lymph node metastasis |
| Charlson Score | C773 | 8842679 | Axillary lymph node metastasis |
| Charlson Score | C774 | 8836801 | Inguinal lymph node metastasis |
| Charlson Score | C775 | 8833837 | Malignant neoplasm of intrapelvic lymph nodes |
| Charlson Score | C775 | 8844873 | Pelvic lymph node metastasis |
| Charlson Score | C775 | 8845709 | Iliac lymph node metastasis |
| Charlson Score | C778 | 8845584 | Multiple-region lymph node metastases |
| Charlson Score | C779 | 1969001 | Lymph node metastases |
| Charlson Score | C780 | 1970005 | Metastatic lung cancer |
| Charlson Score | C780 | 1970006 | Metastatic lung neoplasm |
| Charlson Score | C781 | 1971002 | Metastatic mediastinal neoplasm |
| Charlson Score | C782 | 1972003 | Metastatic pleural neoplasm |
| Charlson Score | C782 | 1972004 | Carcinomatous pleurisy |
| Charlson Score | C782 | 8834664 | Neoplastic pleurisy |
| Charlson Score | C782 | 8847463 | Pleural dissemination |
| Charlson Score | C782 | 8847850 | Malignant pleural effusion |
| Charlson Score | C783 | 8847055 | Metastatic tracheal neoplasm |
| Charlson Score | C783 | 8847126 | Metastatic nasal cavity cancer |
| Charlson Score | C783 | 8847793 | Metastatic ethmoid sinus cancer |

| **Category** | **ICD10** | **Diagnosis Code** | **Diagnosis Name** |
| --- | --- | --- | --- |
| Charlson Score | C783 | 8847794 | Metastatic maxillary antrum cancer |
| Charlson Score | C783 | 8847795 | Metastatic frontal sinus cancer |
| Charlson Score | C783 | 8847796 | Metastatic sphenoidal sinus cancer |
| Charlson Score | C783 | 8847797 | Metastatic paranasal sinus cancer |
| Charlson Score | C784 | 8837929 | Metastatic small intestinal neoplasm |
| Charlson Score | C784 | 8844084 | Metastatic duodenal carcinoma |
| Charlson Score | C785 | 8837931 | Metastatic colorectal neoplasm |
| Charlson Score | C785 | 8842810 | Metastatic rectal neoplasm |
| Charlson Score | C786 | 1976001 | Carcinomatous peritonitis |
| Charlson Score | C786 | 2354005 | Pseudomyxoma peritonei |
| Charlson Score | C786 | 7895002 | Carcinomatous ascites |
| Charlson Score | C786 | 8837926 | Metastatic retroperitoneal neoplasm |
| Charlson Score | C786 | 8839765 | Peritoneal metastases |
| Charlson Score | C786 | 8842314 | Peritoneal dissemination |
| Charlson Score | C787 | 1977005 | Metastatic liver cancer |
| Charlson Score | C787 | 1977006 | Metastatic hepatic neoplasm |
| Charlson Score | C788 | 8837928 | Metastatic gastrointestinal neoplasm |
| Charlson Score | C788 | 8837930 | Metastatic pancreatic neoplasm |
| Charlson Score | C788 | 8837933 | Metastatic splenic tumor |
| Charlson Score | C790 | 1980004 | Metastatic renal tumor |
| Charlson Score | C791 | 1981002 | Metastatic bladder cancer |
| Charlson Score | C792 | 1982004 | Metastatic skin tumor |
| Charlson Score | C792 | 1982005 | Skin metastases from carcinoma breast |
| Charlson Score | C792 | 8842031 | Leukemia cutis |
| Charlson Score | C792 | 8847798 | Metastatic abdominal wall neoplasm |
| Charlson Score | C793 | 1921008 | Meningeal carcinomatosis |
| Charlson Score | C793 | 1983001 | Metastatic supratentorial and infratentorial neoplasm |
| Charlson Score | C793 | 1983003 | Metastatic occipital tumor |
| Charlson Score | C793 | 1983005 | Metastatic frontal tumor |
| Charlson Score | C793 | 1983006 | Metastatic temporal tumor |
| Charlson Score | C793 | 1983009 | Metastatic deep-cerebrum tumor |
| Charlson Score | C793 | 1983019 | Metastatic brain tumor |
| Charlson Score | C793 | 1983025 | Spinal cord dissemination |
| Charlson Score | C793 | 8835803 | Meningeal leukemia |
| Charlson Score | C794 | 1906005 | Metastatic choroidal cancer |
| Charlson Score | C794 | 1983014 | Metastatic spinal cord tumor |
| Charlson Score | C794 | 1984001 | Metastatic spinal epidural tumor |
| Charlson Score | C794 | 1984002 | Metastatic intradural extramedullary spinal cord tumor |
| Charlson Score | C795 | 1985007 | Metastatic bone cancer |
| Charlson Score | C795 | 1985008 | Pelvic metastases |
| Charlson Score | C795 | 1985010 | Bone metastases from uterine carcinoma |
| Charlson Score | C795 | 1985023 | Metastatic bone tumor |
| Charlson Score | C795 | 1985024 | Metastatic skull tumor |
| Charlson Score | C795 | 1985025 | Bone metastases from carcinoma breast |
| Charlson Score | C795 | 8836022 | Spinal metastases |
| Charlson Score | C795 | 8837313 | Femoral metastatic bone tumor |
| Charlson Score | C795 | 8841259 | Rib metastases |

| **Category** | **ICD10** | **Diagnosis Code** | **Diagnosis Name** |
| --- | --- | --- | --- |
| Charlson Score | C795 | 8842124 | Bone marrow infiltration by multiple myeloma |
| Charlson Score | C795 | 8842125 | Bone marrow infiltration by myeloid leukemia |
| Charlson Score | C795 | 8842126 | Bone marrow infiltration by adult T-cell leukemia |
| Charlson Score | C795 | 8842127 | Bone marrow infiltration by lymphatic leukemia |
| Charlson Score | C795 | 8842667 | Bone metastases from stomach carcinoma |
| Charlson Score | C795 | 8842692 | Bone metastases from carcinoma of liver |
| Charlson Score | C795 | 8842718 | Bone metastases from thyroid gland cancer |
| Charlson Score | C795 | 8842771 | Bone metastases from renal carcinoma |
| Charlson Score | C795 | 8842788 | Bone metastases from prostatic carcinoma |
| Charlson Score | C795 | 8842830 | Bone metastases from pulmonary carcinoma |
| Charlson Score | C795 | 8842905 | Thoracic metastases |
| Charlson Score | C795 | 8843015 | Lumbar metastases |
| Charlson Score | C795 | 8843072 | Bone metastases from oesophageal cancer |
| Charlson Score | C795 | 8843075 | Bone metastases from pancreatic cancer |
| Charlson Score | C795 | 8843085 | Bone metastases from large intestine carcinoma |
| Charlson Score | C795 | 8843088 | Bone metastases from rectal carcinoma |
| Charlson Score | C795 | 8843434 | Metastatic mandibular cancer |
| Charlson Score | C795 | 8843437 | Metastatic maxillary cancer |
| Charlson Score | C795 | 8844349 | Bone marrow infiltration by malignant lymphoma |
| Charlson Score | C795 | 8844442 | Bone marrow metastases |
| Charlson Score | C795 | 8846021 | Fracture femur due to metastatic bone tumor |
| Charlson Score | C795 | 8848241 | Cervical metastases |
| Charlson Score | C795 | 8849333 | Neoplastic pathologic fracture of thoracic vertebra |
| Charlson Score | C795 | 8849334 | Neoplastic pathologic fracture of finger |
| Charlson Score | C795 | 8849335 | Neoplastic pathologic fracture of humerus |
| Charlson Score | C795 | 8849336 | Neoplastic pathologic fracture of spine |
| Charlson Score | C795 | 8849337 | Neoplastic pathologic fracture of sacrum |
| Charlson Score | C795 | 8849338 | Neoplastic pathologic fractures of the 1st and 2nd thoracic vertebras |
| Charlson Score | C795 | 8849339 | Neoplastic pathologic fracture of the 2nd lumbar vertebra |
| Charlson Score | C795 | 8849340 | Neoplastic pathologic fracture of the 3rd lumbar vertebra |
| Charlson Score | C795 | 8849341 | Neoplastic pathologic fracture of the 4th lumbar vertebra |
| Charlson Score | C795 | 8849342 | Neoplastic pathologic fracture of the 5th lumbar vertebra |
| Charlson Score | C795 | 8849343 | Neoplastic pathologic fracture of femur |
| Charlson Score | C795 | 8849344 | Neoplastic pathologic fracture of pubis |
| Charlson Score | C795 | 8849345 | Neoplastic pathologic fracture |
| Charlson Score | C795 | 8849346 | Neoplastic pathologic fracture of lumbar vertebra |
| Charlson Score | C795 | 8849347 | Neoplastic pathologic fracture of rib |
| Charlson Score | C796 | 1986005 | Metastatic ovarian cancer |
| Charlson Score | C796 | 8832841 | Krukenberg tumor |
| Charlson Score | C797 | 8837934 | Metastatic adrenal tumor |

| **Category** | **ICD10** | **Diagnosis Code** | **Diagnosis Name** |
| --- | --- | --- | --- |
| Charlson Score | C798 | 1988009 | Metastatic neck tumor |
| Charlson Score | C798 | 2398068 | Metastatic cardiac tumor |
| Charlson Score | C798 | 4239002 | Carcinomatous pericarditis |
| Charlson Score | C798 | 4572003 | Carcinomatous lymphangitis |
| Charlson Score | C798 | 8833010 | Metastatic neck adenocarcinoma |
| Charlson Score | C798 | 8837932 | Metastatic vaginal tumor |
| Charlson Score | C798 | 8843435 | Metastatic chest wall tumor |
| Charlson Score | C798 | 8843436 | Metastatic oral carcinoma |
| Charlson Score | C798 | 8843438 | Metastatic tongue cancer |
| Charlson Score | C798 | 8845248 | Lymphangitis carcinomatosis |
| Charlson Score | C798 | 8845443 | Metastatic uterine carcinoma |
| Charlson Score | C798 | 8848159 | Metastatic prostate tumor |
| Charlson Score | C798 | 8848470 | Diaphragmatic metastases |
| Charlson Score | C798 | 8848981 | Metastatic carcinoma breast |
| Charlson Score | C798 | 8850285 | Metastatic soft tissue tumor |
| Charlson Score | C799 | 1729020 | Metastatic melanoma |
| Charlson Score | C799 | 1990004 | Systemic metastatic cancer |
| Charlson Score | C799 | 1990009 | Multiple cancer metastases |
| Charlson Score | C799 | 1991019 | Metastatic tumor |
| Charlson Score | C799 | 1991030 | Systemic metastases from ovarian carcinoma |
| Charlson Score | C799 | 8837935 | Metastatic carcinoma squamous |
| Charlson Score | C800 | 1991011 | Unknown primary cancer |
| Charlson Score | C800 | 8849128 | Neuroendocrine cell carcinoma of unknown primary site |
| Charlson Score | C800 | 8849130 | Neuroendocrine tumor of unknown primary site |
| Charlson Score | C809 | 1830013 | Germinoma |
| Charlson Score | C809 | 1958001 | Chordoma |
| Charlson Score | C809 | 1990013 | Double cancer |
| Charlson Score | C809 | 1991004 | Malignant teratoma |
| Charlson Score | C809 | 1991086 | Embryonal carcinoma |
| Charlson Score | C809 | 1991089 | Endodermal sinus tumor |
| Charlson Score | C809 | 7103002 | Dermatomyositis with malignancy |
| Charlson Score | C809 | 8830213 | Malignant tumor |
| Charlson Score | C809 | 8830214 | Anemia with malignancy |
| Charlson Score | C809 | 8831468 | Carcinoid |
| Charlson Score | C809 | 8831889 | Carcinoma |
| Charlson Score | C809 | 8832025 | Cancer cachexia |
| Charlson Score | C809 | 8832027 | Carcinomatous neuropathy |
| Charlson Score | C809 | 8832030 | Cancer anemia |
| Charlson Score | C809 | 8835466 | Epithelioma |
| Charlson Score | C809 | 8840249 | End stage cancer |
| Charlson Score | C809 | 8840887 | Yolk sac tumor |
| Charlson Score | C809 | 8841315 | Eaton-Lambert syndrome |
| Charlson Score | C809 | 8841420 | Carcinomatous neuromyopathy |
| Charlson Score | C809 | 8841441 | Carcinomatous myelopathy |
| Charlson Score | C809 | 8843069 | Paraneoplastic syndromes |
| Charlson Score | C809 | 8845247 | Cancer-associated retinopathy |
| Charlson Score | C809 | 8848236 | Carcinomatous skin ulceration |
| Charlson Score | C809 | 8848997 | Paraneoplastic limbic encephalitis |

| **Category** | **ICD10** | **Diagnosis Code** | **Diagnosis Name** |
| --- | --- | --- | --- |
| Charlson Score | C809 | 8849127 | Neuroendocrine cell carcinoma |
| Charlson Score | C809 | 8849129 | Neuroendocrine tumor |
| Charlson Score | C809 | 8849706 | Malignant rhabdoid tumor |
| Charlson Score | C809 | 8849753 | Mixed germ cell tumor |
| Charlson Score | C809 | 8849851 | Lynch syndrome |
| Charlson Score | C809 | 8850206 | Paraneoplastic cerebellar degeneration |
| Charlson Score | C809 | 8850330 | Paraneoplastic opsoclonus-myoclonus syndrome |
| Charlson Score | C810 | 8841112 | Hodgkin’s disease lymphocyte predominance type |
| Charlson Score | C810 | 8847329 | Nodular lymphocyte predominant Hodgkin lymphoma |
| Charlson Score | C811 | 8833116 | Hodgkin’s disease nodular sclerosis type |
| Charlson Score | C811 | 8847328 | Nodular sclerosis classical Hodgkin lymphoma |
| Charlson Score | C812 | 8833890 | Hodgkin’s disease mixed cellularity type |
| Charlson Score | C812 | 8847339 | Mixed cellularity classical Hodgkin lymphoma |
| Charlson Score | C813 | 8841109 | Hodgkin’s disease lymphocyte depletion type |
| Charlson Score | C813 | 8847439 | Lymphocyte depletion classical Hodgkin lymphoma |
| Charlson Score | C814 | 8847440 | Lymphocyte-rich classical Hodgkin lymphoma |
| Charlson Score | C817 | 8847338 | Classical Hodgkin lymphoma |
| Charlson Score | C819 | 2012002 | Hodgkin lymphoma |
| Charlson Score | C819 | 2019001 | Lymphomas Hodgkin’s disease |
| Charlson Score | C820 | 8841699 | Follicular lymphoma, medium-sized cell type |
| Charlson Score | C820 | 8847444 | Follicular lymphoma grade I |
| Charlson Score | C821 | 8841700 | Follicular lymphoma, mixed type |
| Charlson Score | C821 | 8847445 | Follicular lymphoma grade II |
| Charlson Score | C823 | 8847446 | Follicular lymphoma grade IIIa |
| Charlson Score | C824 | 8841701 | Follicular lymphoma, large-sized cell type |
| Charlson Score | C824 | 8847447 | Follicular lymphoma grade IIIb |
| Charlson Score | C826 | 8847332 | Primary cutaneous follicle centre lymphoma |
| Charlson Score | C827 | 8847357 | Paediatric-type follicular lymphoma |
| Charlson Score | C829 | 8841397 | Follicular lymphoma |
| Charlson Score | C830 | 8834717 | Diffuse lymphoma, small-sized cell type |
| Charlson Score | C830 | 8847354 | Paediatric-type nodal marginal zone lymphoma |
| Charlson Score | C830 | 8847359 | Small cell lymphocytic lymphoma |
| Charlson Score | C830 | 8847382 | Nodal marginal zone lymphoma |
| Charlson Score | C830 | 8847412 | Splenic B-cell lymphoma/leukemia unclassifiable |
| Charlson Score | C830 | 8847414 | Splenic diffuse red pulp small B-cell lymphoma |
| Charlson Score | C830 | 8847419 | Splenic marginal zone lymphoma |
| Charlson Score | C830 | 8847427 | Hairy cell leukemia subtype |
| Charlson Score | C830 | 8847441 | Lymphoplasmacytic lymphoma |
| Charlson Score | C831 | 8833887 | Diffuse lymphoma, mixed type |
| Charlson Score | C831 | 8834696 | Diffuse lymphoma, small cleaved-cell type |
| Charlson Score | C831 | 8846346 | Mantle cell lymphoma |
| Charlson Score | C833 | 8837272 | Diffuse lymphoma, large-sized cell type |
| Charlson Score | C833 | 8847273 | ALK-positive large B-cell lymphoma |
| Charlson Score | C833 | 8847300 | T-cell/histiocyte-rich large B-cell lymphoma |

| **Category** | **ICD10** | **Diagnosis Code** | **Diagnosis Name** |
| --- | --- | --- | --- |
| Charlson Score | C833 | 8847324 | Plasmablastic lymphoma |
| Charlson Score | C833 | 8847337 | EBV-positive diffuse large B-cell lymphoma of the elderly |
| Charlson Score | C833 | 8847396 | Primary CNS diffuse large B-cell lymphoma |
| Charlson Score | C833 | 8847418 | Primary cutaneous diffuse large B-cell lymphoma of lower limb type |
| Charlson Score | C833 | 8847422 | Diffuse large B-cell lymphoma |
| Charlson Score | C833 | 8847432 | Chronic inflammation-associated diffuse large B-cell lymphoma |
| Charlson Score | C835 | 8841107 | Lymphoblastic lymphoma |
| Charlson Score | C835 | 8847277 | BCR-ABL1-positive B-lymphoblastic lymphoma |
| Charlson Score | C835 | 8847281 | B-lymphoblastic lymphoma |
| Charlson Score | C835 | 8847285 | E2A-PBX1-positive B-lymphoblastic lymphoma |
| Charlson Score | C835 | 8847291 | IL3-IGH-positive B-lymphoblastic lymphoma |
| Charlson Score | C835 | 8847294 | MLL-rearranged B-lymphoblastic lymphoma |
| Charlson Score | C835 | 8847297 | TEL-AML1-positive B-lymphoblastic lymphoma |
| Charlson Score | C835 | 8847303 | T-lymphoblastic lymphoma |
| Charlson Score | C835 | 8847335 | Hyperdiploid B-lymphoblastic lymphoma |
| Charlson Score | C835 | 8847403 | Hypodiploid B-lymphoblastic lymphoma |
| Charlson Score | C837 | 8839053 | Burkitt’s lymphoma |
| Charlson Score | C838 | 8847286 | Large B-cell lymphoma arising in HHV8 associated multicentric Castleman’s disease |
| Charlson Score | C838 | 8847326 | Intravascular large B-cell lymphoma |
| Charlson Score | C838 | 8847331 | Primary effusion lymphoma |
| Charlson Score | C838 | 8847420 | B-cell lymphoma, unclassifiable, with features intermediate between diffuse large B-cell lymphoma and Burkitt’s lymphoma |
| Charlson Score | C838 | 8847421 | B-cell lymphoma, unclassifiable, with features intermediate between diffuse large B-cell lymphoma and Hodgkin’s lymphoma |
| Charlson Score | C838 | 8847442 | Lymphomatoid granulomatosis |
| Charlson Score | C840 | 8842177 | Mycosis fungoides |
| Charlson Score | C841 | 2022001 | Sezary’s syndrome |
| Charlson Score | C844 | 8830149 | T-zone lymphoma |
| Charlson Score | C844 | 8840258 | Peripheral T-cell lymphoma |
| Charlson Score | C844 | 8841201 | Lennert lymphoma |
| Charlson Score | C844 | 8847430 | Peripheral T-cell lymphoma unspecified |
| Charlson Score | C844 | 8848111 | CCR4-positive peripheral T-cell lymphoma |
| Charlson Score | C845 | 8847351 | EBV-positive T-cell lymphoproliferative disease of childhood |
| Charlson Score | C845 | 8847355 | Systematic EBV-positive T-cell lymphoproliferative disease of childhood |
| Charlson Score | C845 | 8849682 | Chronic active EB virus infection |
| Charlson Score | C846 | 8840439 | Diffuse anaplastic lymphoma |
| Charlson Score | C846 | 8847274 | ALK-positive anaplastic large-cell lymphoma |
| Charlson Score | C846 | 8847433 | Anaplastic large-cell lymphoma |
| Charlson Score | C847 | 8847271 | ALK-negative anaplastic large-cell lymphoma |
| Charlson Score | C848 | 8847348 | Hydroa vacciniforme-like lymphoma |
| Charlson Score | C848 | 8847416 | Primary cutaneous γδ T-cell lymphoma |

| **Category** | **ICD10** | **Diagnosis Code** | **Diagnosis Name** |
| --- | --- | --- | --- |
| Charlson Score | C848 | 8848110 | CCR4-positive cutaneous T-cell lymphoma |
| Charlson Score | C848 | 8848162 | Cutaneous T-cell lymphoma |
| Charlson Score | C851 | 8830067 | B-cell lymphoma |
| Charlson Score | C851 | 8848431 | CD20-positive B-cell non-Hodgkin’s lymphoma |
| Charlson Score | C851 | 8849059 | B-cell non-Hodgkin’s lymphoma |
| Charlson Score | C852 | 8847364 | Primary mediastinal large B-cell lymphoma |
| Charlson Score | C859 | 1591001 | Splenic malignant lymphoma |
| Charlson Score | C859 | 1901006 | Orbital malignant lymphoma |
| Charlson Score | C859 | 2028005 | Malignant lymphoma |
| Charlson Score | C859 | 2028006 | Gastric malignant lymphoma |
| Charlson Score | C859 | 2028011 | Duodenal malignant lymphoma |
| Charlson Score | C859 | 2028012 | Mediastinal malignant lymphoma |
| Charlson Score | C859 | 2028017 | Non-Hodgkin’s lymphoma |
| Charlson Score | C859 | 2028041 | Thyroid malignant lymphoma |
| Charlson Score | C859 | 2028048 | Tonsillar malignant lymphoma |
| Charlson Score | C859 | 8833789 | Malignant lymphoma of bone |
| Charlson Score | C859 | 8833984 | Reticulosarcoma |
| Charlson Score | C859 | 8837363 | Colonic malignant lymphoma |
| Charlson Score | C859 | 8838679 | Cerebral malignant lymphoma |
| Charlson Score | C859 | 8839552 | Diffuse lymphoma |
| Charlson Score | C859 | 8841113 | Lymphoma |
| Charlson Score | C859 | 8844042 | Cervical malignant lymphoma |
| Charlson Score | C859 | 8844043 | Colonic malignant lymphoma |
| Charlson Score | C859 | 8844058 | Small intestine malignant lymphoma |
| Charlson Score | C859 | 8844062 | Heart malignant lymphoma |
| Charlson Score | C859 | 8844083 | Rectal malignant lymphoma |
| Charlson Score | C859 | 8845971 | Testicular malignant lymphoma |
| Charlson Score | C859 | 8846037 | Pyothorax-associated lymphoma |
| Charlson Score | C883 | 8830368 | Alpha heavy chain disease |
| Charlson Score | C883 | 8840562 | Immunoproliferative small bowel disease |
| Charlson Score | C883 | 8847157 | Alpha heavy chain disease |
| Charlson Score | C900 | 2030003 | Multiple myeloma |
| Charlson Score | C900 | 8830205 | Malignant monoclonal gammopathy |
| Charlson Score | C900 | 8832852 | Crow-Fukase syndrome |
| Charlson Score | C900 | 8837022 | Multiple myeloma arthrosis |
| Charlson Score | C900 | 8839397 | Nonsecretory multiple myeloma |
| Charlson Score | C900 | 8840039 | Bence Jones multiple myeloma |
| Charlson Score | C900 | 8842090 | Myeloma kidney |
| Charlson Score | C900 | 8847152 | POEMS syndrome |
| Charlson Score | C900 | 8847175 | Plasma cell myelomas |
| Charlson Score | C900 | 8847250 | Nonsecretory myeloma |
| Charlson Score | C900 | 8847258 | Asymptomatic myeloma |
| Charlson Score | C901 | 2031001 | Plasma cell leukemia |
| Charlson Score | C910 | 2040003 | Acute lymphoblastic leukemia |
| Charlson Score | C910 | 8846350 | Ph-positive acute lymphocytic leukemia |
| Charlson Score | C910 | 8847275 | BCR-ABL1-positive B-lymphoblastic leukemia |
| Charlson Score | C910 | 8847276 | BCR-ABL1-positive B-lymphoblastic leukemia/lymphoma |
| Charlson Score | C910 | 8847279 | B-lymphoblastic leukemia |

| **Category** | **ICD10** | **Diagnosis Code** | **Diagnosis Name** |
| --- | --- | --- | --- |
| Charlson Score | C910 | 8847280 | B-lymphoblastic leukemia/lymphoma |
| Charlson Score | C910 | 8847283 | E2A-PBX1-positive B-lymphoblastic leukemia |
| Charlson Score | C910 | 8847284 | E2A-PBX1-positive B-lymphoblastic leukemia/lymphoma |
| Charlson Score | C910 | 8847289 | IL3-IGH-positive B-lymphoblastic leukemia |
| Charlson Score | C910 | 8847290 | IL3-IGH-positive B-lymphoblastic leukemia/lymphoma |
| Charlson Score | C910 | 8847292 | MLL-rearranged B-lymphoblastic leukemia |
| Charlson Score | C910 | 8847293 | MLL-rearranged B-lymphoblastic leukemia/lymphoma |
| Charlson Score | C910 | 8847295 | TEL-AML1-positive B-lymphoblastic leukemia |
| Charlson Score | C910 | 8847296 | TEL-AML1-positive B-lymphoblastic leukemia/lymphoma |
| Charlson Score | C910 | 8847301 | T-lymphoblastic leukemia |
| Charlson Score | C910 | 8847302 | T-lymphoblastic leukemia/lymphoma |
| Charlson Score | C910 | 8847333 | Hyperdiploid B-lymphoblastic leukemia |
| Charlson Score | C910 | 8847334 | Hyperdiploid B-lymphoblastic leukemia/lymphoma |
| Charlson Score | C910 | 8847352 | Acute lymphocytic leukemia in children |
| Charlson Score | C910 | 8847401 | Hypodiploid B-lymphoblastic leukemia |
| Charlson Score | C910 | 8847402 | Hypodiploid B-lymphoblastic leukemia/lymphoma |
| Charlson Score | C911 | 2041001 | Chronic lymphocytic leukemia |
| Charlson Score | C911 | 8830066 | B-cell chronic lymphocytic leukemia |
| Charlson Score | C913 | 2049005 | Prolymphocytic leukemia |
| Charlson Score | C913 | 8847278 | B-cell prolymphocytic leukemia |
| Charlson Score | C914 | 8839929 | Hairy cell leukemia |
| Charlson Score | C914 | 8847426 | Hairy cell leukemia |
| Charlson Score | C915 | 8832798 | Smoldering acute leukemia |
| Charlson Score | C915 | 8835876 | Adult T-cell leukemia |
| Charlson Score | C915 | 8835877 | Adult T-cell lymphoma |
| Charlson Score | C915 | 8847282 | CCR4-positive adult T-cell leukemia-lymphoma |
| Charlson Score | C915 | 8847374 | Adult T-cell leukemia-lymphoma |
| Charlson Score | C915 | 8847375 | Adult T-cell leukemia-lymphoma of acute type |
| Charlson Score | C915 | 8847376 | Adult T-cell leukemia-lymphoma of smoldering type |
| Charlson Score | C915 | 8847377 | Adult T-cell leukemia-lymphoma of chronic type |
| Charlson Score | C915 | 8847378 | Adult T-cell leukemia-lymphoma of lymphoma type |
| Charlson Score | C916 | 8847298 | T-cell prolymphocytic leukemia |
| Charlson Score | C917 | 8847299 | T-cell large granular lymphocytic leukemia |
| Charlson Score | C917 | 8847431 | Chronic NK-cell lymphoproliferative disorder |
| Charlson Score | C918 | 8847411 | Burkitt’s leukemia |
| Charlson Score | C919 | 2049003 | Lymphatic leukemia |
| Charlson Score | C920 | 2050003 | Acute myeloid leukemia |
| Charlson Score | C920 | 8830132 | RAEB-t |
| Charlson Score | C920 | 8849738 | Acute myeloid leukemia of the least anaplastic type |
| Charlson Score | C920 | 8849786 | Acute myeloid leukemia with maturation |

| **Category** | **ICD10** | **Diagnosis Code** | **Diagnosis Name** |
| --- | --- | --- | --- |
| Charlson Score | C920 | 8849787 | Acute myeloid leukemia without maturation |
| Charlson Score | C920 | 8849858 | FLT3-ITD mutation-positive acute myeloid leukemia |
| Charlson Score | C921 | 2051004 | Chronic myeloid leukemia |
| Charlson Score | C921 | 8841651 | Chronic myeloid leukemia transformation |
| Charlson Score | C921 | 8841652 | Chronic phase chronic myeloid leukemia |
| Charlson Score | C921 | 8841653 | Accelerated phase chronic myeloid leukemia |
| Charlson Score | C922 | 8842982 | Atypical chronic myeloid leukemia |
| Charlson Score | C923 | 8842897 | Granulocytic sarcoma |
| Charlson Score | C924 | 2050004 | Acute promyelocytic leukemia |
| Charlson Score | C925 | 2050008 | Acute myelomonocytic leukemia |
| Charlson Score | C927 | 8833339 | Leukemia basophilic |
| Charlson Score | C927 | 8838402 | Secondary leukemias |
| Charlson Score | C927 | 8848483 | Blastic plasmacytoid dendritic cell neoplasia |
| Charlson Score | C928 | 8849750 | Acute myeloid leukemia with myelodysplasia- related changes |
| Charlson Score | C929 | 2059002 | Myelogenous leukemia |
| Charlson Score | C929 | 2089007 | Atypical leukemia |
| Charlson Score | C929 | 8837869 | Hypoplastic leukemia |
| Charlson Score | C930 | 2060001 | Acute monocytic leukemia |
| Charlson Score | C931 | 2051005 | Chronic myelomonocytic leukemia |
| Charlson Score | C931 | 8833810 | Myelomonocytic leukemia |
| Charlson Score | C931 | 8840368 | Chronic monocytic leukemia |
| Charlson Score | C933 | 8842929 | Juvenile myelomonocytic leukemia |
| Charlson Score | C939 | 8837107 | Monocytic leukemia |
| Charlson Score | C940 | 2070003 | Erythroleukemia |
| Charlson Score | C942 | 8832328 | Acute megakaryoblastic leukemia |
| Charlson Score | C943 | 8839403 | Mastocytic leukemia |
| Charlson Score | C947 | 8847304 | Aggressive NK-cell leukemia |
| Charlson Score | C947 | 8849702 | NK-cell leukemia |
| Charlson Score | C950 | 2080001 | Leukemia acute |
| Charlson Score | C950 | 8833886 | Mixed leukemia |
| Charlson Score | C951 | 2081001 | Leukemia chronic |
| Charlson Score | C959 | 2089006 | Leukemia |
| Charlson Score | C959 | 8838956 | Leukemic arthritis |
| Charlson Score | C960 | 8841165 | Letterer-Siwe disease |
| Charlson Score | C962 | 8830222 | Malignant mastocytoma |
| Charlson Score | C964 | 8842921 | Interdigitating dendritic cell sarcoma |
| Charlson Score | C964 | 8843022 | Follicular dendritic cell tumor |
| Charlson Score | C965 | 8839024 | Hand-Schüller-Christian disease |
| Charlson Score | C966 | 8833451 | Eosinophilic granuloma |
| Charlson Score | C966 | 8839288 | Histiocytosis X |
| Charlson Score | C966 | 8840901 | Langerhans’ cell histiocytosis |
| Charlson Score | C966 | 8842097 | Eosinophilic granulomatosis |
| Charlson Score | C968 | 8830217 | Malignant histiocytosis |
| Charlson Score | C968 | 8830218 | Malignant histiocytosis arthrosis |
| Charlson Score | E101 | 8830028 | Type 1 diabetic ketoacidosis |
| Charlson Score | E101 | 8841680 | Type 1 diabetes mellitus with ketoacidosis |

| **Category** | **ICD10** | **Diagnosis Code** | **Diagnosis Name** |
| --- | --- | --- | --- |
| Charlson Score | E101 | 8844025 | Slowly progressive type 1 diabetes mellitus with ketoacidosis |
| Charlson Score | E101 | 8844045 | Fulminant type 1 diabetes mellitus |
| Charlson Score | E101 | 8845044 | Type 1 diabetic acidosis |
| Charlson Score | E101 | 8845045 | Type 1 Diabetic Acetonemia |
| Charlson Score | E101 | 8849056 | Type 1 diabetic ketosis |
| Charlson Score | E102 | 8830031 | Type 1 diabetic nephropathy |
| Charlson Score | E102 | 8841681 | Type 1 diabetes mellitus with renal complications |
| Charlson Score | E102 | 8843983 | Type 1 diabetic nephropathy stage 1 |
| Charlson Score | E102 | 8843984 | Type 1 diabetic nephropathy stage 2 |
| Charlson Score | E102 | 8843985 | Type 1 diabetic nephropathy stage 3 |
| Charlson Score | E102 | 8843986 | Type 1 diabetic nephropathy stage 3A |
| Charlson Score | E102 | 8843987 | Type 1 diabetic nephropathy stage 3B |
| Charlson Score | E102 | 8843988 | Type 1 diabetic nephropathy stage 4 |
| Charlson Score | E102 | 8843989 | Type 1 diabetic nephropathy stage 5 |
| Charlson Score | E102 | 8844028 | Slowly progressive type 1 diabetes mellitus with renal complications |
| Charlson Score | E102 | 8845058 | Type 1 diabetic renal sclerosis |
| Charlson Score | E102 | 8845059 | Type 1 diabetic kidney failure |
| Charlson Score | E103 | 8830033 | Type 1 diabetic retinopathy |
| Charlson Score | E103 | 8841682 | Type 1 diabetes mellitus with ophthalmic complications |
| Charlson Score | E103 | 8843982 | Type 1 diabetic macular oedema |
| Charlson Score | E103 | 8844024 | Slowly progressive type 1 diabetes mellitus with ophthalmic complications |
| Charlson Score | E103 | 8844346 | Type 1 diabetic cataract |
| Charlson Score | E103 | 8844536 | Proliferative type 1 diabetic retinopathy |
| Charlson Score | E103 | 8845043 | Type 1 diabetic maculopathy |
| Charlson Score | E103 | 8845049 | Type 1 diabetic ophthalmoplegia |
| Charlson Score | E103 | 8845053 | Type 1 diabetic iritis |
| Charlson Score | E103 | 8845064 | Type 1 diabetic central retinopathy |
| Charlson Score | E104 | 8830032 | Type 1 diabetic neuropathy |
| Charlson Score | E104 | 8841683 | Type 1 diabetes mellitus with neurological complications |
| Charlson Score | E104 | 8844027 | Slowly progressive type 1 diabetes mellitus with neurological complications |
| Charlson Score | E104 | 8845050 | Type 1 diabetic amyotrophy |
| Charlson Score | E104 | 8845055 | Type 1 diabetic neuropathic bladder |
| Charlson Score | E104 | 8845056 | Type 1 diabetic neuralgia |
| Charlson Score | E104 | 8845057 | Type 1 diabetic autonomic neuropathy |
| Charlson Score | E104 | 8845062 | Type 1 diabetic polyneuropathy |
| Charlson Score | E104 | 8845063 | Type 1 diabetic mononeuropathy |
| Charlson Score | E104 | 8845071 | Type 1 diabetic peripheral neuropathy |
| Charlson Score | E105 | 8841684 | Type 1 diabetes mellitus with peripheral circulatory complications |
| Charlson Score | E105 | 8843105 | Type 1 diabetic gangrene |
| Charlson Score | E105 | 8844031 | Slowly progressive type 1 diabetes mellitus with peripheral circulatory complications |

| **Category** | **ICD10** | **Diagnosis Code** | **Diagnosis Name** |
| --- | --- | --- | --- |
| Charlson Score | E105 | 8845046 | Type 1 diabetic ulcer |
| Charlson Score | E105 | 8845051 | Type 1 diabetic vascular disorders |
| Charlson Score | E105 | 8845066 | Type 1 diabetic arteriosclerosis |
| Charlson Score | E105 | 8845067 | Type 1 diabetic arterial occlusion |
| Charlson Score | E105 | 8845069 | Type 1 diabetic peripheral angiopathy |
| Charlson Score | E105 | 8845070 | Type 1 diabetic peripheral vascular disease |
| Charlson Score | E109 | 8841688 | Type 1 diabetes mellitus without complications |
| Charlson Score | E109 | 8844030 | Slowly progressive type 1 diabetes mellitus without complications |
| Charlson Score | E111 | 8830040 | Type 2 diabetic ketoacidosis |
| Charlson Score | E111 | 8841690 | Type 2 diabetes mellitus with ketoacidosis |
| Charlson Score | E111 | 8845073 | Type 2 diabetic acidosis |
| Charlson Score | E111 | 8845074 | Type 2 diabetic acetonemia |
| Charlson Score | E111 | 8849058 | Type 2 diabetic ketosis |
| Charlson Score | E112 | 8830042 | Type 2 diabetic nephropathy |
| Charlson Score | E112 | 8841691 | Type 2 diabetes mellitus with renal complications |
| Charlson Score | E112 | 8843991 | Type 2 diabetic nephropathy stage 1 |
| Charlson Score | E112 | 8843992 | Type 2 diabetic nephropathy stage 2 |
| Charlson Score | E112 | 8843993 | Type 2 diabetic nephropathy stage 3 |
| Charlson Score | E112 | 8843994 | Type 2 diabetic nephropathy stage 3A |
| Charlson Score | E112 | 8843995 | Type 2 diabetic nephropathy stage 3B |
| Charlson Score | E112 | 8843996 | Type 2 diabetic nephropathy stage 4 |
| Charlson Score | E112 | 8843997 | Type 2 diabetic nephropathy stage 5 |
| Charlson Score | E112 | 8845087 | Type 2 diabetic renal sclerosis |
| Charlson Score | E112 | 8845088 | Type 2 diabetic kidney failure |
| Charlson Score | E113 | 8830045 | Type 2 diabetic retinopathy |
| Charlson Score | E113 | 8841692 | Type 2 diabetes mellitus with ophthalmic complications |
| Charlson Score | E113 | 8843990 | Type 2 diabetic macular edema |
| Charlson Score | E113 | 8844347 | Type 2 diabetic cataract |
| Charlson Score | E113 | 8844537 | Proliferative type 2 diabetic retinopathy |
| Charlson Score | E113 | 8845072 | Type 2 diabetic maculopathy |
| Charlson Score | E113 | 8845078 | Type 2 diabetic ophthalmoplegia |
| Charlson Score | E113 | 8845082 | Type 2 diabetic iritis |
| Charlson Score | E113 | 8845093 | Type 2 diabetic central retinopathy |
| Charlson Score | E114 | 8830043 | Type 2 diabetic neuropathy |
| Charlson Score | E114 | 8830044 | Type 2 diabetic myopathies |
| Charlson Score | E114 | 8841693 | Type 2 diabetes mellitus with neurological complications |
| Charlson Score | E114 | 8845079 | Type 2 diabetic amyotrophy |
| Charlson Score | E114 | 8845084 | Type 2 diabetic neuropathic bladder |
| Charlson Score | E114 | 8845085 | Type 2 diabetic neuralgia |
| Charlson Score | E114 | 8845086 | Type 2 diabetic autonomic neuropathy |
| Charlson Score | E114 | 8845091 | Type 2 diabetic polyneuropathy |
| Charlson Score | E114 | 8845092 | Type 2 diabetic mononeuropathy |
| Charlson Score | E114 | 8845100 | Type 2 diabetic peripheral neuropathy |
| Charlson Score | E115 | 8841694 | Type 2 diabetes mellitus with peripheral circulatory complications |

| **Category** | **ICD10** | **Diagnosis Code** | **Diagnosis Name** |
| --- | --- | --- | --- |
| Charlson Score | E115 | 8843106 | Type 2 diabetic gangrene |
| Charlson Score | E115 | 8845075 | Type 2 diabetic ulcer |
| Charlson Score | E115 | 8845080 | Type 2 diabetic vascular disorders |
| Charlson Score | E115 | 8845095 | Type 2 diabetic arteriosclerosis |
| Charlson Score | E115 | 8845096 | Type 2 diabetic arterial occlusion |
| Charlson Score | E115 | 8845098 | Type 2 diabetic peripheral angiopathy |
| Charlson Score | E115 | 8845099 | Type 2 diabetic peripheral vascular disease |
| Charlson Score | E119 | 8841698 | Type 2 diabetes mellitus without complications |
| Charlson Score | E131 | 8843121 | Viral diabetes mellitus with ketoacidosis |
| Charlson Score | E131 | 8843376 | Pancreatogenous diabetes with ketoacidosis |
| Charlson Score | E131 | 8843389 | Steroid diabetes with ketoacidosis |
| Charlson Score | E131 | 8843449 | Secondary diabetes with ketoacidosis |
| Charlson Score | E131 | 8843620 | Drug-induced diabetes with ketoacidosis |
| Charlson Score | E131 | 8849587 | Hepatic diabetes mellitus with ketoacidosis |
| Charlson Score | E132 | 8843124 | Viral diabetes mellitus with renal complications |
| Charlson Score | E132 | 8843379 | Pancreatogenous diabetes with renal complications |
| Charlson Score | E132 | 8843392 | Steroid diabetes with renal complications |
| Charlson Score | E132 | 8843452 | Secondary diabetes with renal complications |
| Charlson Score | E132 | 8843623 | Drug-induced diabetes with renal complications |
| Charlson Score | E132 | 8849590 | Hepatic diabetes mellitus with renal complications |
| Charlson Score | E133 | 8843120 | Viral diabetes mellitus with ophthalmic complications |
| Charlson Score | E133 | 8843375 | Pancreatogenous diabetes with ophthalmic complications |
| Charlson Score | E133 | 8843388 | Steroid diabetes with ophthalmic complications |
| Charlson Score | E133 | 8843448 | Secondary diabetes with ophthalmic complications |
| Charlson Score | E133 | 8843619 | Drug-induced diabetes with ophthalmic complications |
| Charlson Score | E133 | 8849586 | Hepatic diabetes mellitus with ophthalmic complications |
| Charlson Score | E134 | 8843123 | Viral diabetes mellitus with neurological complications |
| Charlson Score | E134 | 8843378 | Pancreatogenous diabetes with neurological complications |
| Charlson Score | E134 | 8843391 | Steroid diabetes with neurological complications |
| Charlson Score | E134 | 8843451 | Secondary diabetes with neurological complications |
| Charlson Score | E134 | 8843622 | Drug-induced diabetes with neurological complications |
| Charlson Score | E134 | 8849589 | Hepatic diabetes mellitus with neurological complications |
| Charlson Score | E135 | 8843128 | Viral diabetes mellitus with peripheral circulatory complications |
| Charlson Score | E135 | 8843383 | Pancreatogenous diabetes with peripheral circulatory complications |

| **Category** | **ICD10** | **Diagnosis Code** | **Diagnosis Name** |
| --- | --- | --- | --- |
| Charlson Score | E135 | 8843396 | Steroid diabetes with peripheral circulatory complications |
| Charlson Score | E135 | 8843456 | Secondary diabetes with peripheral circulatory complications |
| Charlson Score | E135 | 8843627 | Drug-induced diabetes with peripheral circulatory complications |
| Charlson Score | E135 | 8849594 | Hepatic diabetes mellitus with peripheral circulatory complications |
| Charlson Score | E139 | 8843127 | Viral diabetes mellitus without complications |
| Charlson Score | E139 | 8843382 | Pancreatogenous diabetes without complications |
| Charlson Score | E139 | 8843395 | Steroid diabetes without complications |
| Charlson Score | E139 | 8843455 | Secondary diabetes without complications |
| Charlson Score | E139 | 8843626 | Drug-induced diabetes without complications |
| Charlson Score | E139 | 8849593 | Hepatic diabetes mellitus without complications |
| Charlson Score | E141 | 2501002 | Diabetic acidosis |
| Charlson Score | E141 | 2501003 | Diabetic acetonemia |
| Charlson Score | E141 | 2501005 | Diabetic ketoacidosis |
| Charlson Score | E141 | 8849181 | Diabetic ketosis |
| Charlson Score | E142 | 2503005 | Diabetic renal disease |
| Charlson Score | E142 | 2503007 | Diabetic kidney failure |
| Charlson Score | E142 | 8832747 | Kimmelsteel-Wilson syndrome |
| Charlson Score | E142 | 8838071 | Diabetic renal sclerosis |
| Charlson Score | E142 | 8850065 | Diabetic renal disease |
| Charlson Score | E143 | 2504004 | Diabetic iritis |
| Charlson Score | E143 | 2504005 | Central retinopathy diabetic |
| Charlson Score | E143 | 2504006 | Diabetic cataract |
| Charlson Score | E143 | 2504010 | Proliferative retinopathy diabetic |
| Charlson Score | E143 | 2504012 | Diabetic maculopathy |
| Charlson Score | E143 | 2504013 | Retinopathy diabetic |
| Charlson Score | E143 | 8838065 | Diabetic ophthalmoplegia |
| Charlson Score | E143 | 8844089 | Diabetic macular edema |
| Charlson Score | E144 | 2505011 | Diabetic neuralgia |
| Charlson Score | E144 | 2505018 | Diabetic peripheral neuropathy |
| Charlson Score | E144 | 2505021 | Diabetic amyotrophy |
| Charlson Score | E144 | 8838069 | Diabetic neuropathic bladder |
| Charlson Score | E144 | 8838070 | Diabetic autonomic neuropathy |
| Charlson Score | E144 | 8838074 | Diabetic polyneuropathy |
| Charlson Score | E144 | 8838075 | Diabetic mononeuropathy |
| Charlson Score | E144 | 8838078 | Diabetic neuropathy |
| Charlson Score | E144 | 8848634 | Diabetic foot lesions |
| Charlson Score | E144 | 8848768 | Diabetic neuropathic pain |
| Charlson Score | E145 | 2506006 | Diabetic gangrene |
| Charlson Score | E145 | 2506011 | Diabetic arterial occlusion |
| Charlson Score | E145 | 8838063 | Diabetic ulcer |
| Charlson Score | E145 | 8838066 | Diabetic vascular disorder |
| Charlson Score | E145 | 8838077 | Diabetic arteriosclerosis |
| Charlson Score | E145 | 8838079 | Diabetic peripheral angiopathy |
| Charlson Score | E145 | 8838080 | Diabetic peripheral vascular disease |

| **Category** | **ICD10** | **Diagnosis Code** | **Diagnosis Name** |
| --- | --- | --- | --- |
| Charlson Score | E145 | 8848632 | Diabetic foot gangrene |
| Charlson Score | E145 | 8848633 | Diabetic foot ulcers |
| Charlson Score | E149 | 8843439 | Diabetes mellitus without complications |
| Charlson Score | F010 | 8842565 | Vascular dementia of acute onset |
| Charlson Score | F011 | 8842608 | Multi-infarct dementia |
| Charlson Score | F011 | 8842626 | Cortical dementia |
| Charlson Score | F011 | 8847912 | CADASIL |
| Charlson Score | F011 | 8847913 | CARASIL |
| Charlson Score | F012 | 8842625 | Subcortical dementia |
| Charlson Score | F019 | 8842571 | Vascular dementia |
| Charlson Score | F051 | 8842619 | Delirium superimposed on dementia |
| Charlson Score | G450 | 4332006 | Vascular insufficiency of vertebral artery |
| Charlson Score | G450 | 4333004 | Vertebrobasilar insufficiency |
| Charlson Score | G450 | 4359041 | Basilar insufficiency |
| Charlson Score | G450 | 8837831 | Vertebral basilar artery insufficiency |
| Charlson Score | G451 | 8838303 | Internal carotid artery insufficiency |
| Charlson Score | G454 | 8830529 | Transient global amnesia |
| Charlson Score | G458 | 4241001 | Subclavian steal syndrome |
| Charlson Score | G458 | 4369015 | Impending stroke |
| Charlson Score | G459 | 4359007 | Transient ischaemic attack |
| Charlson Score | G459 | 4359038 | Cerebrovascular spasm |
| Charlson Score | G459 | 4359052 | Reversible ischemic neuronal damage |
| Charlson Score | G459 | 8838752 | Cerebral arteriospasm |
| Charlson Score | G459 | 8838752 | Cerebral arteriospasm |
| Charlson Score | G300 | 8842548 | Pre-senile dementia of the Alzheimer’s type |
| Charlson Score | G301 | 8842551 | Senile dementia of the Alzheimer’s type |
| Charlson Score | G308 | 8842550 | Atypical dementia of the Alzheimer’s type |
| Charlson Score | G308 | 8849974 | Mixed dementia |
| Charlson Score | G309 | 3310002 | Alzheimer’s disease |
| Charlson Score | G309 | 8842308 | Familial Alzheimer’s disease |
| Charlson Score | G309 | 8842549 | Dementia Alzheimer’s type |
| Charlson Score | G310 | 2901003 | Pick’s disease |
| Charlson Score | G310 | 8833284 | Circumscribed cerebral atrophy |
| Charlson Score | G310 | 8844891 | Frontotemporal dementia |
| Charlson Score | G310 | 8848596 | Frontotemporal degeneration |
| Charlson Score | G311 | 3312002 | Senile degeneration of brain |
| Charlson Score | G312 | 2919004 | Alcoholic encephalopathy |
| Charlson Score | G312 | 8830341 | Alcoholic cerebellar ataxia |
| Charlson Score | G312 | 8830351 | Alcoholic cerebellar degeneration |
| Charlson Score | G318 | 8840933 | Leigh syndrome |
| Charlson Score | G318 | 8842323 | Diffuse Lewy body disease |
| Charlson Score | G318 | 8842457 | Alpers disease |
| Charlson Score | G318 | 8845840 | Dementia with Lewy bodies |
| Charlson Score | G318 | 8848436 | Hereditary diffuse leukoencephalopathy with spheroids (HDLS) |
| Charlson Score | G318 | 8848644 | Infantile neuroaxonal dystrophy |
| Charlson Score | G318 | 8849489 | Progressive leukoencephalopathy with ovarian dysfunction |
| Charlson Score | G319 | 3319002 | Cerebral atrophy |

| **Category** | **ICD10** | **Diagnosis Code** | **Diagnosis Name** |
| --- | --- | --- | --- |
| Charlson Score | G319 | 3488007 | Cerebellar atrophy |
| Charlson Score | G319 | 8830187 | Subacute cerebellar degeneration |
| Charlson Score | G319 | 8834817 | Cerebellar degeneration |
| Charlson Score | G319 | 8835986 | Spinocerebellar degeneration |
| Charlson Score | G319 | 8849351 | Progressive leukoencephalopathy |
| Charlson Score | G810 | 3420002 | Flaccid hemiplegia |
| Charlson Score | G811 | 3421001 | Spastic hemiplegia |
| Charlson Score | G819 | 3429014 | Hemiplegia |
| Charlson Score | G819 | 3448011 | Alternating hemiplegia |
| Charlson Score | G819 | 4389027 | Post stroke hemiplegia |
| Charlson Score | G819 | 8830533 | Transient hemiplegia |
| Charlson Score | G819 | 8831201 | Inferior alternating hemiplegia |
| Charlson Score | G819 | 8835371 | Pure motor hemiplegia |
| Charlson Score | G819 | 8838253 | Ipsilateral hemiplegia |
| Charlson Score | G819 | 8838709 | Hemiplegia after cerebral infarction |
| Charlson Score | G819 | 8839785 | Hemiparesis |
| Charlson Score | G819 | 8849760 | Alternating hemiplegia of childhood |
| Charlson Score | G820 | 8834175 | Flaccid paraplegia |
| Charlson Score | G821 | 3441016 | Spastic paraplegia |
| Charlson Score | G821 | 3441022 | Paraplegia in flexion |
| Charlson Score | G822 | 3441015 | Paraplegia |
| Charlson Score | G822 | 8839784 | Paraparesis |
| Charlson Score | I210 | 8832400 | Acute myocardial infarction of anterolateral wall |
| Charlson Score | I210 | 8832401 | Acute myocardial infarction of anterior wall |
| Charlson Score | I210 | 8832402 | Acute apical myocardial infarction of anterior wall |
| Charlson Score | I210 | 8832403 | Acute anteroseptal myocardial infarction |
| Charlson Score | I210 | 8847004 | Acute extensive anterior myocardial infarction |
| Charlson Score | I211 | 8832297 | Acute myocardial infarction of inferoposterior wall |
| Charlson Score | I211 | 8832298 | Acute myocardial infarction of inferolateral wall |
| Charlson Score | I211 | 8832310 | Acute inferior myocardial infarction |
| Charlson Score | I211 | 8847013 | Rupture of chordae tendineae complicating acute myocardial infarction |
| Charlson Score | I211 | 8847057 | Papillary muscle rupture complicating acute myocardial infarction |
| Charlson Score | I211 | 8847058 | Papillary muscle dysfunction complicating acute myocardial infarction |
| Charlson Score | I212 | 8832326 | Acute myocardial infarction of basal-lateral wall |
| Charlson Score | I212 | 8832338 | Acute myocardial infarction of high-lateral wall |
| Charlson Score | I212 | 8832347 | Acute myocardial infarction of posterior wall |
| Charlson Score | I212 | 8832348 | Acute myocardial infarction of posteroseptal wall |
| Charlson Score | I212 | 8832356 | Acute myocardial infarction of posterobasal wall |
| Charlson Score | I212 | 8832357 | Acute myocardial infarction of posterolateral wall |
| Charlson Score | I212 | 8832387 | Acute myocardial infarction of apical lateral wall |
| Charlson Score | I212 | 8832404 | Acute myocardial infarction of lateral wall |
| Charlson Score | I212 | 8832409 | Acute septal myocardial infarction |

| **Category** | **ICD10** | **Diagnosis Code** | **Diagnosis Name** |
| --- | --- | --- | --- |
| Charlson Score | I212 | 8843284 | Acute right ventricular infarction |
| Charlson Score | I212 | 8847031 | Ventricular septal perforation complicating acute myocardial infarction |
| Charlson Score | I212 | 8847039 | Atrial septal perforation complicating acute myocardial infarction |
| Charlson Score | I213 | 8832316 | Acute transmural myocardial infarction |
| Charlson Score | I213 | 8847032 | Intraventricular thrombosis complicating acute myocardial infarction |
| Charlson Score | I213 | 8847036 | Apical thrombosis complicating acute myocardial infarction |
| Charlson Score | I213 | 8847038 | Cardiac rupture complicating acute myocardial infarction |
| Charlson Score | I213 | 8847040 | Atrial thrombosis complicating acute myocardial infarction |
| Charlson Score | I213 | 8847041 | Pericardial hematoma complicating acute myocardial infarction |
| Charlson Score | I214 | 8832389 | Acute subendocardial myocardial infarction |
| Charlson Score | I219 | 4109038 | Myocardial rupture |
| Charlson Score | I219 | 8832376 | Acute Myocardial infarction |
| Charlson Score | I219 | 8834919 | Myocardial infarction |
| Charlson Score | I219 | 8842460 | Right ventricular free wall rupture |
| Charlson Score | I219 | 8842490 | Left ventricular free wall rupture |
| Charlson Score | I219 | 8842693 | Coronary ostial atresia |
| Charlson Score | I219 | 8844146 | Coronary artery aneurysm rupture |
| Charlson Score | I219 | 8846988 | Acute ST segment elevation myocardial infarction |
| Charlson Score | I219 | 8847059 | Non-Q wave MI |
| Charlson Score | I219 | 8847060 | Non STEMI |
| Charlson Score | I220 | 8833965 | Relapsing myocardial infarction of anterior wall |
| Charlson Score | I220 | 8833970 | Relapsing septal myocardial infarction |
| Charlson Score | I221 | 8833961 | Relapsing myocardial infarction of inferior wall |
| Charlson Score | I228 | 8833962 | Relapsing myocardial infarction of posterior wall |
| Charlson Score | I228 | 8833966 | Relapsing myocardial infarction of lateral wall |
| Charlson Score | I229 | 8833963 | Relapsing myocardial infarction |
| Charlson Score | I252 | 8837801 | Old myocardial infarction of inferior wall |
| Charlson Score | I252 | 8837804 | Old myocardial infarction of posterior wall |
| Charlson Score | I252 | 8837807 | Old myocardial infarction |
| Charlson Score | I252 | 8837809 | Old anteroseptal myocardial infarction |
| Charlson Score | I252 | 8837810 | Old myocardial infarction of lateral wall |
| Charlson Score | I252 | 8847530 | Old myocardial infarction of anterior wall |
| Charlson Score | I500 | 4280002 | Cardiac edema |
| Charlson Score | I500 | 4280005 | Chronic congestive heart failure |
| Charlson Score | I500 | 4280011 | Right heart failure |
| Charlson Score | I500 | 8830796 | Congestive heart failure |
| Charlson Score | I500 | 8842461 | Right ventricular insufficiency |
| Charlson Score | I501 | 4281005 | Cardiac asthma |
| Charlson Score | I501 | 4281009 | Left heart failure |
| Charlson Score | I501 | 4281010 | Cardiac dyspnea |
| Charlson Score | I501 | 5140016 | Cardiogenic pulmonary edema |

| **Category** | **ICD10** | **Diagnosis Code** | **Diagnosis Name** |
| --- | --- | --- | --- |
| Charlson Score | I501 | 8834012 | Left ventricular insufficiency |
| Charlson Score | I509 | 4289005 | Acute heart failure |
| Charlson Score | I509 | 4289015 | Heart failure |
| Charlson Score | I509 | 4289018 | Chronic heart failure |
| Charlson Score | I509 | 8834931 | Myocardial failure |
| Charlson Score | I509 | 8841016 | Bi-ventricular failure |
| Charlson Score | I600 | 8842229 | Ruptured internal carotid artery dissection |
| Charlson Score | I600 | 8847449 | Subarachnoid hemorrhage due to ruptured IC- PC aneurysm |
| Charlson Score | I600 | 8847541 | Subarachnoid hemorrhage due to ruptured internal carotid artery aneurysm |
| Charlson Score | I600 | 8849043 | Subarachnoid hemorrhage due to ruptured internal carotid artery dissection |
| Charlson Score | I601 | 8837619 | Subarachnoid hemorrhage from middle cerebral artery |
| Charlson Score | I601 | 8847527 | Subarachnoid hemorrhage due to ruptured middle cerebral artery aneurysm |
| Charlson Score | I602 | 8836504 | Subarachnoid hemorrhage from anterior  communicating artery |
| Charlson Score | I602 | 8847505 | Subarachnoid hemorrhage due to ruptured anterior communicating artery aneurysm |
| Charlson Score | I603 | 8833433 | Subarachnoid hemorrhage from posterior communicating artery aneurysm |
| Charlson Score | I603 | 8847468 | Subarachnoid hemorrhage due to ruptured posterior communicating artery aneurysm |
| Charlson Score | I604 | 8838740 | Subarachnoid hemorrhage from basilar artery |
| Charlson Score | I604 | 8847545 | Subarachnoid hemorrhage due to ruptured basilar artery aneurysm |
| Charlson Score | I605 | 8842228 | Ruptured vertebral artery dissection |
| Charlson Score | I605 | 8847531 | Subarachnoid hemorrhage due to ruptured vertebral artery aneurysm |
| Charlson Score | I605 | 8849042 | Subarachnoid hemorrhage due to ruptured vertebral artery dissection |
| Charlson Score | I606 | 8846593 | Subarachnoid hemorrhage from posterior cerebral artery |
| Charlson Score | I606 | 8846750 | Subarachnoid hemorrhage from anterior cerebral artery |
| Charlson Score | I606 | 8847469 | Subarachnoid hemorrhage due to ruptured posterior cerebral artery aneurysm |
| Charlson Score | I606 | 8847506 | Subarachnoid hemorrhage due to ruptured  anterior cerebral artery aneurysm |
| Charlson Score | I607 | 8847536 | Subarachnoid hemorrhage due to ruptured intracranial aneurysm |
| Charlson Score | I608 | 8835797 | Meningorrhagia |
| Charlson Score | I608 | 8847895 | Cerebral arteriovenous malformations rupture |
| Charlson Score | I608 | 8847896 | Subarachnoid hemorrhage due to ruptured cerebral arteriovenous malformations |
| Charlson Score | I609 | 4309001 | Subarachnoid hemorrhage |
| Charlson Score | I609 | 4309005 | Idiopathic subarachnoid hemorrhage |

| **Category** | **ICD10** | **Diagnosis Code** | **Diagnosis Name** |
| --- | --- | --- | --- |
| Charlson Score | I609 | 8836339 | Congenital cerebral aneurysm rupture |
| Charlson Score | I609 | 8838751 | Cerebral aneurysm rupture |
| Charlson Score | I610 | 4310038 | Thalamic hemorrhage |
| Charlson Score | I610 | 4319027 | Subcortical hemorrhage |
| Charlson Score | I610 | 4319030 | Putamen hemorrhage |
| Charlson Score | I610 | 8847680 | Caudate nucleus hemorrhage |
| Charlson Score | I611 | 8839257 | Cortical intracerebral hemorrhage |
| Charlson Score | I613 | 4319013 | Brain stem hemorrhage |
| Charlson Score | I613 | 8841358 | Pontine hemorrhage |
| Charlson Score | I613 | 8845147 | Hematobulbia |
| Charlson Score | I614 | 4319006 | Cerebellar hemorrhage |
| Charlson Score | I615 | 4319018 | Intraventricular hemorrhage |
| Charlson Score | I615 | 4319032 | Intraventricular hematoma hemorrhage |
| Charlson Score | I616 | 8836998 | Multiple localized intracerebral hemorrhage |
| Charlson Score | I618 | 4320007 | Posterior cranial fossa hematoma |
| Charlson Score | I619 | 4319003 | Hypertensive intracerebral hemorrhage |
| Charlson Score | I619 | 4319009 | Idiopathic intracerebral hemorrhage |
| Charlson Score | I619 | 4319020 | Hematencephalon |
| Charlson Score | I619 | 8847897 | Cerebral hemorrhage due to ruptured cerebral arteriovenous malformations |
| Charlson Score | I620 | 4321006 | Infantile chronic subdural hematoma |
| Charlson Score | I620 | 4321008 | Chronic subdural hematoma |
| Charlson Score | I620 | 7670007 | Juvenile chronic subdural hematoma |
| Charlson Score | I620 | 8843499 | Non-traumatic acute subdural hemorrhage |
| Charlson Score | I621 | 8843500 | Non-traumatic acute extradural hemorrhage |
| Charlson Score | I629 | 8839202 | Non-traumatic intracranial hemorrhage |
| Charlson Score | I630 | 3448022 | Cestan’s syndrome |
| Charlson Score | I630 | 8838690 | Cerebral infarction due to thrombosis of precerebral arteries |
| Charlson Score | I630 | 8846410 | Cestan-Chenais syndrome |
| Charlson Score | I631 | 8838691 | Cerebral infarction due to embolism of precerebral arteries |
| Charlson Score | I632 | 8838692 | Cerebral infarction due to occlusion of precerebral arteries |
| Charlson Score | I633 | 8842255 | Atherothrombotic brain infarction |
| Charlson Score | I633 | 8846351 | Atherothrombotic cerebral infarction (acute stage) |
| Charlson Score | I633 | 8846352 | Atherothrombotic cerebral infarction (chronic stage) |
| Charlson Score | I633 | 8846384 | Thrombotic cerebellar infarction |
| Charlson Score | I633 | 8846385 | Thrombotic cerebral infarction |
| Charlson Score | I634 | 8842272 | Cardiogenic embolism |
| Charlson Score | I634 | 8846397 | Cardiogenic cerebellar infarction |
| Charlson Score | I634 | 8846412 | Embolic cerebellar infarction |
| Charlson Score | I634 | 8846413 | Embolic cerebellar infarction (acute stage) |
| Charlson Score | I634 | 8846414 | Embolic cerebellar infarction (chronic stage) |
| Charlson Score | I634 | 8846415 | Embolic cerebral infarction |
| Charlson Score | I634 | 8846416 | Embolic cerebral infarction (acute stage) |
| Charlson Score | I634 | 8846417 | Embolic cerebral infarction (chronic stage) |

| **Category** | **ICD10** | **Diagnosis Code** | **Diagnosis Name** |
| --- | --- | --- | --- |
| Charlson Score | I634 | 8847851 | Paradoxical cerebral embolism |
| Charlson Score | I635 | 4330012 | Top of the basilar syndrome |
| Charlson Score | I635 | 4341002 | Medullary infarction |
| Charlson Score | I635 | 4341005 | Pontine infarction |
| Charlson Score | I635 | 4341010 | Cerebellar infarction |
| Charlson Score | I635 | 4341027 | Brain stem infarction |
| Charlson Score | I635 | 4341052 | Penetrating branch infarction |
| Charlson Score | I635 | 4341053 | Cortical branch infarction |
| Charlson Score | I635 | 8838703 | Cerebrovascular obstructive cerebral infarction |
| Charlson Score | I635 | 8846357 | Medullary infarction (acute stage) |
| Charlson Score | I635 | 8846358 | Medullary infarction (chronic stage) |
| Charlson Score | I635 | 8846373 | Pontine infarction (acute stage) |
| Charlson Score | I635 | 8846374 | Pontine infarction (chronic stage) |
| Charlson Score | I635 | 8846419 | Multiple cerebellar infarction |
| Charlson Score | I635 | 8846436 | Brainstem stem infarction (acute stage) |
| Charlson Score | I635 | 8846437 | Brainstem stem infarction (chronic stage) |
| Charlson Score | I636 | 3259015 | Venous brain infarction |
| Charlson Score | I636 | 8835486 | Brain infarction due to venous thrombosis |
| Charlson Score | I638 | 4341018 | Multiple cerebral infarction |
| Charlson Score | I638 | 4341044 | Hemorrhagic cerebral infarction |
| Charlson Score | I638 | 4341045 | Asymptomatic cerebral infarction |
| Charlson Score | I638 | 4341049 | Lacunar infarction |
| Charlson Score | I638 | 4341056 | Watershed infarction |
| Charlson Score | I638 | 8846420 | Multiple lacunar infarction |
| Charlson Score | I638 | 8846438 | Cerebral infarction due to cerebral vasospasm |
| Charlson Score | I638 | 8846450 | Asymptomatic multiple cerebral infarction |
| Charlson Score | I638 | 8846451 | Asymptomatic lacunar infarction |
| Charlson Score | I638 | 8848096 | Cerebral infarction due to cerebral artery dissection |
| Charlson Score | I639 | 4349005 | Recurrent cerebral infarction |
| Charlson Score | I639 | 4369016 | Ischemic stroke |
| Charlson Score | I639 | 8838708 | Brain infarction |
| Charlson Score | I639 | 8838753 | Encephalomalacia |
| Charlson Score | I639 | 8846439 | Cerebral infarction (acute stage) |
| Charlson Score | I639 | 8846440 | Cerebral infarction (chronic stage) |
| Charlson Score | I639 | 8849423 | Cerebral infarction due to Trousseau’s syndrome |
| Charlson Score | I639 | 8849460 | Migrainous infarction |
| Charlson Score | I64 | 4369009 | Stroke |
| Charlson Score | I64 | 4369014 | Stroke in progression |
| Charlson Score | I64 | 8838704 | Cerebrovascular accident |
| Charlson Score | I650 | 3448001 | Avellis syndrome |
| Charlson Score | I650 | 8837826 | Vertebral artery stenosis |
| Charlson Score | I650 | 8837827 | Vertebral artery thrombosis |
| Charlson Score | I650 | 8837828 | Vertebral artery embolism |
| Charlson Score | I650 | 8837830 | Vertebral artery occlusion |
| Charlson Score | I651 | 8838741 | Basilar artery stenosis |
| Charlson Score | I651 | 8838742 | Basilar artery thrombosis |
| Charlson Score | I651 | 8838744 | Basilar artery apical embolism |

| **Category** | **ICD10** | **Diagnosis Code** | **Diagnosis Name** |
| --- | --- | --- | --- |
| Charlson Score | I651 | 8838745 | Basilar artery embolism |
| Charlson Score | I651 | 8838746 | Basilar artery occlusion |
| Charlson Score | I652 | 8832970 | Carotid atherosclerosis |
| Charlson Score | I652 | 8838298 | Internal carotid artery stenosis |
| Charlson Score | I652 | 8838300 | Internal carotid artery thrombosis |
| Charlson Score | I652 | 8838301 | Internal carotid artery embolism |
| Charlson Score | I652 | 8838304 | Internal carotid artery occlusion |
| Charlson Score | I652 | 8845880 | Ocular ischemic syndrome |
| Charlson Score | I653 | 8837832 | Vertebrobasilar artery stenosis |
| Charlson Score | I660 | 4338004 | Middle cerebral artery stenosis |
| Charlson Score | I660 | 8837620 | Middle cerebral artery thrombosis |
| Charlson Score | I660 | 8837621 | Middle cerebral artery syndrome |
| Charlson Score | I660 | 8837622 | Middle cerebral artery embolism |
| Charlson Score | I660 | 8837623 | Middle cerebral artery occlusion |
| Charlson Score | I661 | 8836542 | Anterior cerebral artery stenosis |
| Charlson Score | I661 | 8836543 | Anterior cerebral artery thrombosis |
| Charlson Score | I661 | 8836544 | Anterior cerebral artery syndrome |
| Charlson Score | I661 | 8836545 | Anterior cerebral artery embolism |
| Charlson Score | I661 | 8836546 | Anterior cerebral artery occlusion |
| Charlson Score | I662 | 3488005 | Thalamic pain |
| Charlson Score | I662 | 8833554 | Posterior cerebral artery stenosis |
| Charlson Score | I662 | 8833555 | Posterior cerebral artery thrombosis |
| Charlson Score | I662 | 8833556 | Posterior cerebral artery syndrome |
| Charlson Score | I662 | 8833557 | Posterior cerebral artery embolism |
| Charlson Score | I662 | 8833558 | Posterior cerebral artery occlusion |
| Charlson Score | I663 | 3489059 | Lateral medullary syndrome |
| Charlson Score | I663 | 4369001 | Wallenberg syndrome |
| Charlson Score | I663 | 8830921 | Medullary depression |
| Charlson Score | I663 | 8834809 | Cerebellar stroke syndrome |
| Charlson Score | I663 | 8834811 | Cerebellar artery stenosis |
| Charlson Score | I663 | 8834812 | Cerebellar artery thrombosis |
| Charlson Score | I663 | 8834813 | Cerebellar artery embolism |
| Charlson Score | I663 | 8834814 | Cerebellar artery obstruction |
| Charlson Score | I663 | 8844426 | Posterior inferior cerebellar artery obstruction |
| Charlson Score | I663 | 8844484 | Superior cerebellar artery occlusion |
| Charlson Score | I663 | 8844501 | Anterior inferior cerebellar artery occlusion |
| Charlson Score | I663 | 8844937 | Lateral medullary syndrome |
| Charlson Score | I668 | 3526001 | Claude syndrome |
| Charlson Score | I668 | 4341051 | Lacunar stroke |
| Charlson Score | I668 | 8844431 | Posterior communicating artery obstruction |
| Charlson Score | I668 | 8844509 | Anterior communicating artery obstruction |
| Charlson Score | I669 | 4341057 | Occlusive cerebrovascular disease |
| Charlson Score | I669 | 8838705 | Cerebral thrombosis |
| Charlson Score | I669 | 8838736 | Cerebral embolism |
| Charlson Score | I669 | 8838748 | Cerebral artery stenosis |
| Charlson Score | I669 | 8838750 | Cerebral artery occlusion |
| Charlson Score | I670 | 4373024 | Dissecting cerebral aneurysm |
| Charlson Score | I670 | 8844866 | Posterior inferior cerebellar artery dissection |
| Charlson Score | I670 | 8844868 | Posterior cerebral artery dissection |

| **Category** | **ICD10** | **Diagnosis Code** | **Diagnosis Name** |
| --- | --- | --- | --- |
| Charlson Score | I670 | 8844890 | Anterior cerebral artery dissection |
| Charlson Score | I670 | 8844900 | Middle cerebral artery dissection |
| Charlson Score | I671 | 4373003 | Anterior communicating artery aneurysm |
| Charlson Score | I671 | 4373005 | Multiple cerebral aneurysms |
| Charlson Score | I671 | 4373007 | Middle cerebral artery aneurysm |
| Charlson Score | I671 | 4373010 | Internal posterior communicating artery aneurysms |
| Charlson Score | I671 | 4373014 | Cerebral arteriovenous fistula |
| Charlson Score | I671 | 4373015 | Cerebral arterial aneurysm |
| Charlson Score | I671 | 4373020 | Posterior cerebral artery aneurysm |
| Charlson Score | I671 | 4373028 | Fusiform type cerebral aneurysm |
| Charlson Score | I671 | 8830739 | Arterial aneurysm of circle of Willis |
| Charlson Score | I671 | 8833591 | Acquired arteriovenous fistula |
| Charlson Score | I671 | 8838721 | Saccular aneurysm |
| Charlson Score | I671 | 8842858 | Unruptured cerebral aneurysm |
| Charlson Score | I671 | 8842952 | Anterior cerebral artery aneurysm |
| Charlson Score | I671 | 8843057 | Dural arteriovenous fistula |
| Charlson Score | I671 | 8844427 | Posterior inferior cerebellar artery aneurysm |
| Charlson Score | I671 | 8844432 | Posterior communicating artery aneurysm |
| Charlson Score | I671 | 8844485 | Superior cerebellar artery aneurysm |
| Charlson Score | I671 | 8844502 | Anterior inferior cerebellar artery aneurysm |
| Charlson Score | I671 | 8844561 | Internal carotid-posterior communicating artery aneurysms |
| Charlson Score | I671 | 9009002 | Aortic cerebral aneurysm |
| Charlson Score | I672 | 4370003 | Arteriosclerotic encephalopathy |
| Charlson Score | I672 | 8838749 | Cerebral arteriosclerosis |
| Charlson Score | I674 | 4372001 | Hypertensive malignant encephalopathy |
| Charlson Score | I674 | 4372002 | Hypertensive cerebral circulatory disorder |
| Charlson Score | I674 | 4372003 | Hypertensive encephalopathy |
| Charlson Score | I675 | 4375001 | Moyamoya disease |
| Charlson Score | I675 | 4375003 | Adult moyamoya disease |
| Charlson Score | I675 | 4375004 | Child moyamoya disease |
| Charlson Score | I676 | 3259002 | Cavernous sinus syndrome |
| Charlson Score | I676 | 8838722 | Cerebral venous thrombosis |
| Charlson Score | I676 | 8839253 | Cortical venous thrombosis |
| Charlson Score | I677 | 4374003 | Cerebral arteritis |
| Charlson Score | I677 | 8830740 | Willis periarteritis |
| Charlson Score | I677 | 8849299 | Primary central nervous system vasculitis |
| Charlson Score | I678 | 3489005 | Brain necrosis |
| Charlson Score | I678 | 3489032 | Foville syndrome |
| Charlson Score | I678 | 4370011 | Cerebral circulatory insufficiency |
| Charlson Score | I678 | 4371003 | Ischemic cerebrovascular disease |
| Charlson Score | I678 | 4379014 | Ischemic leukoencephalopathy |
| Charlson Score | I678 | 8838694 | Brain ischemia |
| Charlson Score | I678 | 8838770 | Cerebral capillary telangiectasia |
| Charlson Score | I678 | 8842527 | Cerebral circulatory failure |
| Charlson Score | I678 | 8847569 | Radiation necrosis |
| Charlson Score | I678 | 8849257 | Reversible cerebral vasoconstriction syndrome |

| **Category** | **ICD10** | **Diagnosis Code** | **Diagnosis Name** |
| --- | --- | --- | --- |
| Charlson Score | I678 | 8849583 | Reversible posterior leukoencephalopathy syndrome |
| Charlson Score | I679 | 3448002 | Weber syndrome |
| Charlson Score | I679 | 3448028 | Miyard-Gubrere syndrome |
| Charlson Score | I679 | 3489029 | Benedict syndrome |
| Charlson Score | I679 | 3489035 | Locked-in syndrome |
| Charlson Score | I679 | 4379006 | Cerebrovascular disorder |
| Charlson Score | I679 | 8835412 | Superior alternating hemiplegia |
| Charlson Score | I679 | 8838688 | Brain stem stroke syndrome |
| Charlson Score | I690 | 4389001 | Sequelae of subarachnoid hemorrhage |
| Charlson Score | I691 | 4389017 | Sequelae of intracerebral hemorrhage |
| Charlson Score | I693 | 4341026 | Old cerebral infarction |
| Charlson Score | I693 | 4389014 | Late effects of cerebral infarction |
| Charlson Score | I693 | 8834808 | Late effects of cerebellar infarction |
| Charlson Score | I693 | 8846424 | Old atherothrombotic cerebral infarction |
| Charlson Score | I693 | 8846425 | Old medullary infarction |
| Charlson Score | I693 | 8846426 | Old pontine infarction |
| Charlson Score | I693 | 8846427 | Old cerebellar infarction |
| Charlson Score | I693 | 8846428 | Old embolic cerebral infarction |
| Charlson Score | I693 | 8846429 | Old multiple cerebral infarction |
| Charlson Score | I693 | 8846430 | Old brain stem infarction |
| Charlson Score | I693 | 8846431 | Old lacunar infarction |
| Charlson Score | I694 | 4389024 | Sequelae of stroke |
| Charlson Score | I710 | 4410003 | Dissecting aortic aneurysm |
| Charlson Score | I710 | 4411007 | Acute aortic dissection |
| Charlson Score | I710 | 8831072 | Dissecting thoracic aortic aneurysm |
| Charlson Score | I710 | 8831077 | Dissecting aortic aneurysm (DeBakey type I) |
| Charlson Score | I710 | 8831078 | Dissecting aortic aneurysm (DeBakey type II) |
| Charlson Score | I710 | 8831079 | Dissecting aortic aneurysm (DeBakey type IIIa) |
| Charlson Score | I710 | 8831080 | Dissecting aortic aneurysm (DeBakey type IIIb) |
| Charlson Score | I710 | 8842465 | Dissecting aortic aneurysm (Stanford A) |
| Charlson Score | I710 | 8842466 | Dissecting aortic aneurysm (Stanford B) |
| Charlson Score | I710 | 8842477 | Acute aortic dissection (DeBakey I) |
| Charlson Score | I710 | 8842478 | Acute aortic dissection (DeBakey II) |
| Charlson Score | I710 | 8842479 | Acute aortic dissection (DeBakey IIIa) |
| Charlson Score | I710 | 8842480 | Acute aortic dissection (DeBakey IIIb) |
| Charlson Score | I710 | 8842481 | Acute aortic dissection (Stanford A) |
| Charlson Score | I710 | 8842482 | Acute aortic dissection (Stanford B) |
| Charlson Score | I710 | 8842515 | Early thrombosed aortic dissection |
| Charlson Score | I710 | 8844922 | Chronic aortic dissection |
| Charlson Score | I711 | 4411002 | Thoracic aortic aneurysm rupture |
| Charlson Score | I711 | 8838989 | Ruptured thoracic aortic aneurysm |
| Charlson Score | I712 | 4412001 | Thoracic aortic aneurysm |
| Charlson Score | I712 | 4412005 | Impending rupture of thoracic aortic aneurysm |
| Charlson Score | I712 | 8835407 | Aneurysms of the ascending thoracic aorta |
| Charlson Score | I712 | 8842685 | Aneurysms of the descending thoracic aorta |
| Charlson Score | I712 | 8842700 | Aneurysm of arch of aorta |
| Charlson Score | I712 | 8844859 | Dilatation of thoracic aorta |
| Charlson Score | I712 | 8845236 | Infective thoracic aortic aneurysm |

| **Category** | **ICD10** | **Diagnosis Code** | **Diagnosis Name** |
| --- | --- | --- | --- |
| Charlson Score | I712 | 8846404 | Ascending aortic dilatation |
| Charlson Score | I713 | 4413002 | Abdominal aortic aneurysm rupture |
| Charlson Score | I713 | 8838991 | Ruptured abdominal aortic aneurysm |
| Charlson Score | I714 | 4414002 | Abdominal aortic aneurysm |
| Charlson Score | I714 | 4414005 | Impending rupture of abdominal aortic aneurysm |
| Charlson Score | I714 | 8844916 | Dilatation of abdominal aortic aneurysm |
| Charlson Score | I714 | 8845238 | Infective abdominal aortic aneurysm |
| Charlson Score | I715 | 8832571 | Thoracoabdominal aortic aneurysm rupture |
| Charlson Score | I715 | 8838988 | Ruptured thoracoabdominal aortic aneurysm |
| Charlson Score | I716 | 8832570 | Thoracoabdominal aortic aneurysm |
| Charlson Score | I718 | 8837397 | Aortic rupture |
| Charlson Score | I719 | 4416003 | Aortic aneurysm |
| Charlson Score | I719 | 8837386 | Aortic ectasia |
| Charlson Score | I739 | 4439001 | Vascular disease of lower limbs |
| Charlson Score | I739 | 4439002 | Peripheral circulatory disturbance of lower limbs |
| Charlson Score | I739 | 4439011 | Arteriosclerotic intermittent claudication |
| Charlson Score | I739 | 4439020 | Peripheral circulatory disturbance |
| Charlson Score | I739 | 8831507 | Intermittent claudication |
| Charlson Score | I739 | 8838278 | Spasm of artery |
| Charlson Score | I739 | 8840260 | Peripheral vasospasm |
| Charlson Score | I739 | 8847576 | Peripheral arterial disease |
| Charlson Score | J40 | 4900009 | Bronchitis |
| Charlson Score | J40 | 8830902 | Swallowing bronchitis |
| Charlson Score | J40 | 8831363 | Catarrhal bronchitis |
| Charlson Score | J40 | 8832147 | Tracheobronchitis |
| Charlson Score | J40 | 8837799 | Hypostatic bronchitis |
| Charlson Score | J40 | 8839537 | Diffuse bronchitis |
| Charlson Score | J40 | 8839605 | Fibrin bronchitis |
| Charlson Score | J40 | 8840227 | Membranous bronchitis |
| Charlson Score | J410 | 8832233 | Smoker’s bronchitis |
| Charlson Score | J410 | 8837134 | Simple chronic bronchitis |
| Charlson Score | J411 | 8838670 | Mucopurulent chronic bronchitis |
| Charlson Score | J411 | 8840311 | Chronic suppurative bronchitis |
| Charlson Score | J42 | 4919002 | Chronic bronchitis |
| Charlson Score | J42 | 8840330 | Chronic tracheitis |
| Charlson Score | J42 | 8840331 | Chronic tracheobronchitis |
| Charlson Score | J42 | 8840333 | Chronic bronchorrhea |
| Charlson Score | J42 | 8841221 | Senile bronchitis |
| Charlson Score | J430 | 8830557 | Unilateral pulmonary emphysema |
| Charlson Score | J430 | 8840234 | MacLeod syndrome |
| Charlson Score | J431 | 8839011 | Panlobular emphysema |
| Charlson Score | J432 | 8837582 | Centrilobular emphysema |
| Charlson Score | J439 | 4920017 | Emphysema |
| Charlson Score | J439 | 4920020 | Chronic pulmonary emphysema |
| Charlson Score | J439 | 4920021 | Senile emphysema |
| Charlson Score | J439 | 8830448 | Atrophic emphysema |
| Charlson Score | J439 | 8832218 | Emphysematous lung cyst |
| Charlson Score | J439 | 8832694 | Giant emphysematous bullae |

| **Category** | **ICD10** | **Diagnosis Code** | **Diagnosis Name** |
| --- | --- | --- | --- |
| Charlson Score | J439 | 8834830 | Interlobular emphysema |
| Charlson Score | J439 | 8838902 | Alveolar emphysema |
| Charlson Score | J439 | 8839846 | Bullous emphysema |
| Charlson Score | J439 | 8839950 | Obstructive emphysema |
| Charlson Score | J440 | 8849256 | Chronic obstructive pulmonary disease with acute lower respiratory infection |
| Charlson Score | J441 | 8849212 | Chronic obstructive pulmonary disease with acute exacerbation |
| Charlson Score | J448 | 4912001 | Diffuse panbronchiolitis |
| Charlson Score | J448 | 4912003 | Obstructive bronchitis |
| Charlson Score | J448 | 4912004 | Bronchiolitis obliterans |
| Charlson Score | J448 | 8849096 | Emphysematous chronic obstructive bronchitis |
| Charlson Score | J448 | 8849097 | Emphysematous chronic obstructive pulmonary disease |
| Charlson Score | J448 | 8849192 | Nonemphysematous chronic obstructive pulmonary disease |
| Charlson Score | J449 | 8840399 | Chronic obstructive pulmonary disease |
| Charlson Score | J449 | 8850253 | Asthma and COPD overlap |
| Charlson Score | J450 | 4930001 | Atopic asthma |
| Charlson Score | J450 | 4930002 | Extrinsic asthma |
| Charlson Score | J450 | 4930005 | Infantile asthma |
| Charlson Score | J450 | 4930006 | Occupational asthma |
| Charlson Score | J450 | 4939003 | Allergic bronchitis |
| Charlson Score | J450 | 4939004 | Allergic asthma |
| Charlson Score | J450 | 8834797 | Infantile asthmatic bronchitis |
| Charlson Score | J450 | 8847408 | Infantile asthma |
| Charlson Score | J451 | 4939016 | Psychologic asthma |
| Charlson Score | J451 | 4939039 | Non-atopic asthma |
| Charlson Score | J451 | 8830247 | Aspirin asthma |
| Charlson Score | J451 | 8831609 | Infectious bronchial asthma |
| Charlson Score | J458 | 8833884 | Mixed asthma |
| Charlson Score | J459 | 4939008 | Bronchial asthma |
| Charlson Score | J459 | 4939022 | Asthmatic bronchitis |
| Charlson Score | J459 | 4939037 | Refractory asthma |
| Charlson Score | J459 | 4939038 | Nocturnal asthma |
| Charlson Score | J459 | 8833454 | Bronchial asthma with eosinophilia |
| Charlson Score | J459 | 8840365 | Chronic asthmatic bronchitis |
| Charlson Score | J459 | 8841641 | Exercise-induced asthma |
| Charlson Score | J459 | 8844994 | Steroid-dependent asthma |
| Charlson Score | J459 | 8846176 | Cough variant asthma |
| Charlson Score | J46 | 4939010 | Status asthmaticus |
| Charlson Score | J46 | 4939012 | Asthmatic attack |
| Charlson Score | J47 | 4950002 | Bronchiectasis |
| Charlson Score | J47 | 8830923 | Cylindrical bronchiectasis |
| Charlson Score | J47 | 8831457 | Lower lobe bronchiectasis |
| Charlson Score | J47 | 8833273 | Localized bronchiectasis |
| Charlson Score | J47 | 8833931 | Bronchiolectasis |
| Charlson Score | J47 | 8838720 | Cystic bronchiectasis |
| Charlson Score | J47 | 8839538 | Diffuse bronchiectasis |

| **Category** | **ICD10** | **Diagnosis Code** | **Diagnosis Name** |
| --- | --- | --- | --- |
| Charlson Score | J47 | 8840332 | Chronic bronchiectasis |
| Charlson Score | J60 | 8837111 | Coal worker’s pneumoconiosis |
| Charlson Score | J61 | 5019002 | Asbestosis |
| Charlson Score | J628 | 8832978 | Silicosis |
| Charlson Score | J628 | 8836028 | Chalicicosis |
| Charlson Score | J630 | 8830379 | Aluminosis (of lung) |
| Charlson Score | J631 | 8840118 | Bauxite fibrosis (of lung) |
| Charlson Score | J632 | 8840034 | Berylliosis |
| Charlson Score | J632 | 8840035 | Beryllium granulomas |
| Charlson Score | J633 | 8833754 | Graphite fibrosis (of lung) |
| Charlson Score | J634 | 8837915 | Siderosis |
| Charlson Score | J635 | 8834502 | Stannosis |
| Charlson Score | J64 | 8835641 | Pneumoconiosis |
| Charlson Score | J65 | 8832977 | Silicotuberculosis |
| Charlson Score | J65 | 8835640 | Pneumoconiosis |
| Charlson Score | J660 | 8840567 | Byssinosis |
| Charlson Score | J661 | 8830223 | Flax-dresser disease |
| Charlson Score | J670 | 8838756 | Farmer’s lung |
| Charlson Score | J671 | 8834022 | Bagassosis |
| Charlson Score | J672 | 8838218 | Bird fancier’s lung |
| Charlson Score | J673 | 8833866 | Suberosis |
| Charlson Score | J674 | 8839093 | Malt worker’s lung |
| Charlson Score | J675 | 8832249 | Mushroom worker’s lung |
| Charlson Score | J676 | 8831092 | Maple bark stripper’s lung |
| Charlson Score | J677 | 8831222 | Humidifier lung |
| Charlson Score | J677 | 8832788 | Air conditioner lung |
| Charlson Score | J678 | 4959009 | Summer-type hypersensitivity pneumonitis |
| Charlson Score | J678 | 8832779 | Fishmeal worker’s lung |
| Charlson Score | J678 | 8833029 | Furrier’s lung |
| Charlson Score | J678 | 8833323 | Coffee worker’s lung |
| Charlson Score | J678 | 8836033 | Sequoiasis |
| Charlson Score | J678 | 8837458 | Cheese-washer’s lung |
| Charlson Score | J679 | 4959008 | Hypersensitivity pneumonitis |
| Charlson Score | K250 | 5319011 | Acute gastric ulcer with hemorrhage |
| Charlson Score | K250 | 5350009 | Acute gastric mucosal lesion |
| Charlson Score | K250 | 8847768 | Postoperative gastric ulcer |
| Charlson Score | K250 | 8847779 | Stress ulcer |
| Charlson Score | K250 | 8847799 | Dieulafoy’s lesion |
| Charlson Score | K251 | 8832277 | Acute gastric ulcer with perforation |
| Charlson Score | K252 | 8845122 | Acute gastric ulcer with hemorrhage and perforation |
| Charlson Score | K253 | 5313001 | Acute gastric ulcer |
| Charlson Score | K254 | 8834632 | Hemorrhagic gastric ulcer |
| Charlson Score | K254 | 8847788 | Multiple hemorrhagic gastric ulcer |
| Charlson Score | K254 | 9620001 | Steroid ulcer |
| Charlson Score | K255 | 5310002 | Ulcer stomach with perforation |
| Charlson Score | K255 | 8830483 | Stomach perforation |
| Charlson Score | K255 | 8835734 | Steroid ulcer perforation |
| Charlson Score | K255 | 8847782 | Penetrating gastric ulcer |

| **Category** | **ICD10** | **Diagnosis Code** | **Diagnosis Name** |
| --- | --- | --- | --- |
| Charlson Score | K256 | 8845130 | Ulcer stomach with perf and hem |
| Charlson Score | K257 | 5317003 | Chronic gastric ulcer |
| Charlson Score | K257 | 8830419 | Gastric ulcer scar |
| Charlson Score | K257 | 8833960 | Reactivated gastric ulcer |
| Charlson Score | K257 | 8847819 | Active stage of chronic gastric ulcer |
| Charlson Score | K259 | 5310001 | Gastric erosion |
| Charlson Score | K259 | 5317002 | Intractable gastric ulcer |
| Charlson Score | K259 | 5319009 | Gastric ulcer |
| Charlson Score | K259 | 8834146 | Residual gastric ulcer |
| Charlson Score | K259 | 8842157 | NSAIDs-associated gastric ulcer |
| Charlson Score | K259 | 8847787 | Multiple gastric ulcers |
| Charlson Score | K259 | 8847823 | Drug-induced gastric ulcer |
| Charlson Score | K260 | 8845123 | Acute duodenal ulcer with hemorrhage |
| Charlson Score | K260 | 8847773 | Postoperative duodenal ulcer |
| Charlson Score | K260 | 8847780 | Stress-induced duodenal ulcer |
| Charlson Score | K261 | 8845126 | Acute duodenal ulcer with perforation |
| Charlson Score | K262 | 8845124 | Acute duodenal ulcer with hemorrhage and perforation |
| Charlson Score | K263 | 8845125 | Acute duodenal ulcer |
| Charlson Score | K264 | 8834641 | Duodenal ulcer bleeding |
| Charlson Score | K265 | 5325003 | Perforated duodenal ulcer |
| Charlson Score | K265 | 5329012 | Penetrating duodenal ulcer |
| Charlson Score | K265 | 8835296 | Perforation duodenal |
| Charlson Score | K266 | 8845131 | Duodenal ulcer with hemorrhage and perforation |
| Charlson Score | K267 | 8835291 | Duodenal ulcer scar |
| Charlson Score | K267 | 8845143 | Chronic duodenal ulcer |
| Charlson Score | K267 | 8847756 | Ulcer duodenal reactivated |
| Charlson Score | K267 | 8847820 | Active stage of chronic duodenal ulcer |
| Charlson Score | K269 | 5329002 | Duodenal ulcer |
| Charlson Score | K269 | 5329003 | Duodenal erosions |
| Charlson Score | K269 | 8842158 | NSAID-induced duodenal ulcer |
| Charlson Score | K269 | 8842506 | Postbulbar duodenal ulcer |
| Charlson Score | K269 | 8847789 | Multiple duodenal ulcer |
| Charlson Score | K269 | 8847806 | Intractable duodenal ulcer |
| Charlson Score | K284 | 8847762 | Hemorrhagic anastomotic ulcer |
| Charlson Score | K285 | 8847781 | Perforated anastomotic ulcer |
| Charlson Score | K287 | 8847817 | Anastomotic ulcer scar |
| Charlson Score | K289 | 5340003 | Anastomotic ulcer |
| Charlson Score | K289 | 8847807 | Intractable anastomotic ulcer |
| Charlson Score | K702 | 8846239 | Alcoholic hepatic fibrosis |
| Charlson Score | K703 | 5712001 | Alcoholic cirrhosis |
| Charlson Score | K703 | 8846331 | Decompensated alcoholic cirrhosis |
| Charlson Score | K703 | 8846466 | Oesophageal varices associated with alcoholic cirrhosis |
| Charlson Score | K703 | 8849244 | Oesophageal varices bleeding associated with alcoholic cirrhosis |
| Charlson Score | K717 | 8849218 | Drug-induced cirrhosis |
| Charlson Score | K721 | 8840321 | Chronic hepatic failure |

| **Category** | **ICD10** | **Diagnosis Code** | **Diagnosis Name** |
| --- | --- | --- | --- |
| Charlson Score | K729 | 5722002 | Hepatic coma |
| Charlson Score | K729 | 5722003 | Hepatic encephalopathy |
| Charlson Score | K729 | 5738005 | Hepatocellular jaundice |
| Charlson Score | K729 | 5738014 | Hepatic failure |
| Charlson Score | K729 | 8831477 | Hepatic atrophy |
| Charlson Score | K729 | 8831481 | Hepatic necrosis |
| Charlson Score | K730 | 8840360 | Chronic persistent hepatitis |
| Charlson Score | K732 | 5714010 | Chronic active hepatitis |
| Charlson Score | K738 | 5714007 | Chronic inactive hepatitis |
| Charlson Score | K739 | 5714005 | Chronic hepatitis |
| Charlson Score | K739 | 5714006 | Exacerbation of chronic hepatitis |
| Charlson Score | K739 | 5733010 | Prolonged hepatitis |
| Charlson Score | K740 | 8831607 | Hepatic fibrosis |
| Charlson Score | K743 | 5716001 | Primary biliary cirrhosis |
| Charlson Score | K743 | 8834520 | Charcot’s cirrhosis |
| Charlson Score | K743 | 8837208 | Unilobar cirrhosis |
| Charlson Score | K743 | 8838207 | Todd’s cirrhosis |
| Charlson Score | K743 | 8840388 | Chronic non-suppurative destructive cholangitis |
| Charlson Score | K743 | 8845947 | Symptomatic primary biliary cirrhosis |
| Charlson Score | K743 | 8846062 | Asymptomatic primary biliary cirrhosis |
| Charlson Score | K743 | 8849023 | Primary biliary cholangitis |
| Charlson Score | K743 | 8849027 | Symptomatic primary biliary cholangitis |
| Charlson Score | K743 | 8849051 | Asymptomatic primary biliary cholangitis |
| Charlson Score | K743 | 8849297 | Oesophageal varices associated with primary biliary cholangitis |
| Charlson Score | K743 | 8849298 | Oesophageal varices bleeding associated with primary biliary cholangitis |
| Charlson Score | K744 | 5716003 | Secondary biliary cirrhosis |
| Charlson Score | K744 | 5716004 | Obstructive cirrhosis |
| Charlson Score | K745 | 5716002 | Biliary cirrhosis |
| Charlson Score | K745 | 8837113 | Cholangetic cirrhosis |
| Charlson Score | K746 | 5715012 | Decompensated cirrhosis |
| Charlson Score | K746 | 5715013 | Portal cirrhosis |
| Charlson Score | K746 | 5715025 | Compensated cirrhosis |
| Charlson Score | K746 | 5715027 | Postnecrotic cirrhosis |
| Charlson Score | K746 | 8830442 | Atrophic cirrhosis |
| Charlson Score | K746 | 8830819 | Nutritional cirrhosis |
| Charlson Score | K746 | 8831482 | Posthepatitis cirrhosis |
| Charlson Score | K746 | 8831512 | Hepatic cirrhosis |
| Charlson Score | K746 | 8833121 | Nodular cirrhosis |
| Charlson Score | K746 | 8833880 | Mixed cirrhosis |
| Charlson Score | K746 | 8834697 | Micronodular cirrhosis |
| Charlson Score | K746 | 8837269 | Macronodular cirrhosis |
| Charlson Score | K746 | 8837557 | Septal cirrhosis |
| Charlson Score | K746 | 8838175 | Cryptogenic cirrhosis |
| Charlson Score | K746 | 8840692 | Periportal cirrhosis |
| Charlson Score | K746 | 8846291 | Autoimmune cirrhosis |
| Charlson Score | K746 | 8846517 | Oesophageal varices associated with cirrhosis |

| **Category** | **ICD10** | **Diagnosis Code** | **Diagnosis Name** |
| --- | --- | --- | --- |
| Charlson Score | K746 | 8849260 | Oesophageal varices bleeding associated with cirrhosis |
| Charlson Score | K766 | 4598008 | Portal vein ectasia |
| Charlson Score | K766 | 5723004 | Idiopathic portal hypertension |
| Charlson Score | K766 | 8832839 | Cruveilhier-Baumgarten syndrome |
| Charlson Score | K766 | 8840687 | Portal hypertension |
| Charlson Score | K766 | 8843101 | Portal hypertensive gastropathy |
| Charlson Score | K766 | 8845506 | Portal hypertensive gastroenteropathy |
| Charlson Score | K766 | 8845507 | Portal hypertensive enteropathy |
| Charlson Score | K767 | 8831588 | Hepatorenal syndrome |
| Charlson Score | M0500 | 8839621 | Felty’s syndrome |
| Charlson Score | M0510 | 8831446 | Caplan’s syndrome |
| Charlson Score | M0510 | 8840935 | Rheumatic interstitial pneumonitis |
| Charlson Score | M0510 | 8840951 | Rheumatic lung disease |
| Charlson Score | M0510 | 8847737 | Rheumatoid arthritis-associated interstitial pneumonia |
| Charlson Score | M0520 | 8840937 | Rheumatic vasculitis |
| Charlson Score | M0520 | 8849490 | Rheumatoid vasculitis |
| Charlson Score | M053 | 8847586 | Rheumatic pleuritis |
| Charlson Score | M0530 | 7148003 | Malignant rheumatoid arthritis |
| Charlson Score | M0530 | 8842100 | Rheumatic myopathy |
| Charlson Score | M0530 | 8842101 | Rheumatic carditis |
| Charlson Score | M0530 | 8842102 | Rheumatic myocarditis |
| Charlson Score | M0530 | 8842103 | Rheumatic pericarditis |
| Charlson Score | M0580 | 8850165 | Seropositive rheumatoid arthritis without complications |
| Charlson Score | M0581 | 8850163 | Seropositive rheumatoid arthritis in shoulder joint without complications |
| Charlson Score | M0582 | 8850175 | Seropositive rheumatoid arthritis in elbow joint without complications |
| Charlson Score | M0583 | 8850172 | Seropositive rheumatoid arthritis in wrist joint without complications |
| Charlson Score | M0584 | 8850169 | Seropositive rheumatoid arthritis in finger joint without complications |
| Charlson Score | M0585 | 8850168 | Seropositive rheumatoid arthritis in hip joint  without complications |
| Charlson Score | M0586 | 8850171 | Seropositive rheumatoid arthritis in knee joint without complications |
| Charlson Score | M0587 | 8850170 | Seropositive rheumatoid arthritis in toe joint without complications |
| Charlson Score | M0587 | 8850174 | Seropositive rheumatoid arthritis in ankle joint without complications |
| Charlson Score | M0588 | 8850164 | Seropositive rheumatoid arthritis in temporomandibular joint without complications |
| Charlson Score | M0588 | 8850166 | Seropositive rheumatoid arthritis in thoracic vertebra without complications |
| Charlson Score | M0588 | 8850167 | Seropositive rheumatoid arthritis in cervical  vertebra without complications |

| **Category** | **ICD10** | **Diagnosis Code** | **Diagnosis Name** |
| --- | --- | --- | --- |
| Charlson Score | M0588 | 8850173 | Seropositive rheumatoid arthritis in spine without complications |
| Charlson Score | M0588 | 8850176 | Seropositive rheumatoid arthritis in lumbar vertebra without complications |
| Charlson Score | M0590 | 8842104 | Seropositive rheumatoid arthritis |
| Charlson Score | M0591 | 8849616 | Seropositive rheumatoid arthritis in shoulder joint |
| Charlson Score | M0592 | 8849627 | Seropositive rheumatoid arthritis in elbow joint |
| Charlson Score | M0593 | 8849624 | Seropositive rheumatoid arthritis in wrist joint |
| Charlson Score | M0594 | 8849621 | Seropositive rheumatoid arthritis in finger joint |
| Charlson Score | M0595 | 8849620 | Seropositive rheumatoid arthritis in hip joint |
| Charlson Score | M0596 | 8849623 | Seropositive rheumatoid arthritis in knee joint |
| Charlson Score | M0597 | 8849622 | Seropositive rheumatoid arthritis in toe joint |
| Charlson Score | M0597 | 8849626 | Seropositive rheumatoid arthritis in ankle joint |
| Charlson Score | M0598 | 8849617 | Seropositive rheumatoid arthritis in temporomandibular joint |
| Charlson Score | M0598 | 8849618 | Seropositive rheumatoid arthritis in thoracic vertebra |
| Charlson Score | M0598 | 8849619 | Seropositive rheumatoid arthritis in cervical vertebra |
| Charlson Score | M0598 | 8849625 | Seropositive rheumatoid arthritis in spine |
| Charlson Score | M0598 | 8849628 | Seropositive rheumatoid arthritis in lumbar vertebra |
| Charlson Score | M0600 | 8842105 | Seronegative rheumatoid arthritis |
| Charlson Score | M0600 | 8844120 | RS3PE syndrome |
| Charlson Score | M0601 | 8849603 | Seronegative rheumatoid arthritis in shoulder joint |
| Charlson Score | M0602 | 8849614 | Seronegative rheumatoid arthritis in elbow joint |
| Charlson Score | M0603 | 8849611 | Seronegative rheumatoid arthritis in wrist joint |
| Charlson Score | M0604 | 8849608 | Seronegative rheumatoid arthritis in finger joint |
| Charlson Score | M0605 | 8849607 | Seronegative rheumatoid arthritis in hip joint |
| Charlson Score | M0606 | 8849610 | Seronegative rheumatoid arthritis in knee joint |
| Charlson Score | M0607 | 8849609 | Seronegative rheumatoid arthritis in toe joint |
| Charlson Score | M0607 | 8849613 | Seronegative rheumatoid arthritis in ankle joint |
| Charlson Score | M0608 | 8849604 | Seronegative rheumatoid arthritis in temporomandibular joint |
| Charlson Score | M0608 | 8849605 | Seronegative rheumatoid arthritis in thoracic vertebra |
| Charlson Score | M0608 | 8849606 | Seronegative rheumatoid arthritis in cervical vertebra |
| Charlson Score | M0608 | 8849612 | Seronegative rheumatoid arthritis in spine |
| Charlson Score | M0608 | 8849615 | Seronegative rheumatoid arthritis in lumbar vertebra |
| Charlson Score | M0630 | 8840952 | Rheumatic subcutaneous nodule |
| Charlson Score | M0690 | 7140010 | Rheumatoid arthritis |
| Charlson Score | M0690 | 7140016 | Multiple rheumatoid arthritis |
| Charlson Score | M0690 | 8842106 | Rheumatoid arthritis |
| Charlson Score | M0691 | 8842152 | Rheumatoid arthritis in shoulder joint |
| Charlson Score | M0692 | 8842153 | Rheumatoid arthritis in elbow joint |

| **Category** | **ICD10** | **Diagnosis Code** | **Diagnosis Name** |
| --- | --- | --- | --- |
| Charlson Score | M0693 | 8842136 | Rheumatoid arthritis in wrist joint |
| Charlson Score | M0694 | 8842137 | Rheumatoid arthritis in finger joint |
| Charlson Score | M0695 | 8842140 | Rheumatoid arthritis in hip joint |
| Charlson Score | M0696 | 8842138 | Rheumatoid arthritis in knee joint |
| Charlson Score | M0697 | 8842139 | Rheumatoid arthritis in ankle joint |
| Charlson Score | M0697 | 8842141 | Rheumatoid arthritis in toe joint |
| Charlson Score | M0698 | 8844635 | Rheumatoid arthritis in thoracic vertebra |
| Charlson Score | M0698 | 8844636 | Rheumatoid arthritis in cervical vertebra |
| Charlson Score | M0698 | 8844637 | Rheumatoid arthritis in spine |
| Charlson Score | M0698 | 8844638 | Rheumatoid arthritis in lumbar vertebra |
| Charlson Score | M0698 | 8846107 | Rheumatoid arthritis in temporomandibular joint |
| Charlson Score | M320 | 7100031 | Drug-induced lupus |
| Charlson Score | M321 | 7100007 | Lupus nephritis |
| Charlson Score | M321 | 8830137 | SLE fundus |
| Charlson Score | M321 | 8836513 | Respiratory disorder in systemic lupus erythematosus |
| Charlson Score | M321 | 8836515 | Pericarditis in systemic lupus erythematosus |
| Charlson Score | M321 | 8836516 | Cerebral arteritis in systemic lupus erythematosus |
| Charlson Score | M321 | 8836518 | Myelitis in systemic lupus erythematosus |
| Charlson Score | M321 | 8836519 | Encephalitis in systemic lupus erythematosus |
| Charlson Score | M321 | 8836520 | Encephalomyelitis in systemic lupus erythematosus |
| Charlson Score | M321 | 8840979 | Libman-Sacks endocarditis |
| Charlson Score | M321 | 8841438 | Myopathy in systemic lupus erythematosus |
| Charlson Score | M321 | 8844080 | Central nervous system lupus |
| Charlson Score | M321 | 8844339 | Lupus pleuritis |
| Charlson Score | M321 | 8844340 | Lupus enteritis |
| Charlson Score | M321 | 8844341 | Lupus pneumonia |
| Charlson Score | M321 | 8844342 | Lupus cystitis |
| Charlson Score | M321 | 8848278 | Interstitial pneumonitis in systemic lupus erythematosus |
| Charlson Score | M321 | 8850360 | Lupus peritonitis |
| Charlson Score | M329 | 7100011 | Systemic lupus erythematosus |
| Charlson Score | M329 | 8842174 | Lupus miliaris disseminatus faciei |
| Charlson Score | M329 | 8846167 | Steroid-resistant systemic lupus erythematosus |
| Charlson Score | M332 | 7104004 | Polymyositis |
| Charlson Score | M332 | 8837013 | Respiratory disorder in polymyositis |
| Charlson Score | M332 | 8848283 | Interstitial pneumonia in polymyositis |
| Charlson Score | M340 | 7101012 | Systemic scleroderma |
| Charlson Score | M341 | 8832845 | CREST syndrome |
| Charlson Score | M348 | 8837541 | Thibierge-Weissenbach syndrome |
| Charlson Score | M348 | 8841439 | Scleroderma myopathy |
| Charlson Score | M348 | 8844510 | Respiratory disorder in systemic scleroderma |
| Charlson Score | M348 | 8846114 | Scleroderma kidney |
| Charlson Score | M348 | 8846115 | Scleroderma renal crisis |
| Charlson Score | M349 | 7101001 | Scleroderma |
| Charlson Score | M353 | 7250004 | Polymyalgia rheumatica |

| **Category** | **ICD10** | **Diagnosis Code** | **Diagnosis Name** |
| --- | --- | --- | --- |
| Charlson Score | N012 | 8832474 | Rapidly progressive diffuse membranous glomerulonephritis |
| Charlson Score | N014 | 8832472 | Rapidly progressive diffuse endocapillary proliferative glomerulonephritis |
| Charlson Score | N016 | 8832471 | Rapidly progressive dense deposit disease |
| Charlson Score | N017 | 8832473 | Rapidly progressive diffuse crescentic glomerulonephritis |
| Charlson Score | N017 | 8848109 | ANCA-associated glomerulonephritis |
| Charlson Score | N017 | 8848133 | Rapidly progressive anti-GBM antibody-type glomerulonephritis |
| Charlson Score | N017 | 8848401 | Idiopathic crescentic glomerulonephritis |
| Charlson Score | N017 | 8848508 | Anti-glomerular basement membrane nephritis |
| Charlson Score | N019 | 8832470 | Rapidly progressive glomerulonephritis |
| Charlson Score | N030 | 8832919 | Mild chronic nephritic syndrome |
| Charlson Score | N032 | 8840393 | Chronic diffuse membranous glomerulonephritis |
| Charlson Score | N033 | 8840394 | Chronic diffuse mesangial proliferative glomerulonephritis |
| Charlson Score | N034 | 8840391 | Chronic diffuse endocapillary proliferative glomerulonephritis |
| Charlson Score | N036 | 8840376 | Chronic dense deposit disease |
| Charlson Score | N037 | 8840392 | Chronic diffuse crescentic glomerulonephritis |
| Charlson Score | N039 | 5829003 | Chronic glomerulonephritis |
| Charlson Score | N039 | 5829008 | Chronic nephritic syndrome |
| Charlson Score | N052 | 5831004 | Membranous nephropathy |
| Charlson Score | N052 | 8840228 | Membranous glomerulonephritis |
| Charlson Score | N053 | 8840538 | Mesangial proliferative glomerulonephritis |
| Charlson Score | N054 | 8831660 | Endocapillary proliferative glomerulonephritis |
| Charlson Score | N055 | 8840229 | Membranoproliferative glomerulonephritis |
| Charlson Score | N055 | 8840230 | Type 1 membranoproliferative glomerulonephritis |
| Charlson Score | N055 | 8840232 | Type 3 membranoproliferative glomerulonephritis |
| Charlson Score | N055 | 8849712 | Primary membranoproliferative glomerulonephritis |
| Charlson Score | N056 | 8840231 | Type 2 membranoproliferative glomerulonephritis |
| Charlson Score | N181 | 8847577 | Chronic kidney disease stage 1 |
| Charlson Score | N182 | 8847578 | Chronic kidney disease stage 2 |
| Charlson Score | N183 | 8847579 | Chronic kidney disease stage 3 |
| Charlson Score | N183 | 8847580 | Chronic kidney disease stage 3a |
| Charlson Score | N183 | 8847581 | Chronic kidney disease stage 3b |
| Charlson Score | N184 | 8847582 | Chronic kidney disease stage 4 |
| Charlson Score | N185 | 3621014 | Renal retinopathy |
| Charlson Score | N185 | 5869015 | Pericarditis uremic |
| Charlson Score | N185 | 5869016 | Uremic lung |
| Charlson Score | N185 | 8838554 | Uremic polyneuropathy |
| Charlson Score | N185 | 8838555 | Uremic neuropathy |
| Charlson Score | N185 | 8841385 | Uremic encephalopathy |
| Charlson Score | N185 | 8842116 | End-stage renal failure |

| **Category** | **ICD10** | **Diagnosis Code** | **Diagnosis Name** |
| --- | --- | --- | --- |
| Charlson Score | N185 | 8847501 | Anemia in ESA hyporesponsiveness |
| Charlson Score | N185 | 8847544 | Uremic cardiomyopathy |
| Charlson Score | N185 | 8847583 | Chronic kidney disease stage 5 |
| Charlson Score | N185 | 8848103 | Chronic kidney disease stage 5D |
| Charlson Score | N189 | 5859002 | Chronic renal failure |
| Charlson Score | N189 | 8844106 | Chronic kidney disease |
| Charlson Score | N19 | 2858001 | Renal anemia |
| Charlson Score | N19 | 5859001 | Uremia |
| Charlson Score | N19 | 5860004 | Renal anuria |
| Charlson Score | N19 | 5939017 | Non-functioning kidney |
| Charlson Score | N19 | 8835642 | Renal failure |
| Charlson Score | N19 | 8837198 | Proteinuria anemia |
| Charlson Score | N250 | 5880002 | Renal osteodystrophy |
| Charlson Score | N250 | 8835613 | Renal rickets |
| Charlson Score | N250 | 8835615 | Renal osteodystrophy |
| Charlson Score | N250 | 8841309 | Phosphate-losing tubular disorder |
| Charlson Score | N250 | 8847972 | Renal osteomalacia |
| Charlson Score | N251 | 5881001 | Nephrogenic diabetes insipidus |
| Charlson Score | N251 | 8848582 | Congenital nephrogenic diabetes insipidus |
| Charlson Score | N258 | 2762015 | Renal tubular acidosis |
| Charlson Score | N258 | 8830501 | Primary proximal renal tubular acidosis |
| Charlson Score | N258 | 8830899 | Distal renal tubular acidosis |
| Charlson Score | N258 | 8832721 | Proximal renal tubular acidosis |
| Charlson Score | N258 | 8833311 | Primary renal tubular acidosis |
| Charlson Score | N258 | 8838396 | Secondary proximal renal tubular acidosis |
| Charlson Score | N258 | 8838517 | Type 1 renal tubular acidosis |
| Charlson Score | N258 | 8838518 | Type 2 renal tubular acidosis |
| Charlson Score | N258 | 8838519 | Type 3 renal tubular acidosis |
| Charlson Score | N258 | 8840870 | Lightwood-Albright syndrome |
| Charlson Score | N258 | 8844495 | Nephrogenic secondary hyperparathyroidism |
| Charlson Score | N258 | 8847502 | Prolonged hyperparathyroidism |
| Charlson Score | N259 | 8832417 | Acute tubular disorder |
| Charlson Score | R02 | 7854001 | Congestive gangrene |
| Charlson Score | R02 | 7854002 | Lower-extremity gangrene |
| Charlson Score | R02 | 7854003 | Lower-extremity toe gangrene |
| Charlson Score | R02 | 7854004 | Lower-leg necrosis |
| Charlson Score | R02 | 7854005 | Lower-leg gangrene |
| Charlson Score | R02 | 7854008 | Necrotic ulcer |
| Charlson Score | R02 | 7854012 | Auricular gangrene |
| Charlson Score | R02 | 7854015 | Neurogenic gangrene |
| Charlson Score | R02 | 7854017 | Spinal gangrene |
| Charlson Score | R02 | 7854018 | Foot gangrene |
| Charlson Score | R02 | 7854022 | Ulcerative gangrene |
| Charlson Score | R02 | 7854024 | Idiopathic gangrene |
| Charlson Score | R02 | 7854027 | Skin necrosis |
| Charlson Score | R02 | 7854030 | Peripheral necrosis |
| Charlson Score | R02 | 7854033 | Toe necrosis |
| Charlson Score | R02 | 7854035 | Nodular necrotic dermatitis |
| Charlson Score | R02 | 7854036 | Necroinflammation |

| **Category** | **ICD10** | **Diagnosis Code** | **Diagnosis Name** |
| --- | --- | --- | --- |
| Charlson Score | R02 | 7854037 | Cutaneous gangrene |
| Charlson Score | R02 | 8830823 | Perineal gangrene |
| Charlson Score | R02 | 8830871 | Gangrene |
| Charlson Score | R02 | 8834388 | Toe gangrene |
| Charlson Score | R02 | 8834604 | Finger gangrene |
| Charlson Score | R02 | 8835664 | Edematous gangrene |
| Charlson Score | R02 | 8837923 | Thumb gangrene |
| Charlson Score | R02 | 8839748 | Abdominal wall gangrene |
| Charlson Score | R02 | 8848844 | Necrosis |
| Charlson Score | R02 | 8848949 | Foot necrosis |
| Charlson Score | R02 | 9951013 | Angioneurotic gangrene |
| Charlson Score | Z958 | 8842931 | Artificial blood vessel post-implantation |
| Charlson Score | Z958 | 8844497 | Stent implantation status |
| Charlson Score | Z958 | 8846270 | Carotid artery stent implantation status |
| Charlson Score | Z958 | 8847553 | Abdominal aortic stent implantation status |
| Charlson Score | Z958 | 8848122 | Indwelling condition of inferior vena cava filter |
