# Supplementary material for: Validation study of case-identifying algorithms for severe hypoglycemia using hospital administrative data in Japan
Source: PLoS One. 2023 Aug 9;18(8):e0289840. doi: 10.1371/journal.pone.0289840 (PMC10411751; doi:10.1371/journal.pone.0289840)
Supplement: S4 Table — (DOCX) [file pone.0289840.s005.docx]

**S4 Table. List of drug codes**

| **ATC** | **Health Insurance Claims Code** | **Name of Health Insurance Claims Code** |
| --- | --- | --- |
| K01C1 | 620001315 | Otsuka Glucose Solution 50% 200 mL |
| K01C1 | 620001316 | Otsuka Glucose Solution 50% 500 mL |
| K01C1 | 620002261 | 20% Glucose Injection Syringe (NP) 20 mL |
| K01C1 | 620002598 | Glucose Injection 20% Syringe (Terumo) 20 mL |
| K01C1 | 620002599 | Glucose Injection 50% Syringe (Terumo) 20 mL |
| K01C1 | 620004173 | Purified Glucose Solution 20% 500 mL |
| K01C1 | 620004174 | Purified Glucose Solution 30% 500 mL |
| K01C1 | 620004332 | Glucose Injection (Woozi) 20% 20 mL |
| K01C1 | 620004841 | Glucose Injection 40% - PL 20 mL |
| K01C1 | 620006270 | Terumo Glucose Injection 70% 350 mL |
| K01C1 | 620006636 | Terumo Glucose Injection 50% 200 mL |
| K01C1 | 620006637 | Terumo Glucose Injection 50% 500 mL |
| K01C1 | 620006647 | Glucose Injection 20% PL (Fuso) 20 mL |
| K01C1 | 620006648 | Glucose Injection 40% PL (Fuso) 20 mL |
| K01C1 | 620006649 | Glucose Injection 50% PL (Fuso) 20 mL |
| K01C1 | 620008378 | Glucose Injection 20% (Daiichi Sankyo) 20 mL |
| K01C1 | 620008379 | Glucose Injection 50% (Daiichi Sankyo) 20 mL |
| K01C1 | 620155611 | Glucose Injection 20% (NP) 20 mL |
| K01C1 | 620155634 | Purified Glucose Solution 20% 20 mL |
| K01C1 | 620155640 | Glucose Injection 20% (Mylan) 20 mL |
| K01C1 | 620155641 | Glucose Injection 20% PL (Hp) 20 mL |
| K01C1 | 620155642 | Glucose Injection 20% (TX) 20 mL |
| K01C1 | 620155645 | Glucose Injection 20% (AFP) 20 mL |
| K01C1 | 620155714 | Purified Glucose Solution 50% 20 mL |
| K01C1 | 620155716 | Glucose Injection 50% (AFP) 20 mL |
| K01C1 | 621651401 | Glucose Injection 20% Syringe (NP) 20 mL |
| K01C1 | 640406071 | Glucose Injection 20% - PL 20 mL |
| K01C1 | 640406124 | Glucose MP 20% 20 mL |
| K01C1 | 640408013 | 20% Glucose Injection (Nissin) 20 mL |
| K01C1 | 640412069 | Glucose Injection 50% 200 mL |
| K01C1 | 640412070 | Glucose Injection 50% 500 mL |
| K01C1 | 640412071 | Glucose Injection 70% 350 mL |
| K01C1 | 640421002 | 20% Glucose Injection (Nichiiko) 20 mL |
| K01C1 | 640421003 | Glucose Injection T 20% 20 mL |
| K01C1 | 640421004 | Terumo Glucose Injection 50 50% 200 mL |
| K01C1 | 640421005 | Terumo Glucose Injection 50 50% 500 mL |
| K01C1 | 640421006 | Terumo Glucose Injection 70 70% 350 mL |
| K01C1 | 640431008 | 20% Glucose Injection SN 20 mL |
| K01C1 | 640431047 | Glucose Injection 20% (CMX) 20 mL |
| K01C1 | 640460005 | Glucose Injection 50% - PL 20 mL |
| K01C1 | 640460006 | 50% Glucose Injection (Nissin) 20 mL |
| K01C1 | 643230042 | Glucose Injection 20% 20 mL |
| K01C1 | 643230045 | Glucose Injection 20% 500 mL |
| K01C1 | 643230046 | Glucose Injection 30% 500 mL |
| K01C1 | 643230047 | Glucose Injection 40% 20 mL |
| K01C1 | 643230048 | Glucose Injection 50% 20 mL |
| K01C1 | 643230049 | Glucose Injection 50% 200 mL |
| K01C1 | 643230050 | Glucose Injection 50% 300 mL |
| K01C1 | 643230052 | Glucose Injection 50% 500 mL |
| K01C1 | 643230252 | Glucose Injection 50% 100 mL |
| K01C1 | 643230298 | Otsuka Glucose Solution 20% 20 mL |
| K01C1 | 643230301 | Kobayashi Glucose Solution 20% 20 mL |
| K01C1 | 643230302 | Daiichi Glucose Injection 20% 20 mL |
| K01C1 | 643230305 | Glucose Injection (Hishiyama) 20% 20 mL |
| K01C1 | 643230307 | Glucose Injection 20% (Isei) 20 mL |
| K01C1 | 643230308 | Glucose Injection 20% (Towa) 20 mL |
| K01C1 | 643230312 | Purified Glucose Solution 20% 500 mL |
| K01C1 | 643230313 | Purified Glucose Solution 30% 500 mL |
| K01C1 | 643230314 | Otsuka Glucose Solution 40% 20 mL |
| K01C1 | 643230317 | Otsuka Glucose Solution 50% 20 mL |
| K01C1 | 643230319 | Kobayashi Glucose Solution 50% 20 mL |
| K01C1 | 643230320 | Daiichi Glucose Injection 50% 20 mL |
| K01C1 | 643230324 | Purified Glucose Solution 50% 100 mL |
| K01C1 | 643230328 | Purified Glucose Solution 50% 300 mL |
| K01C1 | 643230331 | Purified Glucose Solution 50% 500 mL |
| K01C1 | 643230412 | Otsuka Glucose Solution 70% 350 mL |
