# Supplementary material for: Validation study of case-identifying algorithms for severe hypoglycemia using hospital administrative data in Japan
Source: PLoS One. 2023 Aug 9;18(8):e0289840. doi: 10.1371/journal.pone.0289840 (PMC10411751; doi:10.1371/journal.pone.0289840)
Supplement: S5 Table — (DOCX) [file pone.0289840.s006.docx]

**S5 Table. Analysis for hospital selection**

|  | **Eligible patients in MDV database^a^**  **(N＝150,671)** | **Eligible patients at participating hospitals^b^**  **(N＝757)** | **Hospital 1^c^**  **(N＝339)** | **Hospital 2^c^**  **(N＝185)** | **Hospital 3^c^**  **(N＝233)** |
| --- | --- | --- | --- | --- | --- |
| **Age group**, % |  |  |  |  |  |
| <39 years | 3.6 | 3.4 | 2.9 | 3.8 | 3.9 |
| 40-64 years | 22.1 | 22.6 | 23.3 | 24.3 | 20.2 |
| 65-74 years | 26.8 | 26.4 | 24.5 | 27.0 | 28.8 |
| 75-84 years | 31.7 | 31.7 | 35.1 | 26.5 | 30.9 |
| ≥85 years | 15.8 | 15.9 | 14.2 | 18.4 | 16.3 |
| **Hospitalization category**, % |  |  |  |  |  |
| Inpatient | 48.0 | 50.1 | 49.3 | 51.4 | 50.2 |
| Outpatient | 52.0 | 49.9 | 50.7 | 48.6 | 49.8 |

^a^Number of eligible patients extracted from the whole MDV database.

^b^Sum of the numbers of eligible patients from each hospital in the MDV database.

**^c^**Per agreement with the hospitals to maintain their anonymity, participating hospitals have been arbitrarily numbered here as 1, 2, and 3. This sequence does not correspond to the order or timing of the eligibility evaluation periods.

MDV = Medical Data Vision
