# Supplementary material for: Validation study of case-identifying algorithms for severe hypoglycemia using hospital administrative data in Japan
Source: PLoS One. 2023 Aug 9;18(8):e0289840. doi: 10.1371/journal.pone.0289840 (PMC10411751; doi:10.1371/journal.pone.0289840)
Supplement: S6 Table — (DOCX) [file pone.0289840.s007.docx]

**S6 Table. Performance metrics of case-identifying algorithms for severe hypoglycemia (all evaluated algorithms)**

| **Case definition^a^** | **Cases with target condition^b,c^** | **Index test positive^d^** | **True positive^e^** | **PPV^f,g^**  **(95% CI)** | **Sensitivity^g,h^**  **(95% CI)** | **Prev^i^** | **Prob+^j^** | **Prob-^k^** | |
| --- | --- | --- | --- | --- | --- | --- | --- | --- | --- |
| 1. Any diagnoses (including suspected diagnoses) listed in footnote (a) recorded or high concentration glucose (i.e., ≥20% prescribed at the index date. | 72 | 260 | 72 | 0.277  (0.223, 0.331) | 1.000  (1.000, 1.000) | 0.277 | 1.000 | 0 |  |
| 2. Any diagnoses (excluding suspected diagnoses) listed in footnote (a) recorded or high concentration glucose (i.e., ≥20%) prescribed at the index date. | 72 | 206 | 71 | 0.345  (0.280, 0.410) | 0.986  (0.959, 1.013) | 0.277 | 0.792 | 0.208 |  |
| 3. Any diagnoses (including suspected diagnoses) listed in footnote (a) recorded at the index date. | 72 | 149 | 49 | 0.329  (0.253, 0.404) | 0.681  (0.573, 0.788) | 0.277 | 0.573 | 0.427 |  |
| 4.Any diagnoses (excluding suspected diagnoses) listed in footnote (a) recorded at the index date. | 72 | 94 | 47 | 0.500  (0.399, 0.601) | 0.653  (0.543, 0.763) | 0.277 | 0.362 | 0.638 |  |
| 5.Any diagnoses (including suspected diagnoses) listed in footnote (a) recorded and high concentration glucose (i.e., ≥20%) prescribed at the index date. | 72 | 36 | 28 | 0.778  (0.642, 0.914) | 0.389  (0.276, 0.501) | 0.277 | 0.138 | 0.862 |  |
| 6. Any diagnoses (excluding suspected diagnoses) listed in footnote (a) recorded and high concentration glucose (i.e., ≥20%) prescribed at the index date. | 72 | 35 | 27 | 0.771  (0.632, 0.911) | 0.375  (0.263, 0.487) | 0.277 | 0.135 | 0.865 |  |
| 7. Diagnosis information in algorithm 1 limited to one of E10.0, E11.0, E14.0. | 72 | 152 | 51 | 0.336  (0.260, 0.411) | 0.708  (0.603, 0.813) | 0.277 | 0.585 | 0.415 |  |
| 8. Diagnosis information in algorithm 2 limited to one of E10.0, E11.0, E14.0. | 72 | 152 | 51 | 0.336  (0.260, 0.411) | 0.708  (0.603, 0.813) | 0.277 | 0.585 | 0.415 |  |
| 9. Diagnosis information in algorithm 3 limited to one of E10.0, E11.0, E14.0. | 72 | 9 | 3 | 0.333  (0.025, 0.641) | 0.042  (-0.004, 0.088) | 0.277 | 0.035 | 0.965 |  |
| 10. Diagnosis information in algorithm 4 limited to one of E10.0, E11.0, E14.0. | 72 | 9 | 3 | 0.333  (0.025, 0.641) | 0.042  (-0.004, 0.088) | 0.277 | 0.035 | 0.965 |  |
| 11. Diagnosis information in algorithm 5 limited to one of E10.0, E11.0, E14.0. | 72 | 4 | 3 | 0.750  (0.326, 1.174) | 0.042  (-0.004, 0.088) | 0.277 | 0.154 | 0.985 |  |
| 12. Diagnosis information in algorithm 6 limited to one of E10.0, E11.0, E14.0. | 72 | 4 | 3 | 0.750  (0.326, 1.174) | 0.042  (-0.004, 0.088) | 0.277 | 0.154 | 0.985 |  |
| 13. Diagnosis information in algorithm 1 limited to one of E10.0, E11.0, E14.0, E16.2. | 72 | 229 | 69 | 0.301  (0.242, 0.361) | 0.958  (0.912, 1.004) | 0.277 | 0.881 | 0.119 |  |
| 14. Diagnosis information in algorithm 2 limited to one of E10.0, E11.0, E14.0, E16.2. | 72 | 198 | 68 | 0.343  (0.277, 0.410) | 0.944  (0.892, 0.997) | 0.277 | 0.762 | 0.238 |  |
| 15. Diagnosis information in algorithm 3 limited to one of E10.0, E11.0, E14.0, E16.2. | 72 | 115 | 43 | 0.374  (0.285, 0.462) | 0.597  (0.484, 0.711) | 0.277 | 0.442 | 0.558 |  |
| 16. Diagnosis information in algorithm 4 limited to one of E10.0, E11.0, E14.0, E16.2. | 72 | 83 | 41 | 0.494  (0.386, 0.602) | 0.569  (0.455, 0.684) | 0.277 | 0.319 | 0.681 |  |
| 17. Diagnosis information in algorithm 5 limited to one of E10.0, E11.0, E14.0, E16.2. | 72 | 33 | 25 | 0.758  (0.611, 0.904) | 0.347  (0.237, 0.457) | 0.277 | 0.127 | 0.873 |  |
| 18. Diagnosis information in algorithm 6 limited to one of E10.0, E11.0, E14.0, E16.2. | 72 | 32 | 24 | 0.750  (0.600, 0.900) | 0.333  (0.224, 0.442) | 0.277 | 0.123 | 0.877 |  |
| 19. Diagnosis information in algorithm 1 limited to E10.0. | 72 | 147 | 51 | 0.347  (0.270, 0.424) | 0.708  (0.603, 0.813) | 0.277 | 0.565 | 0.435 |  |
| 20. Diagnosis information in algorithm 2 limited to E10.0. | 72 | 147 | 51 | 0.347  (0.270, 0.424) | 0.708  (0.603, 0.813) | 0.277 | 0.565 | 0.435 |  |
| 21. Diagnosis information in algorithm 3 limited to E10.0. | 72 | 1 | 1 | 1.000  (1.000, 1.000) | 0.014  (-0.013, 0.041) | 0.277 | 0.004 | 0.996 |  |
| 22. Diagnosis information in algorithm 4 limited to E10.0. | 72 | 1 | 1 | 1.000  (1.000, 1.000) | 0.014  (-0.013, 0.041) | 0.277 | 0.004 | 0.996 |  |
| 23. Diagnosis information in algorithm 5 limited to E10.0. | 72 | 1 | 1 | 1.000  (1.000, 1.000) | 0.014  (-0.013, 0.041) | 0.277 | 0.004 | 0.996 |  |
| 24. Diagnosis information in algorithm 6 limited to E10.0. | 72 | 1 | 1 | 1.000  (1.000, 1.000) | 0.014  (-0.013, 0.041) | 0.277 | 0.004 | 0.996 |  |
| 25. Diagnosis information in algorithm 1 limited to E11.0. | 72 | 152 | 51 | 0.336  (0.260, 0.411) | 0.708  (0.603, 0.813) | 0.277 | 0.585 | 0.415 |  |
| 26. Diagnosis information in algorithm 2 limited to E11.0. | 72 | 152 | 51 | 0.336  (0.260, 0.411) | 0.708  (0.603, 0.813) | 0.277 | 0.585 | 0.415 |  |
| 27. Diagnosis information in algorithm 3 limited to E11.0. | 72 | 7 | 2 | 0.286  (-0.049, 0.620) | 0.028  (-0.010, 0.066) | 0.277 | 0.027 | 0.973 |  |
| 28. Diagnosis information in algorithm 4 limited to E11.0. | 72 | 7 | 2 | 0.286  (-0.049, 0.620) | 0.028  (-0.010, 0.066) | 0.277 | 0.027 | 0.973 |  |
| 29. Diagnosis information in algorithm 5 limited to E11.0. | 72 | 2 | 2 | 1.000  (1.000, 1.000) | 0.028  (-0.010, 0.066) | 0.277 | 0.008 | 0.992 |  |
| 30. Diagnosis information in algorithm 6 limited to E11.0. | 72 | 2 | 2 | 1.000  (1.000, 1.000) | 0.028  (-0.010, 0.066) | 0.277 | 0.008 | 0.992 |  |
| 31. Diagnosis information in algorithm 1 limited to E14.0. | 72 | 148 | 51 | 0.345  (0.268, 0.421) | 0.708  (0.603, 0.813) | 0.277 | 0.569 | 0.431 |  |
| 32. Diagnosis information in algorithm 2 limited to E14.0. | 72 | 148 | 51 | 0.345  (0.268, 0.421) | 0.708  (0.603, 0.813) | 0.277 | 0.569 | 0.431 |  |
| 33. Diagnosis information in algorithm 3 limited to E14.0. | 72 | 2 | 0 | 0  (0, 0) | 0  (0, 0) | 0.277 | 0.008 | 0.992 |  |
| 34. Diagnosis information in algorithm 4 limited to E14.0. | 72 | 2 | 0 | 0  (0, 0) | 0  (0, 0) | 0.277 | 0.008 | 0.992 |  |
| 35. Diagnosis information in algorithm 5 limited to E14.0. | 72 | 1 | 0 | 0  (0, 0) | 0  (0, 0) | 0.277 | 0.004 | 0.996 |  |
| 36. Diagnosis information in algorithm 6 limited to E14.0. | 72 | 1 | 0 | 0  (0, 0) | 0  (0, 0) | 0.277 | 0.004 | 0.996 |  |
| 37. Diagnosis information in algorithm 1 limited to E15. | 72 | 149 | 53 | 0.356  (0.279, 0.433) | 0.736  (0.634, 0.838) | 0.277 | 0.573 | 0.427 |  |
| 38. Diagnosis information in algorithm 2 limited to E15. | 72 | 149 | 53 | 0.356  (0.279, 0.433) | 0.736  (0.634, 0.838) | 0.277 | 0.573 | 0.427 |  |
| 39. Diagnosis information in algorithm 3 limited to E15. | 72 | 4 | 4 | 1.000  (1.000, 1.000) | 0.056  (0.003, 0.108) | 0.277 | 0.015 | 0.985 |  |
| 40. Diagnosis information in algorithm 4 limited to E15. | 72 | 4 | 4 | 1.000  (1.000, 1.000) | 0.056  (0.003, 0.108) | 0.277 | 0.015 | 0.985 |  |
| 41. Diagnosis information in algorithm 5 limited to E15. | 72 | 2 | 2 | 1.000  (1.000, 1.000) | 0.028  (-0.010, 0.066) | 0.277 | 0.008 | 0.992 |  |
| 42. Diagnosis information in algorithm 6 limited to E15. | 72 | 2 | 2 | 1.000  (1.000, 1.000) | 0.028  (-0.010, 0.066) | 0.277 | 0.008 | 0.992 |  |
| 43. Diagnosis information in algorithm 1 limited to E16.0. | 72 | 148 | 52 | 0.351  (0.274, 0.428) | 0.722  (0.619, 0.826) | 0.277 | 0.569 | 0.431 |  |
| 44. Diagnosis information in algorithm 2 limited to E16.0. | 72 | 148 | 52 | 0.351  (0.274, 0.428) | 0.722  (0.619, 0.826) | 0.277 | 0.569 | 0.431 |  |
| 45. Diagnosis information in algorithm 3 limited to E16.0. | 72 | 2 | 2 | 1.000  (1.000, 1.000) | 0.028  (-0.010, 0.066) | 0.277 | 0.008 | 0.992 |  |
| 46. Diagnosis information in algorithm 4 limited to E16.0. | 72 | 2 | 2 | 1.000  (1.000, 1.000) | 0.028  (-0.010, 0.066) | 0.277 | 0.008 | 0.992 |  |
| 47. Diagnosis information in algorithm 5 limited to E16.0. | 72 | 1 | 1 | 1.000  (1.000, 1.000) | 0.014  (-0.013, 0.041) | 0.277 | 0.004 | 0.996 |  |
| 48. Diagnosis information in algorithm 6 limited to E16.0. | 72 | 1 | 1 | 1.000  (1.000, 1.000) | 0.014  (-0.013, 0.041) | 0.277 | 0.004 | 0.996 |  |
| 49. Diagnosis information in algorithm 1 limited to E16.1. | 72 | 175 | 51 | 0.291  (0.224, 0.359) | 0.708  (0.603, 0.813) | 0.277 | 0.673 | 0.327 |  |
| 50. Diagnosis information in algorithm 2 limited to E16.1. | 72 | 152 | 51 | 0.336  (0.260, 0.411) | 0.708  (0.603, 0.813) | 0.277 | 0.585 | 0.415 |  |
| 51. Diagnosis information in algorithm 3 limited to E16.1. | 72 | 28 | 0 | 0  (0, 0) | 0  (0, 0) | 0.277 | 0.108 | 0.892 |  |
| 52. Diagnosis information in algorithm 4 limited to E16.1. | 72 | 5 | 0 | 0  (0, 0) | 0  (0, 0) | 0.277 | 0.019 | 0.981 |  |
| 53. Diagnosis information in algorithm 5 limited to E16.1. | 72 | 0 | 0 | -- | 0  (0, 0) | 0.277 | 0 | 1 |  |
| 54. Diagnosis information in algorithm 6 limited to E16.1. | 72 | 0 | 0 | -- | 0  (0, 0) | 0.277 | 0 | 1 |  |
| 55. Diagnosis information in algorithm 1 limited to E16.2. | 72 | 224 | 69 | 0.308  (0.248, 0.369) | 0.958  (0.912, 1.004) | 0.277 | 0.862 | 0.138 |  |
| 56. Diagnosis information in algorithm 2 limited to E16.2. | 72 | 193 | 68 | 0.352  (0.285, 0.420) | 0.944  (0.891, 0.997) | 0.277 | 0.742 | 0.258 |  |
| 57. Diagnosis information in algorithm 3 limited to E16.2. | 72 | 107 | 41 | 0.383  (0.291, 0.475) | 0.569  (0.455, 0.684) | 0.277 | 0.412 | 0.588 |  |
| 58. Diagnosis information in algorithm 4 limited to E16.2. | 72 | 75 | 39 | 0.520  (0.407, 0.633) | 0.542  (0.427, 0.657) | 0.277 | 0.288 | 0.712 |  |
| 59. Diagnosis information in algorithm 5 limited to E16.2. | 72 | 30 | 23 | 0.767  (0.615, 0.918) | 0.319  (0.212, 0.427) | 0.277 | 0.115 | 0.885 |  |
| 60. Diagnosis information in algorithm 6 limited to E16.2. | 72 | 29 | 22 | 0.759  (0.603, 0.914) | 0.306  (0.199, 0.412) | 0.277 | 0.112 | 0.888 |  |
| 61. High-concentration glucose (i.e., ≥20%) prescribed at the index date | 72 | 147 | 51 | 0.347  (0.270, 0.424) | 0.708  (0.603, 0.813) | 0.277 | 0.565 | 0.435 |  |

^a^ICD-10 codes [24] corresponding to names of diseases for possible hypoglycemia: E10.0 = type 1 diabetes mellitus with coma; E11.0 = type 2 diabetes mellitus with coma; E14.0 = unspecified diabetes mellitus with coma; E15 = non-diabetic hypoglycemic coma; E16.0 = drug-induced hypoglycemia without coma; E16.1 = other hypoglycemia; E16.2 = hypoglycemia, unspecified.

^b^The total number of possible cases identified with the algorithm was 336, of which 260 were sampled for validation.

^c^Cases with the target condition were identified by medical record review as severe hypoglycemia and were the reference standard in this study. These cases may include both true positives and false negatives.

^d^Index test-positive cases met the criteria for each case-identifying algorithm and may include both true-positive cases and false-positive cases.

^e^True-positive cases met the criteria for each case-identifying algorithm and were identified as cases of severe hypoglycemia by medical record review.

^f^PPV was calculated based on the number of true-positive cases divided by the number of index test-positive cases (i.e., the sum of the number of true-positive and false-positive cases).

^g^95% CIs were calculated using normal approximation to the binomial distribution.

^h^Sensitivity was calculated by dividing the number of true-positive cases by the number of cases with the target condition (i.e., the sum of true-positive and false-negative cases) with the assumption that all cases with the target condition met the eligibility criteria and were included in the study population.

^i^Prevalence was calculated by dividing the sum of the true-positive and false-negative cases by the total number of cases.

^j^Probability+ was calculated by dividing the sum of the true-positive and false-positive cases by the total number of cases.

^k^Probability- was calculated by dividing the sum of the true-negative and falsenegative cases by the total number of cases.

CI = confidence interval; ICD-10 = International Statistical Classification of Diseases and Related Health Problems, 10^th^ revision; PPV = positive predictive value; Prev = prevalence; Prob = probability.
